# A general design approach toward covalent organic frameworks for highly efficient electrochemiluminescence

**DOI:** 10.1038/s41467-021-25013-8

**Published:** 2021-08-05

**Authors:** Ya-Jie Li, Wei-Rong Cui, Qiao-Qiao Jiang, Qiong Wu, Ru-Ping Liang, Qiu-Xia Luo, Jian-Ding Qiu

**Affiliations:** 1 College of Chemistry, Nanchang University, Nanchang, 330031 China; 2 College of Materials and Chemical Engineering, Pingxiang University, Pingxiang, 337055 China

**Keywords:** Polymers, Electronic properties and materials, Organic molecules in materials science

## Abstract

Electrochemiluminescence (ECL) plays a key role in analysis and sensing because of its high sensitivity and low background. Its wide applications are however limited by a lack of highly tunable ECL luminophores. Here we develop a scalable method to design ECL emitters of covalent organic frameworks (COFs) in aqueous medium by simultaneously restricting the donor and acceptor to the COFs’ tight electron configurations and constructing high-speed charge transport networks through olefin linkages. This design allows efficient intramolecular charge transfer for strong ECL, and no exogenous poisonous co-reactants are needed. Olefin-linked donor-acceptor conjugated COFs, systematically synthesized by combining non-ECL active monomers with C_2v_ or C_3v_ symmetry, exhibit strong ECL signals, which can be boosted by increasing the chain length and conjugation of monomers. The present concept demonstrates that the highly efficient COF-based ECL luminophores can be precisely designed, providing a promising direction toward COF-based ECL phosphors.

## Abstract (web summary)

Electrochemiluminescence (ECL) plays a key role in analysis and sensing but its application is limited by a lack of highly tunable luminophores. Here, the authors demonstrate the design of high efficient ECL luminophores of covalent organic frameworks (COFs) in aqueous media by simultaneously restricting the donor and acceptor to the COFs’ electron configurations and constructing charge transport networks through olefin linkages.

## Introduction

As the first discovery of electrochemiluminescence (ECL), ~100 years ago, this discipline has been developed from an academic curiosity to a powerful electroanalytical tool^1^. ECL is excited by electrochemical reactions rather than by photons, endowing this technique with unique advantages of low background and high sensitivity, high selectivity, fast response, and spatial and temporal control of luminescence^2–5^. Thus, ECL has been extensively explored in analytical fields of environment, clinical, medicine, and biology^4,6–10^.

However, the wide application of ECL is limited by the low quantum efficiency (Φ_ECL_) of the phosphors in aqueous media. The quantum efficiency of widely used inorganic phosphors such as [Ru(bpy)_3_]^2+^ and Ir(ppy)_3_ is difficult to be improved since their structures can hardly be modified. Although the organic phosphors (hydrocarbons^9–11^, rubrene^12^, and organic dyes^13–15^) are modifiable, they function only in aprotic solvents, as their ECL is significantly quenched in aqueous media by water and oxygen via either disabling reductive/oxidative ECL precursors or quenching the formed excited states^1^. In addition, practical application of these phosphors in the construction of ECL-based sensors requires the utilization of noxious co-reactants (e.g., tri-n-propylamine (TPrA)) to amplify the signals owing to the low quantum efficiency of the existed phosphors^16–18^. Thus, the development of water-compatible ECL phosphors with high quantum efficiency is urgent for wider applications. However, the reasonable design and synthesis are limited owing to the lack of an integrated tailor-made material platform.

Covalent organic frameworks (COFs), an emerging class of bottom–up crystalline porous polymers, are connected by covalent bonds of molecular building units with pre-designed geometric structures^19–21^. Their structures can be easily designed via the atomic precise assembly of organic components at the molecular level^22,23^. The past decade has witnessed the explosion of COFs’ applications in catalysis^24–26^, energy storage^27,28^, and optoelectronic devices^29–31^. We believe that the structural designability of COFs might enable systematically tunable ECL systems. However, most reported COFs are connected by polarized dynamic covalent bonds (such as imine bonds), showing relatively weak π-electronic connectivity on the skeleton^32,33^. Besides, the intramolecular rotation induced by π-π stacking and the non-radiative relaxation caused by self-quenching become the main obstacles for luminescent COFs^34,35^. Recently, Luo’s group constructed the ECL-COFs with aggregation-induced (AI)-ECL monomers, where ECL was observed using H_2_O_2_ as a co-reactant and Co_3_O_4_ as an amplification element^2^. Similarly, Yuan’s group observed ECL of COF nanosheet with co-reactant S_2_O_8_^2−^ and co-reaction accelerator Bu_4_NPF_6_, which topologically linked the aggregation-caused quenching ECL luminophores (pyrene) and AIE groups^8^. However, their COFs only had very low ECL signals. To date, the highly efficient ECL of COFs constructed by non-ECL monomers has not yet been reported. In addition, systematic induction and methodology research on COF-based ECL is lacking.

**Fig. 1:**
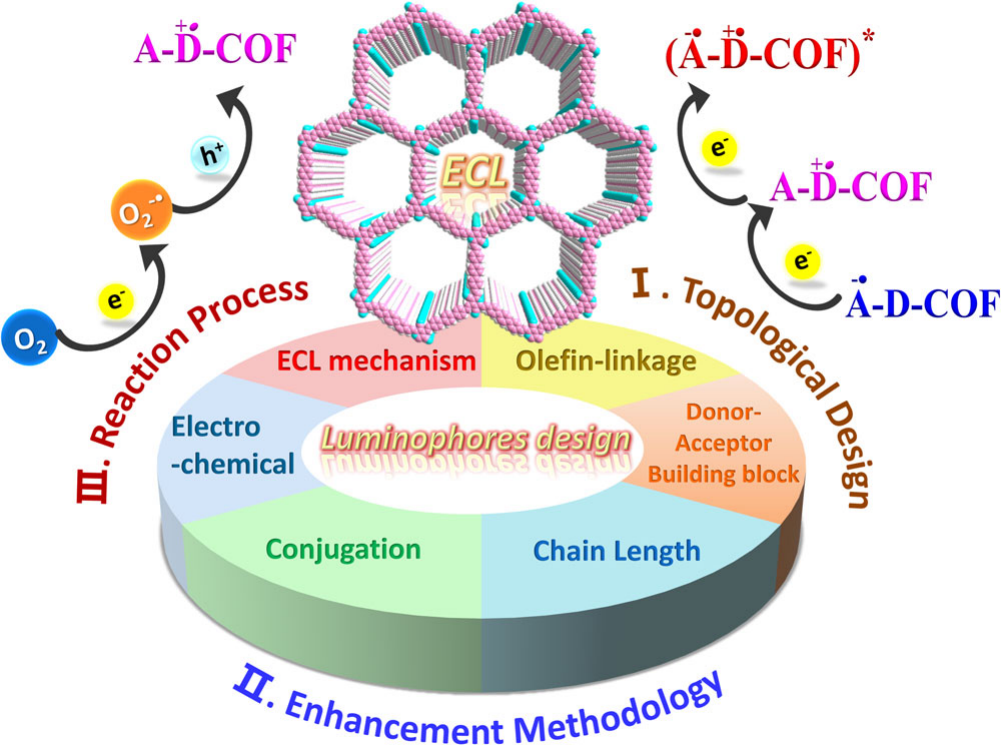
Mechanism diagram. The diagram shows a combinatorial strategy to solve the rapid annihilation of electron-donors and acceptors, facilitate intramolecular charge transfer (ICT) along with the olefin linkages, and thus activate the COF-ECL from the non-ECL active monomers. The aquatic oxygen as a substitute, external co-reactants are not needed. Some scalable design strategies and reaction processes of COFs were further expounded to improve ECL properties, aiming to inspiring researchers to find more ECL phosphors in practice.

To address the low conductivity and self-quenching of reported COFs, hereby, we report on the assembly of a series of olefin-linked donor-acceptor COFs as ECL phosphors by employing C_2v_ symmetrical monomer 2,4,6-trimethylpyridine-3,5-dicarbonitrile (DCTP) and C_3v_ symmetrical monomer 2,4,6-trimethyl-1,3,5-triazine (TMT) as two key acceptors subunits. Different linear and trigonal aldehyde monomers were used to ensure the generalizability of this concept. On the basis of ECL performance of the synthesized COFs, we summarized a scalable topological design protocol for efficient ECL-COFs in aqueous media with internal dissolved oxygen acting as a co-reactant (Fig. 1). It no longer needs the H_2_O_2_, S_2_O_8_^2−^, TPrA, and other highly oxidizing co-reactants, showing some additional attractive characteristics of green, environmental friendliness, and good biocompatibility. Then, the effects of the chain length and strengthening conjugate of the building blocks on the ECL intensity of the COFs were studied along with theoretical calculations. Finally, the structural related ECL properties and mechanism of the COFs have been clarified, which will provide guidelines for ECL molecular structure design and performance improvement.

## Results

### Topological design of ECL-activated COFs

**Fig. 2:**
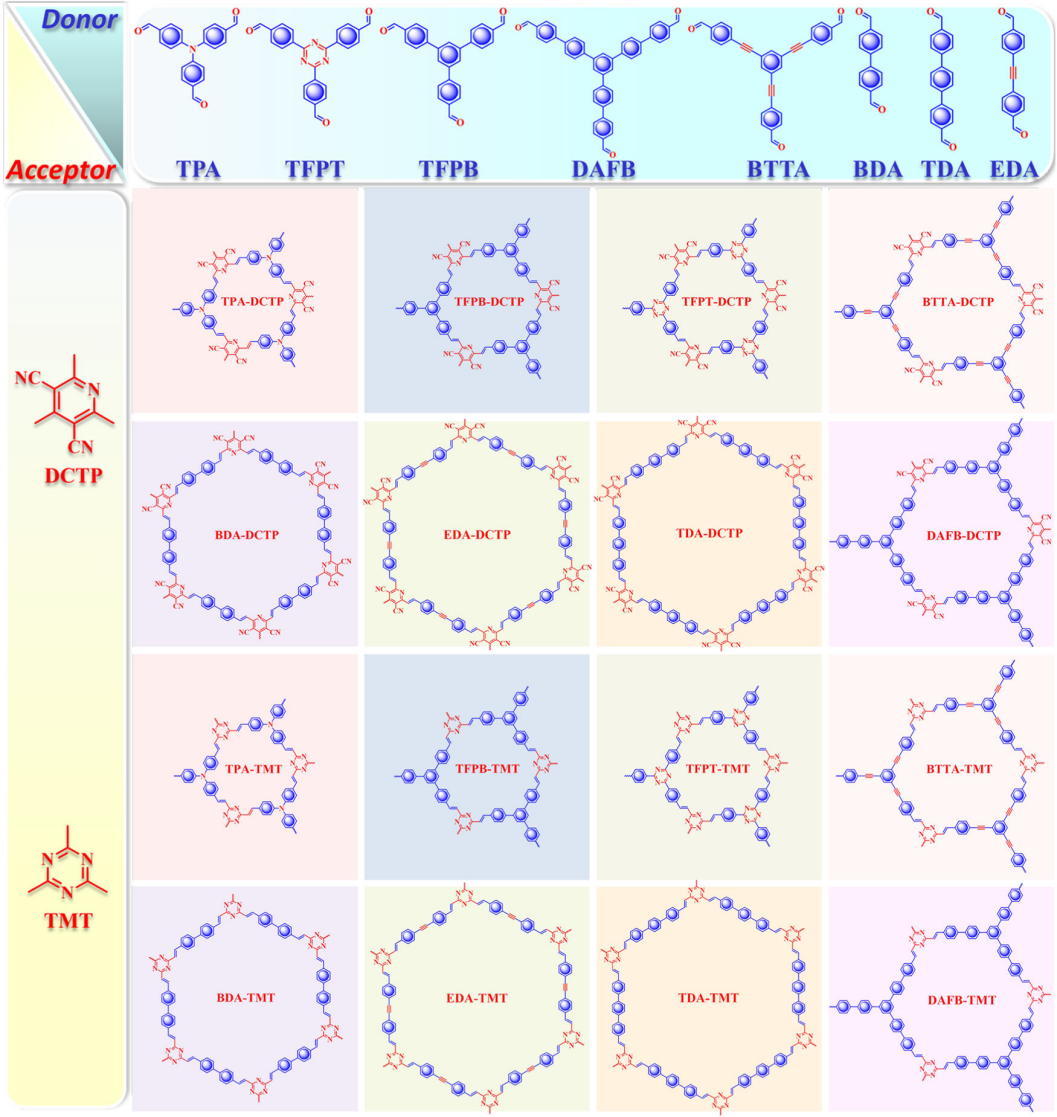
Chemical structures of synthesized COFs. Using two electron-deficient monomers as key receptors and eight electron-rich monomers as donors, 16 olefin-linked donor-acceptor COFs were synthesized.

To activate the ECL responses, COFs are constructed with various combinations of building blocks (Fig. 2). For a high versatility, the monomers with different symmetries, C_2v_ symmetric DCTP and C_3v_ symmetric TMT, are selected as two key subunits. Different configurations of aldehyde monomers are also used as another subunit. 4,4’-Biphenyldicarboxaldehyde (BDA), 4,4”-*p*-terphenyldicarboxaldehyde (TDA), and 4-[2-(4-formylphenyl)ethynyl]benzaldehyde (EDA) can be regarded as linear monomers. 4-[4-[3,5-Bis[4-(4-formylphenyl)phenyl]phenyl]phenyl]benzoic acid (DAFB), 4,4,4-(benzene-1,3,5-triyltris(ethyne-2,1-diyl))tribenzaldehyde (BTTA), 1,3,5-tris(4-formylphenyl)benzene (TFPB), tris(4-formylphenyl)amine (TPA), and 2,4,6-tris(4-formylphenyl)-1,3,5-triazine (TFPT) are representative trigonal monomers.

The olefin-linked COFs were synthesized via Knoevenagel condensation or aldol condensation reaction using piperidine (for DCTP-COFs) and trifluoroacetic acid (for TMT-COFs) as catalysts, respectively. The characterizations of all COFs and model compounds are described in the Supplementary Information (Supplementary Figs. 1–30). The models proposed for the TPA-DCTP, TFPT-DCTP, TFPB-DCTP, DAFB-DCTP, BTTA-DCTP, and DAFB-TMT series COFs are in *PM* space group, the BDA-DCTP, TDA-DCTP, and EDA-DCTP series COFs are in *P2/M* space group, the TPA-TMT, TFPT-TMT, TFPB-TMT, and BTTA-TMT series COFs are in *P6* space group, and the BDA-TMT, TDA-TMT, and EDA-TMT are in *P6/M* space group (Supplementary Tables 1–18). The morphological properties were also studied by the scanning electron microscope (SEM), which showed that all the COFs possess an analogous fibrillar crystal morphology, uniform diameter (~40 nm), and length of several micrometers (Supplementary Figs. 31–35).

As the charge dynamics in devices can be easily controlled, the D-A heterojunction is currently a key structure including transistors, light-emitting diodes, and photovoltaic technologies^36–39^. However, the donor and acceptor molecules always tend to pile up with each other rather than to separately form the bicontinuous arrays, in which charge carriers are captured and easily annihilated by rapid binding^38,39^. The periodically stacked 2D-COFs can solve this predicament of D-A-disordered arrangement and provide an independent route to allow the conduction of electrons and holes^38^. However, it is challenging to arrange the D and A molecules to co-crystallize and stack against each other to form periodic, D-on-D, and A-on-A columnar COF arrays^37^. To address these issues, we synthesized D-A conjugated olefin-linked COFs with spatially separated D-A structures and extended π-conjugated skeletons to demonstrate the tunability of the ECL of the resulted COFs.

**Fig. 3:**
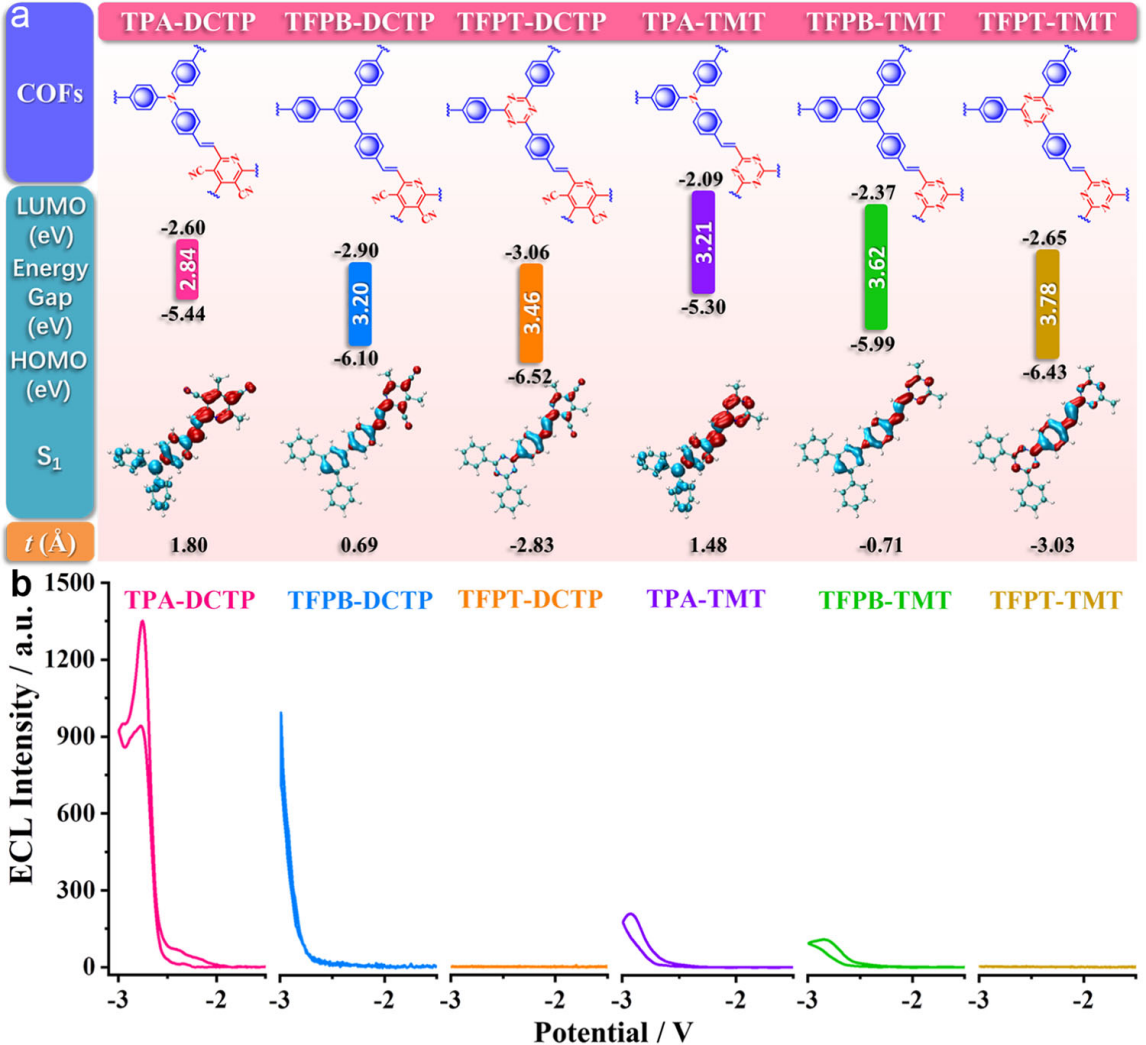
Influence of D-A in COFs on ECL performance. **a** DFT calculations to the building blocks of COFs. The values of HOMOs, LUMOs, and calculated energy gaps and analysis for the distribution of hole (blue) and electron (red) for S_1_ are indicated. The *t* index is measured to evaluate the separation degree of hole and electron. **b** Comparison of ECL intensities of COFs in 0.1 M PBS with 0.1 M KCl between 0 V and −3.0 V. PMT: 800 V, pH 7.5, potential scan rate: 100 mV s^−1^.

We introduced the possible auxiliary electron-donors TPA, TFPB, and TFPT into the HOMOs of DCTP and TMT within the COFs’ skeletons. To verify the construction of the D-A system, we extracted a building block of each COF as a model and calculated the molecular geometries of the ground state and excited state by B3LYP and TD-B3LYP methods, respectively. For TPA-DCTP, TFPB-DCTP, TPA-TMT, and TFPB-TMT, their HOMOs are located on the π-conjugated main chains of TPA/TFPB subunits as an electron-donors unit, whereas the LUMOs are concentrated on the DCTP/TMT subunits as acceptors (Supplementary Fig. 36), implying that the ICT occurs from the D to A in the whole molecule of COFs. After excitation, the electrons and holes are concentrated, respectively, on the receptors and donors moieties with effective separation, and the overlap of S_1_ molecular orbitals is reduced simultaneously (Fig. 3a, Supplementary Table 19). Through this D-A conjugated structure, the ICTs formed within the molecules can effectively activate the ECL signals of COFs (Fig. 3b).

A narrower bandgap is often conducive to a higher ECL intensity^1,40^. Besides the above HOMO-LUMO bandgaps calculated from the building blocks, the bandgaps of COFs were also determined through the Kubelka–Munk-transformed reflectance spectra, which are observed to decrease by 1.01~1.25 eV when COFs are formed with extended π-conjugated skeletons from the organic building blocks. It is noteworthy that the TPA-COFs (TPA-DCTP, TPA-TMT), with stronger electron-supplying abilities, possess smaller bandgaps of 1.82 eV, 2.08 eV and emerge ~1.4 times stronger ECL intensities than those of TFPB-COFs (TFPB-DCTP, TFPB-TMT) (Fig. 3b and Supplementary Fig. 37). For further quantitatively evaluating the ICT characteristics, the excited state of each COF-building block was analyzed by Multiwfn, and the related charge transfer parameters are listed in Supplementary Table 19. The *t* index, which demonstrates the degree of separation between holes and electrons, is also shown as a representative in Fig. 3a. The TFPT monomer has the lowest HOMO energy level (Supplementary Figs. 38 and 39), showing the weakest electron-donor ability. When TFPT-DCTP and TFPT-TMT are at the excited state, the *t* indexes are −2.83 Å and −3.03 Å, respectively, implying the electrons and holes cannot be separated effectively, ICT could not occur (Fig. 3a, Supplementary Table 19), and thus ECL signal is not observed under the same experimental conditions (Fig. 3b). The theoretical calculation results are in good agreement with our molecular design concept. By limiting the donor and acceptor units within the tightly packed periodic array of COFs, the vertically ordered heterojunction form a wide D-A interface, which has an important role in promoting charge separation and redox reactions on the electrode surface. Meanwhile, the π-electrons can be delocalized along the COFs’ plane and be excited more easily.

In many reported cases, the linkage dominates the photoluminescence of COF by the dissipation of excitation energy via electron transfer. For example, the COFs linked by imine and hydrazone often emit poorly, even if highly luminous vertices are used^41–44^. In this context, it is necessary to choose one beneficial linkage for preventing linkage-originated ECL quenching. Under the same experimental conditions, the two olefin-linked COFs (DAFB-DCTP, DAFB-TMT) reveal much stronger ECL signals than the imine-linked COF (DAFB-Pa), which is also true for the three COFs of the BTTA-COFs series (Supplementary Fig. 40). This huge difference in ECL strength might be attributed to the different COFs linkages. Compared with the imine-linked COFs (DAFB-Pa, BTTA-Pa), the smaller semicircular domains of electrochemical impedance spectra for the olefin-linked COFs (DAFB-DCTP, DAFB-TMT, BTTA-DCTP, BTTA-TMT) indicate much more efficient interfacial charge transfer in the highly planar olefin-linked COFs skeletons (Supplementary Fig. 41). The utilization of -C═C- bonds as the linkage avoided the formation of distorted conformations due to the steric hindrance in the main chain when two aromatic units are directly coupled by the C–C single bonds, which is beneficial to form π-conjugate structure in the plane with a better π-communication. Similarly, the two corresponding model compounds MC-1, MC-2 show feeble ECL signals (Supplementary Fig. 42), which proves that the extended π-conjugated COFs are essential for achieving high ECL signals.

Based on the topological design strategy of COFs with π-communication that is provided by the olefin linkages and electrons dynamic coupling in D-A building blocks, the ECL of non-ECL molecules can be realized successfully in an aqueous medium.

### ECL-enhancement methodology of COFs

The tunable building blocks and channels in COFs are the remarkable properties that endow their enormous potential in further enhancing ECL signals. We then tested two strategies to improve the ECL performance in terms of methodology, including increasing the chain length and enhancing the degree of conjugation, aiming to inspire researchers to develop more efficient ECL luminophores suitable for practical applications.

On account of their pre-designed capacities, the porous structure of COFs can be fine-tuned by changing the composition of aromatic hydrocarbons. Albeit there is only one more para-phenyl unit in each spacer, the added twisted biphenyl in the skeleton will lead to the increase of interlayer separation, which is conducive to overcome the aggregation-induced quenching of organic molecules^34^. Researchers have also proved that the longer π-extension main chain is beneficial to improve the semiconductor’s performance^45,46^. However, their influences on ECL properties have not been systematically studied.

**Fig. 4:**
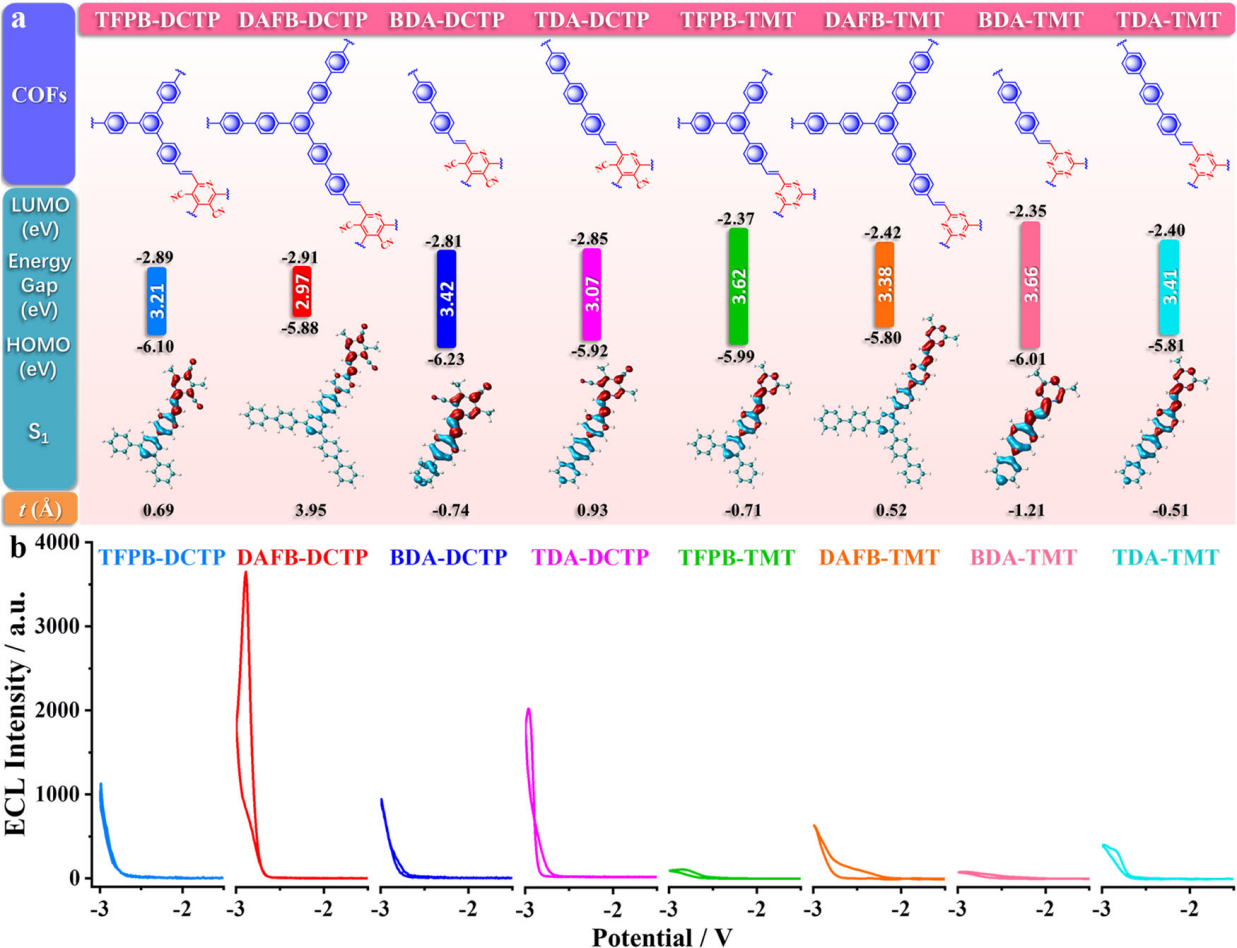
Influence of chain length of COFs on ECL performance. **a** DFT calculations to the building blocks of COFs. The values of HOMOs, LUMOs, and calculated energy gaps and analysis for the distribution of the hole (blue) and electron (red) for S_1_ are indicated. The *t* index is measured to evaluate the separation degree of hole and electron. **b** Comparison of ECL intensities of COFs in 0.1 M PBS with 0.1 M KCl between 0 V and −3.0 V. PMT: 800 V, pH 7.5, potential scan rate: 100 mV s^−1^.

DAFB is regarded as a molecule that adds a para-phenyl group in every direction of triangular aldehyde monomer TFPB. As shown in Fig. 4a and Supplementary Fig. 43, compared with TFPB-DCTP, the HOMO energy level of DAFB-DCTP is increased by 0.22 eV, and the HOMO-LUMO bandgap is decreased by 0.24 eV. When in the S_1_ excited state, the electron and hole are more efficiently separated (Supplementary Table 20). The UV absorption peak of DAFB-DCTP is broadened and the main absorption band shows significantly red-shifted (Supplementary Fig. 44). The optical bandgaps of DAFB-DCTP and TFPB-DCTP measured by the Kubelka–Munk function are 1.96 eV and 2.21 eV, respectively (Supplementary Fig. 44). Accordingly, DAFB-DCTP emits 3.5 times stronger ECL than TFPB-DCTP (Fig. 4b). Among the COFs composed of another C_3V_ receptor, TMT, the ECL intensity of DAFB-TMT is 4.6 times as large as that of TFPB-TMT (Fig. 4b). Among the COFs synthesized by DCTP/TMT and C_2_-symmetric dialdehydes (BDA-DCTP, TDA-DCTP, BDA-TMT, TDA-TMT), the ECL strength is also significantly enhanced by 2.1 (BDA-DCTP/TDA-DCTP) and 5.2 times (BDA-TMT/TDA-TMT), respectively, when the linear monomer BDA is extended into longer TDA (Fig. 4b, Supplementary Fig. 45). The universality of this strategy has been verified again in increasing ECL signals.

On the basis of our results, the distorted conformation of biphenyl and the simultaneous growth of the π-extension skeleton are two paramount factors to obtain enhanced ECL of COFs. We find that the HOMOs and LUMOs of COFs can be subtly regulated by simply changing the composition of polyaromatic hydrocarbons in the COFs’ skeleton, which further affect the ECL performance of the COFs. Thus, it is hopeful to single out favored ECL phosphors with different emission properties (emission wavelength, luminous intensity).

The ECL generated by conjugated organic molecules essentially originates from the charge recombination of the electron excitation systems that emit light upon relaxation^47^. Therefore, the migration of holes and electrons through the skeletons is crucial in luminescent molecules^44^. The benzene rings in building blocks connected by C–C single bond would rotate freely, which partially undermines the charge dislocation in COFs. Alkyne bonds have been proved to be fine connectors of photoactive molecular conductors owing to their highly conjugated structures and inherent abilities to modulate electronic structures and suppressing charge recombinations^48–50^, although the influence of alkynes on ECL properties of alkynes is rarely reported. Thus, we studied the ECL performance of acetylene-induced D-A COFs.

**Fig. 5:**
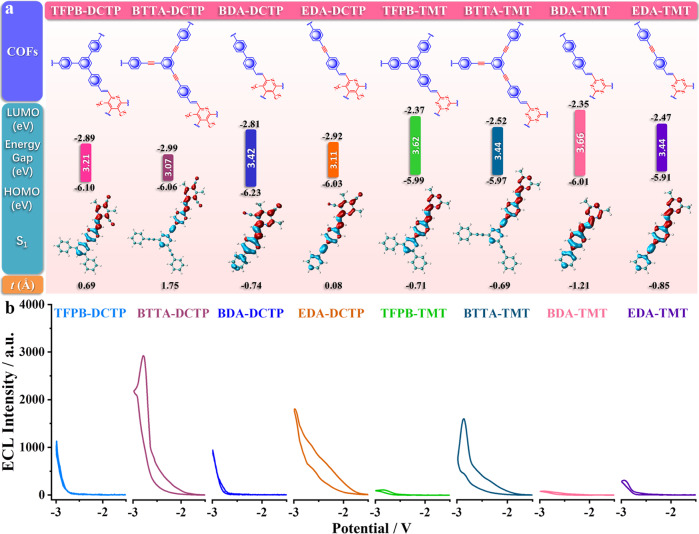
Influence of COFs’ conjugation degree on ECL performance. **a** DFT calculations to the building blocks of COFs. The values of HOMOs, LUMOs, and calculated energy gaps and analysis for the distribution of the hole (blue) and electron (red) for S_1_ are indicated. The *t* index is measured to evaluate the separation degree of hole and electron. **b** Comparison of ECL intensities of COFs in 0.1 M PBS with 0.1 M KCl between 0 V and −3.0 V, pH 7.5, PMT: 800 V, potential scan rate: 100 mV s^−1^.

Using the improved reaction conditions, a series of COFs were synthesized with BTTA/EDA as donors and DCTP/TMT as receptors. The calculated HOMO-LUMO distribution profiles show that the acetylene moieties have a significant contribution to the decreased LUMO levels by ~0.1 eV (Fig. 5a, Supplementary Fig. 46). Compared with TFPB and BDA formed by C–C, the introduction of alkynyl BTTA and EDA can enhance the coplanar conformation and conjugation degree of the resulted COFs (Fig. 5a). As revealed by the UV/Vis DRS analysis, BTTA-COFs reveal wider absorption ranges of ca. 618~678 nm and red-shifted absorption edges by ca. 58~93 nm as compared to the TFPB-COFs, since highly plane π-conjugated structures of BTTA-COFs greatly enhance π-electron delocalization in the two-dimensional frameworks. The optical bandgaps of BTTA-DCTP and TFPB-DCTP calculated from the Kubelka–Munk-transformed function is 2.05 and 2.21 eV, respectively, suggesting that the BTTA-COFs possess better semiconductor properties (Supplementary Fig. 47). The decrease of bandgaps and increase of t indexes can synergistically enhance the ECL signals (Supplementary Table 21). With equimolar masses, the ECL signal of BTTA-DCTP (2935 a.u.) is 2.7 times as large as that of TFPB-DCTP (1107 a.u.), and the ECL signal of EDA-DCTP (1809 a.u.) is 1.9 times as large as that of BDA-DCTP (946 a.u.) (Fig. 5b). Similar trends are also observed in BTTA-TMT (1607 a.u.)/TFPB-TMT (109 a.u.) and EDA-TMT (309 a.u.)/BDA-TMT (81 a.u.) (Fig. 5b, Supplementary Fig. 48). In addition, with the capacity of adsorbing O_2_, alkynyl shortens the distance between dissolved oxygen and COFs, which is favorable to promote the oxidation reaction on the electrode surface^51,52^.

Our results prove that introducing acetylene is a practical approach to enhance the ECL performance of the D-A COFs, based on the roles of acetylene in regulating the electronic structures and promoting the charge separation in the conjugated structure, as well as the special ability of adsorbing O_2_. Hence, the acetylene functionalized COFs show significant improvement in ECL strength.

### Electrochemical and ECL processes from COFs

To clarify the mechanism of electrochemistry and ECL of the COFs, DAFB-DCTP was selected as a representative of ECL-COFs in this experiment, and a series of control experiments were carried out. Considering the advantages of solid-state ECL in reducing the non-radiative relaxation and enhancing the electrocatalytic effect^3^, the prepared 10 μL 1 mM DAFB-DCTP/DMF solution was covered on the surface of a cleaned glassy carbon electrode (GCE) and dried at room temperature to complete the assembly of the COF on the electrode surface.

After cathodic and anodic potential optimization, the strongest ECL signal was obtained by cyclic voltammetry (CV) within a potential window from −3 V to 0 V (vs. Ag/Ag^+^), and the electrochemical and ECL behaviors of COFs were studied (Supplementary Fig. 49). In addition, the ECL signals in different solvents were further tested. Surprisingly, ECL can be generated in both the organic phases (DMF, CH_3_CN, THF, DMSO, and CH_2_Cl_2_, contained 0.1 M TBAPF_6_ as supporting electrolyte) and the aqueous solution (PBS with 0.1 M KCl) as shown in Supplementary Fig. 50. Better ECL performance is obtained in the aqueous medium. On the contrary, under the same test conditions, all the reported typical organic luminophores (including diphenylanthracene (DPA), perylene (Pe), 5,10,15,20-tetrakis(4-hydroxyphenyl)porphyrin (THPP), rubrene, boron dipyrromethene (BODIPY), and pyrene (Py) show a faint luminescence or luminescence quenching, which may be due to the poor solubility or stability of free radical ions and aggregation-induced quenching occurred in organic luminescence caused by water and oxygen (Supplementary Fig. 51). COFs’ superiorities may be attributed to the eliminated harmful π-π accumulation between COFs’ molecules in poor solvents, avoiding aggregation-induced quenching. In addition, the inhibited molecular motion would block the non-radiative decay channels, which is beneficial to the occurrence of ICTs from the donors to the acceptors^53^. Coupled with these advantages on flexibility and diversity, COFs are endowed with great potentials for application in life-related ECL systems.

**Fig. 6:**
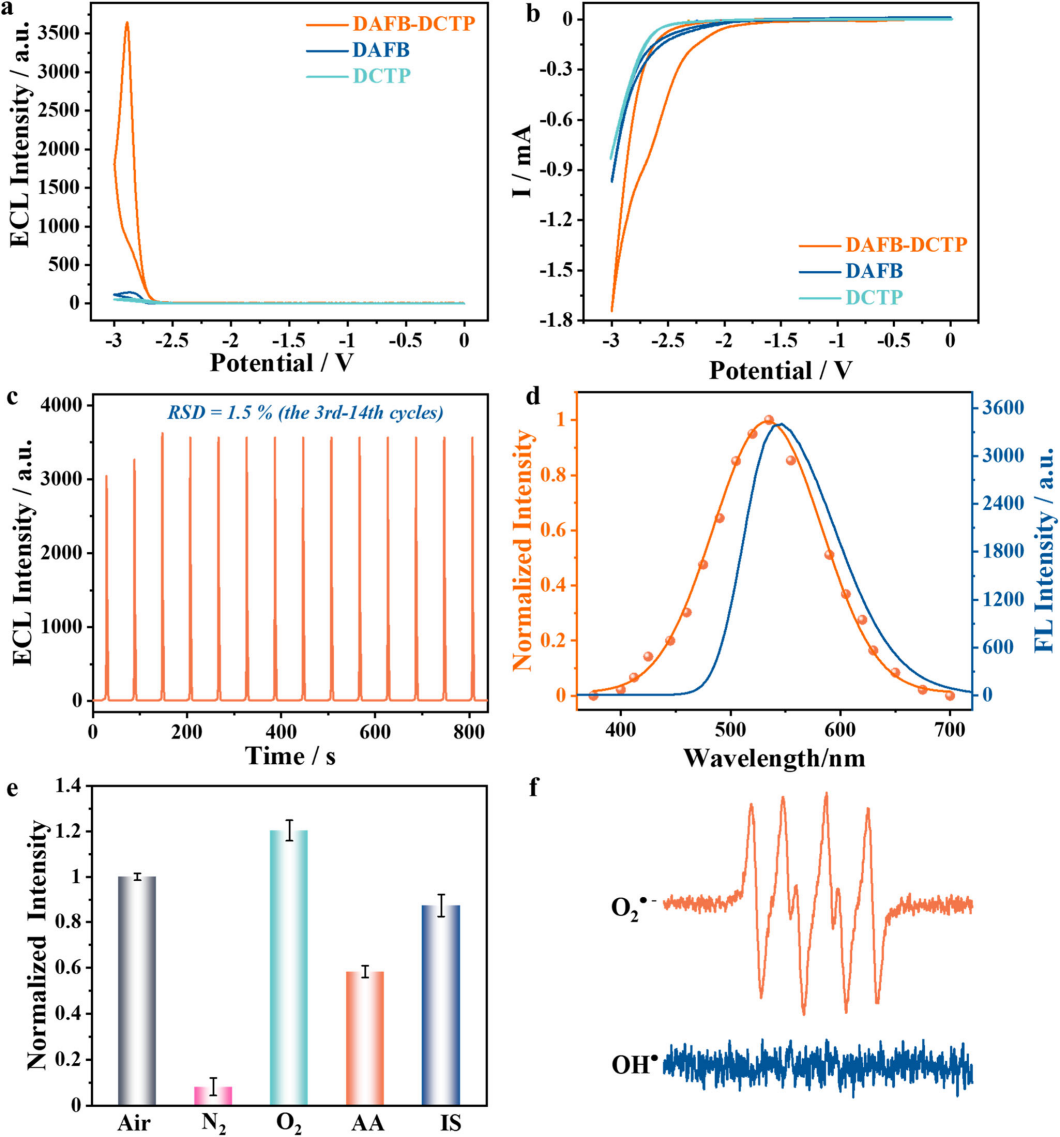
Exploration of electrochemical and ECL mechanism from COFs. **a** ECL spectra and **b** corresponding CVs of DAFB-DCTP (orange curve), DAFB (blue curve), and DCTP (green curve) modified GCE. **c** The ECL signals of DAFB-DCTP/GCE under a continuous potential scan. **d** ECL spectrum of DAFB-DCTP/GCE (orange curve) and FL spectrum of DAFB-DCTP in DMF (blue curve). **e** The normalized ECL intensity of DAFB-DCTP/GCE in the buffer solution containing air, N_2_-saturated, O_2_-saturated, 0.1 mM AA, or 0.1 mM IS. Error bars represent S.D. *n* = 3 independent experiments. **f** The EPR spectra of DAFB-DCTP. All the buffer solutions throughout the ECL/CV experiments were 0.1 M PBS with 0.1 M KCl between 0 V and −3.0 V, pH 7.5, PMT: 800 V, potential scan rate: 100 mV s^−1^.

Figure 6a, b show the ECL-voltage spectra and cyclic voltammograms (CVs) of DAFB-DCTP, respectively. An obvious cathodic ECL signal starts at −2.6 V, increases sharply, and reaches a maximum near −2.9 V (Fig. 6a, orange curve). Using Ru(bpy)_3_^2+^ as a benchmark, the Φ_ECL_ of DAFB-DCTP was determined to be 32.5%. It is much higher than the reported ECL luminophores (Supplementary Table 22) even in the aqueous medium (PBS) and without the aid of any exogenous co-reactants. As expected, almost no ECL signal is observed in the monomers DAFB (Fig. 6a, blue curve) and DCTP (Fig. 6a, green curve), the GCE (Supplementary Fig. 52), and any other molecules consisted COFs (Supplementary Figs. 53 and 54), which exclude the ECL from monomer and other undesirable side reactions. Matching with the appearance of ECL signal, a reduction wave of DAFB-DCTP appears at ~−2.6 V (Fig. 6b, orange curve), indicating that free radical anions are formed at the beginning of the electron injection process toward COFs. Thus, the electrochemical reduction process of COFs initiates the ECL emission. Moreover, when the COFs are cast on a GCE, the redox peak current increases significantly compared with the monomers (Fig. 6b, blue and green curves), demonstrating that the large specific surface area and high conductivity of the COFs provide a platform for fast charge transfer at the electrode interface.

During the continuous potential scans, the ECL emission shows progressively increase and reaches a relatively stable value (the relative standard deviation (RSD) of only 1.5%) after third scans (Fig. 6c). The increase in ECL signal within the first few scans may be owing to the accumulation of some oxidized species. The DAFB-DCTP structure during the process of the electrochemical cycle remains intact and the crystal is not destroyed, guaranteeing good stability, and reproducibility (Supplementary Fig. 55). These results elucidate that the COFs films formed on the electrode have high stability and their structures remain under continuous potential scans, which ensures fine repeatability.

As shown in Fig. 6d, the ECL emission peak of DAFB-DCTP (535 nm, orange curve) is very close to the maximum fluorescence emission wavelength (546 nm, blue curve). Considering the inner filter effect of the instruments and the coarse wavelength interval (15–25 nm) of the bandpass filters used in the ECL spectroscopic measurement, the luminescence of DAFB-DCTP can be classified as the bandgap mechanism^54^. The relative band position of DAFB-DCTP was estimated by Mott-Schottky (M-S) analysis to further investigate the electron structure. The positive slope of the M-S plots indicates that DAFB-DCTP is an n-type semiconductor (Supplementary Fig. 56), in which the electrons are the majority carriers^55–57^. Accordingly, the flat-band potential (E_FB_) of DAFB-DCTP can be fitted to be −1.16 V vs RHE from the x-intercept of the linear region of the M-S diagram. Considering that the conduction band minimum of n-type semiconductors is slightly negative than its flat-band potential (~0.2 eV)^55–57^ and combined with the optical bandgap measured by the Kubelka–Munk function on the basis of UV-vis spectrum, the valence band potential (E_VB_) and conduction band potential (E_CB_) were summarized in the inset of Supplementary Fig. 56.

To verify the active intermediates formed within the ECL process, the effects of different atmospheric conditions and radical scavengers on ECL signals were studied. As listed in Fig. 6e, the COF itself shows very weak luminescence (reduced by 91%) in N_2_-saturated PBS. The ECL is enhanced by 1.2 times in O_2_-saturated solution, suggesting that the dissolved oxygen plays a synergistic role. We then used 0.1 mM ascorbic acid (AA) and isopropanol (IS) as the scavengers for superoxide anion radical (O_2_^•−^)^58^ and hydroxyl radical (OH^•^)^4^ respectively, and found that AA significantly inhibits the ECL, while IS almost has no effect. These control experiments unravel that the ECL reaction proceeds via a radical pathway, which is also confirmed by electron paramagnetic resonance measurement showing the formation of a huge number of O_2_^•−^ within COFs in the ECL process, but no OH^•^ (Fig. 6f). The participant of dissolved oxygen in the ECL process is guaranteed by its free diffusion in the rich pores of COFs. On the basis of our results, we propose the ECL emission mechanism of D-A COFs through the following Eqs. (1) to (5):

During the cathodic potential scan from 0 V to −3.0 V, O_2_ is first electrochemically reduced to the oxidizing intermediate, O_2_^•−^, via Eq. (1). Then, the hole of O_2_^•−^ is injected into the HOMO orbitals concentrated in the COFs’ donor parts. The expanded charge transport network of COFs can promote hole transport and generate cationic free radicals Eq. (2). When the potential reaches enough negative values, electrons will be injected into the LUMO orbitals concentrated in the COFs receptors from the electrode to form anion free radicals Eq. (3). Two kinds of free radical ions of COFs collide with each other to the ICT state directly and return to the ground state via ECL emission, emitting a strong luminescence Eqs. (4)–(5).

Hereby, the ECL from fully conjugated D-A COFs, generating ECL based on the collision between the electron-donors and the acceptors show the much stronger ECL, slightly dissolved oxygen are borrowed to act as a co-reactant and no highly oxidizing co-reactants like H_2_O_2_, S_2_O_8_^2−^ or TPrA is needed. The research on the ECL mechanism of COF is still blank to date, as a supplement to the ECL basic research, it may provide a feasible method and reference mechanism for activating the ECL response of non-ECL molecules.

### Discussion on the COF structure−ECL performance relationship

Although COFs possess attractive designability, the inherent properties of dynamic covalent bonds generally lead to weak π-electron communication, which limits the electron transfer between components^32,33^. Only a handful of COFs has been reported the ECL phenomenon to date^2,8^. For them, even if the typical ECL groups (pyrene) were linked to form the main part of the COF framework, the observable ECL signal requires not only the highly oxidizing co-reactants (H_2_O_2_/S_2_O_8_^2−^), but the assistance of nano-enzymes Co_3_O_4_ or accelerator Bu_4_NPF_6_^2,8^. The tedious steps would greatly limit the versatility of practical applications. As for COFs per se, no distinct ECL signal was obtained. Although the design of these COFs is still limited to the improvement of the existing luminophores, these works did prove that COFs are the most anticipated luminescent material.

This contribution focuses on the construction of highly efficient ECL-COFs. It has been identified that the extended reticular D-A crystal framework decreases the bandgap and increases the ECL strength by shortening the electron migration distance from VB to CB. In this case, the appropriate structure design for the ECL-COFs permits the electrons and holes to be effectively separated, transferred, excited, and collided, making it a promising ECL luminophore family. The most important findings from the ECL intensity assessment reveal the structure−ECL performance relationship are summarized as follows: (1) In contrast to the olefin-linked COFs, other imine-linked COFs (BTTA-Pa, DAFB-Pa), only showed dim signals, which highlights the contribution from olefin linkages. (2) The two model compounds (MC-1 and MC-2) showed negligible ECL intensity, which verifies the importance of the extended π-conjugated framework. (3) Compared with fully conjugated D-A COFs, another olefin-linked COFs (TFPT-DCTP and TFPT-TMT) with no D-A relationship, showed no ECL signal, demonstrating that ECL indeed originates from the ICTs between the D-A building blocks. (4) As two promising enhancing approaches, increasing the chain length of the donor subunits can get narrower optical bandgaps and stronger ECL signals due to the increased *t* index and interlayer separation, and introducing the alkynyl to COF skeleton can obtain a stronger ECL signal by enhancing the conjugation degree and shortening the distance to dissolved oxygen. (5) The ECL intensity of DAFB-DCTP is superior among all the organic phosphors reported to date (Supplementary Fig. 46), which provides overwhelming evidence for the unique advantages of D-A olefin-linked COFs.

Last but not least, the generality of the standpoints raised in this research is emphasized by changing the receptor and symmetry. The previous work relied on existing ECL research and only considered how to weave the phosphors into the COF skeleton. In contrast, this reference is absent for our general approach to ECL-COFs here: model compounds and monomers hardly showed ECL performance, while there is a significant difference when D-A building blocks extending in the olefin-linked crystal framework. This approach is more controllable and more abundant than AIE because the crystal structure can be designed precisely with a stable stroke. Accordingly, this contribution will provide insight for the exploration of organic emitters from small molecules or oligomers to crystalline extended frameworks.

## Discussion

A general topology design strategy of COFs was reported as a family of ECL luminophores. We achieved the ICT to initiate ECL via restriction of the donors and acceptors to the COFs’ tight electron configurations and construction of the high-speed charge transport network through the olefin linkages. Thus, the assembled COFs exhibit the cathodic ECL in an aqueous medium using dissolved oxygen as an amplifier without adding external toxic co-reactants. Our results show that increasing the chain length and using the alkyne bonds can effectively decline the non-radiative damping of the excited states further enhance the molecular ECL strength, which may inspire researchers to customize the COFs suitable for designed applications. With the help of density functional theory (DFT) calculations and control experiments, a mechanism of COFs structure-related ECL has been suggested that the ECL of COFs is attributed to the bandgap mechanism through the free radical pathway. Providing a powerful platform for tailor-made sensor analysis, the COF-ECL system here is very suitable for dealing with various types of environmental pollution, drug analysis, and life monitoring. Furthermore, the present concept could be promising to design more efficient COFs based luminophores by combining machine learning to run high-throughput virtual screening, hopefully opening up broader applications of COF materials and ECL technology.

## Methods

### Materials

Piperidine, DBU, trifluoroacetic acid and acetonitrile (ACN) were purchased from Energy Chemical Technology (Shanghai) Co., Ltd. 2,4,6-Trimethyl-1,3,5-triazine (TMT), 2,4,6-trimethylpyridine-3,5-dicarbonitrile (DCTP), 2,4,6-tris(4-formylphenyl)-1,3,5-triazine (TFPT), 1,3,5-tris(4-formylphenyl)benzene (TFPB), 4-[4-[3,5-bis[4-(4-formylphenyl)phenyl]phenyl]phenyl]benzoic acid (DAFB), 4,4,4-(benzene-1,3,5-triyltris(ethyne-2,1-diyl))tribenzaldehyde (BTTA), 4,4’-biphenyldicarboxaldehyde (BDA), 4,4”-*p*-terphenyldicarboxaldehyde (TDA), 4-[2-(4-formylphenyl)ethynyl]benzaldehyde (EDA), tris(4-formylphenyl)amine (TPA), and *p*-phenylenediamine (Pa) were purchased from Jilin Chinese Academy of Sciences-Yanshen Technology Co., Ltd. Tetrahydrofuran (THF), 1,4-dioxane, mesitylene, benzaldehyde, NaOH, KOH, EtONa, N,N-dimethylformamide (DMF), 2,2,6,6-tetramethylpiperidine (TEMP), ethanol, methanol, CH_2_Cl_2_, acetone, and *n*-BuOH were purchased from Sinopharm Chemical Reagent Co., Ltd. Ultrapure water was prepared from the Millipore system (18.25 MΩ-cm). All the electrochemical measurement used phosphate buffered (PBS, pH = 7.5) containing mixture solutions of 0.1 M KCl, 0.1 M K_2_HPO_4_, and 0.1 M KH_2_PO_4_ as the electrolyte.

### Synthesis of DCTP-COFs

DCTP (17.1 mg, 0.1 mmol), anhydrous DMF (4.0 mL), and piperidine (51.1 mg, 0.6 mmol) were added separately with 8 different aldehydes in a 20 mL Pyrex tube. The amount of aldehydes were taken as DAFB (61.8 mg, 0.1 mmol), BTTA (46.2 mg, 0.1 mmol), TDA (42.9 mg, 0.15 mmol), EDA (35.1 mg, 0.15 mmol), BDA (31.5 mg, 0.15 mmol), TPA (32.9 mg, 0.1 mmol), TFPB (39.0 mg, 0.1 mmol), and TFPT (39.3 mg, 0.1 mmol), respectively. The mixture was degassed by three freeze–pump–thaw cycles, sealed under vacuum, stirred for 10 min, and heated in a 180 °C oil bath for 3 days. The reaction mixture was cooled to room temperature, and a yellow precipitate was collected by centrifugation, washed several times with methanol, CH_2_Cl_2_, and THF, respectively. It was then Soxhlet extracted in CH_2_Cl_2_ and THF for 24 h and dried under vacuum at 60 °C for 12 h to afford DAFB-DCTP (yield, 78%), BTTA-DCTP (yield, 80%), TDA-DCTP (yield, 77%), EDA-DCTP (yield, 75%), BDA-DCTP (yield, 83%), TPA-DCTP (yield, 76%), TFPB-DCTP (yield, 81%), and TFPT-DCTP (yield, 79%), respectively.

### Synthesis of TMT-COFs

TMT (12.3 mg, 0.1 mmol), 0.9 mL mesitylene, 0.9 mL 1,4-dioxane, 0.4 mL trifluoroacetic acid, and 0.05 mL ACN were added separately with eight different aldehydes in a 20 mL Pyrex tube. The amount of aldehydes were taken as DAFB (61.8 mg, 0.1 mmol), BTTA (46.2 mg, 0.1 mmol), TDA (42.9 mg, 0.15 mmol), EDA (35.1 mg, 0.15 mmol), BDA (31.5 mg, 0.15 mmol), TPA (32.9 mg, 0.1 mmol), TFPB (39.0 mg, 0.1 mmol), and TFPT (39.3 mg, 0.1 mmol), respectively. The mixture was degassed by three freeze–pump–thaw cycles, sealed under vacuum, stirred for 10 min, and heated in a 150 °C oil bath for 3 days. The reaction mixture was cooled to room temperature, and the solid was collected by centrifugation, washed with acetone and methanol, neutralized by 0.1 mol L^−1^ NH_4_OH solution in aqueous methanol (50 wt%), and then washed with methanol in a Soxhlet extractor for 12 h and dried under vacuum at 60 °C for 12 h to afford DAFB-TMT (yield, 64%), BTTA-TMT (yield, 67%), TDA-TMT (yield, 70%), EDA-TMT (yield, 65%), BDA-TMT (yield, 72%), TPA-TMT (yield, 65%), TFPB-TMT (yield, 71%), and TFPT-TMT (yield, 69%), respectively.

### Modifying electrodes

The GCE scrubbed by soaked filter paper was polished with 1.0 μm, 0.3 μm, and 0.05 μm alumina powder slurry on chamois leather until showing a mirror surface. Subsequently, the electrodes were ultrasonic in 1:1 HNO_3_:H_2_O, anhydrous ethanol, and ultrapure water at 40% power for 1 min, respectively, and dried in the nitrogen flow. The prepared COFs/DMF solution (1 mM, 10 μL) was covered on the surface of GCE and dried at room temperature for characterization and further use.

### Electrochemical and ECL measurements

The ECL measurements were carried out on an MPI-E multifunctional electrochemical analytical system (Xi’an Remex Analytical Instrument Co. Ltd., Xi’an, China) with the voltage of the photomultiplier tube at 800 V and the scan rate at 100 mV s^−1^, as well as the potential scan was from 0 to −3.0 V in the detection process. The cyclic voltammograms were measured at a sweep rate at 100 mV s^−1^ and with voltage sweeps between 0 and −3.0 V. The ECL and CV were conducted at room temperature in a classical three-electrode configuration consisting of a modified carbon glassy electrode working electrode, a platinum counter electrode, and an Ag/AgCl reference electrode.

### ECL efficiency calculation

ECL efficiency (Φ_ECL_) of the COFs was obtained by the following equation, and [Ru(bpy)_3_]^2+^ was used as a reference^3,59^.

$${\Phi }_{{{{\rm{ECL}}}}}={\Phi }_{{{{\rm{ECL}}}}}^{{{{\rm{\circ }}}}}\left(\frac{I{Q}_{f}^{{{{\rm{\circ }}}}}}{{I}^{{{{\rm{\circ }}}}}{Q}_{f}}\right)$$

where Φ^°^ is the ECL efficiency of the [Ru(bpy)_3_]^2+^ system, I and I^°^ represent the ECL intensities of the COFs and [Ru(bpy)_3_]^2+^, respectively. Q_f_ and Q^°^_f_ correspond to the respective faradaic charge passed for the COFs and [Ru(bpy)_3_]^2+^. The I^°^ and Q^°^_f_ for 1 mM [Ru(bpy)_3_]^2+^ was measured in ACN containing 0.1 M TBAP at 23 °C. While *I* and *Q*_*f*_ for COFs, the procedures were the same as the ECL experimental described above.

### Calculation conditions

The DFT calculations were carried out in the programs of Gaussian09 and calculated the functional with B3LYP. The basis set was calculated by 6–311 G (d). In order to calculate accurately, the DFT-D3 dispersion correction was used, and the excited-state calculation was calculated by TD-B3LYP method. The properties of excited states were analyzed by Multiwfn.

## Supplementary information

Supplementary Information

## Data Availability

All data generated in this study are provided in the Article and its Supplementary Information. The other data that support the findings of this study are available from the corresponding author upon request.
