# Author Correction: A general design approach toward covalent organic frameworks for highly efficient electrochemiluminescence

**DOI:** 10.1038/s41467-021-25868-x

**Published:** 2021-09-16

**Authors:** Ya-Jie Li, Wei-Rong Cui, Qiao-Qiao Jiang, Qiong Wu, Ru-Ping Liang, Qiu-Xia Luo, Jian-Ding Qiu

**Affiliations:** 1 College of Chemistry, Nanchang University, 330031 Nanchang, China; 2 College of Materials and Chemical Engineering, Pingxiang University, 337055 Pingxiang, China

**Keywords:** Polymers, Electronic properties and materials, Organic molecules in materials science

Correction to: *Nature Communications* 10.1038/s41467-021-25013-8, published online 05 August 2021.

The original version of the Supplementary Information of this Article contained an error in Supplementary Figs. 3–10, in which the second diffraction peak of the powder XRD diffractogram was wrongly assigned to (100) instead of (110).

Furthermore, the original version of the Supplementary Information of this Article contained an error in the figure captions of Supplementary Fig. 1 and Supplementary Fig. 2, which incorrectly omitted the following information:

Caption of Supplementary Figure 1: ‘TDA-DCTP, BDA-DCTP, and TFPB-DCTP were synthesized based on a reported method^3^’.

Caption of Supplementary Figure 2: ‘TFPT-TMT was synthesized based on a reported method^4^.’

The HTML has been updated to include a corrected version of the Supplementary Information. The correct version of the Supplementary Information can be found associated with this Correction.

## Supplementary information

Supplementary information
