# Supplementary material for: A general design approach toward covalent organic frameworks for highly efficient electrochemiluminescence
Source: Nat Commun. 2021 Aug 5;12:4735. doi: 10.1038/s41467-021-25013-8 (PMC8342611; doi:10.1038/s41467-021-25013-8)
Supplement: Supplementary file 1 — Supplementary Information [file 41467_2021_25013_MOESM1_ESM.pdf]

Supplementary Information

A general design approach toward covalent organic frameworks for highly efficient electrochemiluminescence

Ya-Jie Li,[1,3] Wei-Rong Cui,[1,3] Qiao-Qiao Jiang,[1] Qiong Wu,[1] Ru-Ping Liang,[1] Qiu-Xia Luo,[1] and Jian-Ding Qiu[1,2]*

[1]College of Chemistry, Nanchang University, Nanchang 330031, China

[2]College of Materials and Chemical Engineering, Pingxiang University, Pingxiang 337055, China

[3]These authors contributed equally: Ya-Jie Li, Wei-Rong Cui.

*Corresponding authors. Tel/Fax: +86-791-83969518. E-mail: jdqiu@ncu.edu.cn.

1. Instruments. Fourier-transform infrared (FT-IR) spectra were recorded on a Nicolet Impact 410 FT-IR spectrometer. Powder X-ray diffraction (PXRD) data of the nanomaterials were collected on a Bruker AXS D8 Advance A25 Powder X-ray diffractometer (40 kV, 40 mA) using Cu Kα (λ=1.5406 Å) radiation. ^{1}H NMR and ^{13}C NMR spectra were checked by Varian instruments (400 MHz). ^{13}C cross-polarization/magic angle spinning solid-state nuclear magnetic resonance (CP/MAS ssNMR) experiments were performed on a Bruker AVANCE III 400 WB spectrometer operating at 100.62 MHz for ^{13}C using a double resonance 4 mm MAS NMR probe and a sample spinning rate of 5 kHz. The nitrogen adsorption and desorption isotherms were measured at 77 K using a Micromeritics ASAP 2020M system. The samples were outgassed at 120 ℃ for 12 h before the measurements. Surface areas were calculated from the adsorption data using Brunauer-Emmett-Teller (BET) methods. The pore-size-distribution curves were obtained via the non-local density functional theory (NLDFT) method. The thermal properties of the nanomaterials were evaluated using a STA PT1600 Linseis thermogravimetric analysis (TGA) instrument over the temperature range of 30 to 800 ℃ under nitrogen atmosphere with a heating rate of 10 ℃/min. UV-vis diffuse reflectance spectra (UV-vis DRS) were recorded at room temperature on a HITACHI U-4100 Spectrophotometer.

2. Synthesis of DAFB-Pa.

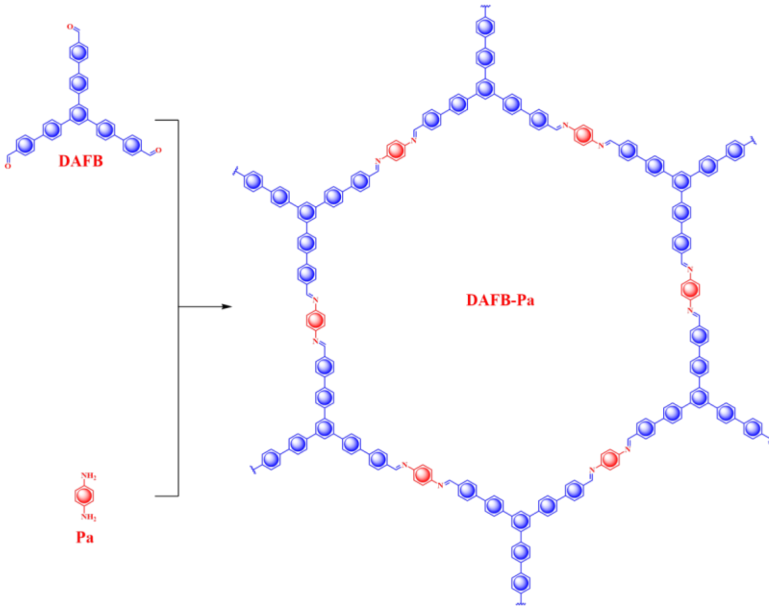

To a 20 mL Pyrex tube, DAFB (66.1 mg, 0.1 mmol) and Pa (16.2 mg, 0.15 mmol), 1,4-dioxane (1.0 mL), mesitylene (1.0 mL) and 6 M aqueous acetic acid (0.2 mL) were added. The tube was frozen at 77 K using a liquid N_2 bath. After three freeze-pump-thaw cycle the tube was evacuated and flame-sealed. Upon warming to room temperature, the tube was placed in an oven at 120 ℃ and left undisturbed for 3 days. The reaction mixture was cooled to room temperature, and the solid was isolated by filtration and washed several times with anhydrous THF, dried at 60 ℃ under vacuum for 12 h to yield DAFB-Pa (yield, 76%).

3. Synthesis of BTTA-Pa.

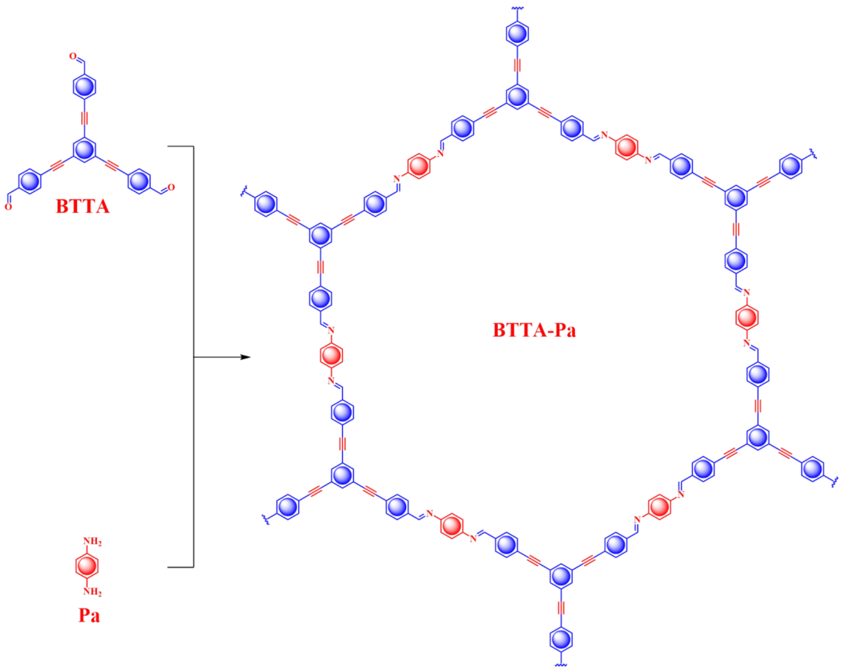

To a 20 mL Pyrex tube, BTTA (48.7 mg, 0.1 mmol) and Pa (16.2 mg, 0.15 mmol), 1,4-dioxane (1.0 mL), mesitylene (1.0 mL) and 6 M aqueous acetic acid (0.2 mL) were added. The tube was frozen at 77 K using a liquid N_2 bath. After three freeze-pump-thaw cycle the tube was evacuated and flame-sealed. Upon warming to room temperature, the tube was placed in an oven at 120 ℃ and left undisturbed for 3 days. The reaction mixture was cooled to room temperature, and the solid was isolated by filtration and washed several times with anhydrous THF, dried at 60 ℃ under vacuum for 12 h to yield BTTA-Pa (yield, 74%).

4. Synthesis of model compound 1 (MC-1).

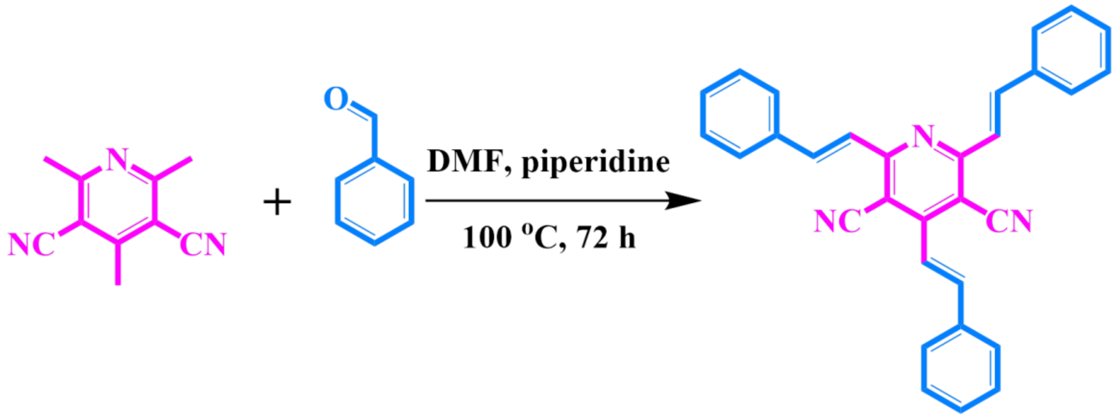

DCTP **Benzaldehyde** **MC-1**

MC-1 was synthesized by the reported method.[1] An oven-dried long-neck Schlenk flask was equipped with a magnetic stir bar and charged with 3,5dicyano-2,4,6-trimethylpyridine (DCTP) (85.6 mg, 0.5 mmol), benzaldehyde (318.5 mg, 3.0 mmol), piperidine (255.5 mg, 3.0 mmol) and 10 mL anhydrous DMF under the protection of nitrogen. Then the mixture was heated to 100 ℃ and stirred for 72 h under nitrogen atmosphere. After cooling down to room temperature, the solution was poured into water and extracted with dichloromethane, dried over $MgSO_4$, and concentrated to obtain a brown solid. The crude product was purified by column chromatography over a silica gel column using petroleum ether–dichloromethane (v/v, 1/1) as the eluent to afford MC-1 as yellow solids. Yield: 53%. ^{1}H NMR (400 MHz, Chloroform-*d*) δ 8.28 (d, J = 15.3 Hz, 2H), 7.96 (d, J = 16.5 Hz, 1H), 7.78–7.72 (m, 4H), 7.70–7.59 (m, 4H), 7.46 (m, 7.49-7.39, 9H), 7.39 (d, J = 0.8 Hz, 1H). ^{13}C NMR (101 MHz, Chloroform-*d*) δ 159.28, 142.41, 141.51, 135.24, 130.55, 130.36, 129.10, 129.06, 128.43, 128.06, 122.44, 120.33, 115.83, 102.83.

5. Synthesis of model compound 2 (MC-2).

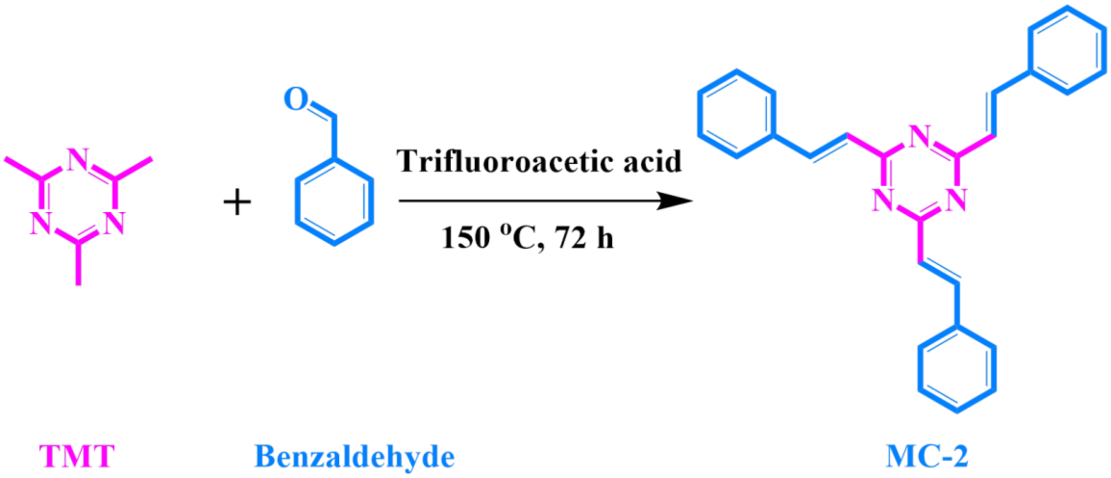

MC-2 was synthesized by the reported method.[2] A Pyrex tube was charged with 2,4,6-trimethyl-1,3,5-triazine (TMT, 6.0 mg, 0.049 mmol), benzaldehyde (15.9 mg, 0.075 mmol), 0.45 mL mesitylene, 0.45 mL 1,4-dioxane, 0.20 mL trifluoroacetic acid, and 0.025 mL acetonitrile. The tube was flash frozen at 77 K (liquid N_2 bath), evacuated to an internal pressure of 500 mTorr, and flame sealed. Upon sealing, the length of the tube was reduced to 7−8 cm. The reaction was carried out at 150 °C for 72 h to give a red solution. The solution was further neutralized with saturated aqueous $NaHCO_3$ solution and evaporated under reduced pressure to remove volatile compounds. The resulting solid was extracted with dichloromethane (DCM, 50 mL), washed with water (50 mL) and brine (50 mL), and dried over Na_2SO_4. The solvent was evaporated, and the product was purified by flash column chromatography (hexanes/DCM = 1:4) to yield an off white solid. yield: 62%. ^{1}H NMR (400 MHz, Chloroform-*d*) δ 8.36 (d, J = 15.9 Hz, 3H), 7.81–7.71 (m, 6H), 7.55–7.42 (m, 9H), 7.29 (d, J = 36.4 Hz, 2H). ^{13}C NMR (101 MHz, Chloroform-*d*) δ 171.33, 141.70, 135.53, 129.91, 128.95, 128.21, 126.34.

6. Supplementary Figures and Tables.

The crystal structures of COFs were elucidated by comparing the diffraction results with the structural simulations. The powder X-ray diffraction (PXRD) patterns, of all fully conjugated COFs showed highly intense reflection peaks at (100) facet (Supplementary Figs. 1-10), indicating their high crystallinity of these as-synthesized COFs, of which the PXRD diffraction patterns matched well with the eclipsed AA-stacking models of the simulated structures (Supplementary Tables 1-16). Their chemical structure and composition were further confirmed from the Fourier transform infrared (FT-IR) spectra (Supplementary Figs. 11-14), and ^{13}C cross polarization/magic angle spinning solid-state nuclear magnetic resonance (^{13}C CP/MAS ssNMR) spectroscopy (Supplementary Figs. 15-18). To evaluate the porosities of these as-synthesized COFs, N_2 adsorption-desorption isotherms were determined at 77 K (Supplementary Figs. S19-S26). All of these as-synthesized COFs showed a high Brunauer–Emmett–Teller (BET) surface area (> 492 m^2 g^{-1}), and their pore size distribution was in good agreement with their AA-stacking model, further confirming their high crystallinity. Moreover, COFs' good thermal stabilities were implied by the thermogravimetric analysis (TGA) curves (Supplementary Figs. 27-28). The morphological properties were studied by the scanning electron microscope (SEM), which showed that all the COFs possess an analogous fibrillar crystal morphology (Supplementary Figs. 31-35). The characterization results confirm the successful construction of high crystallinity COFs involved in this work.

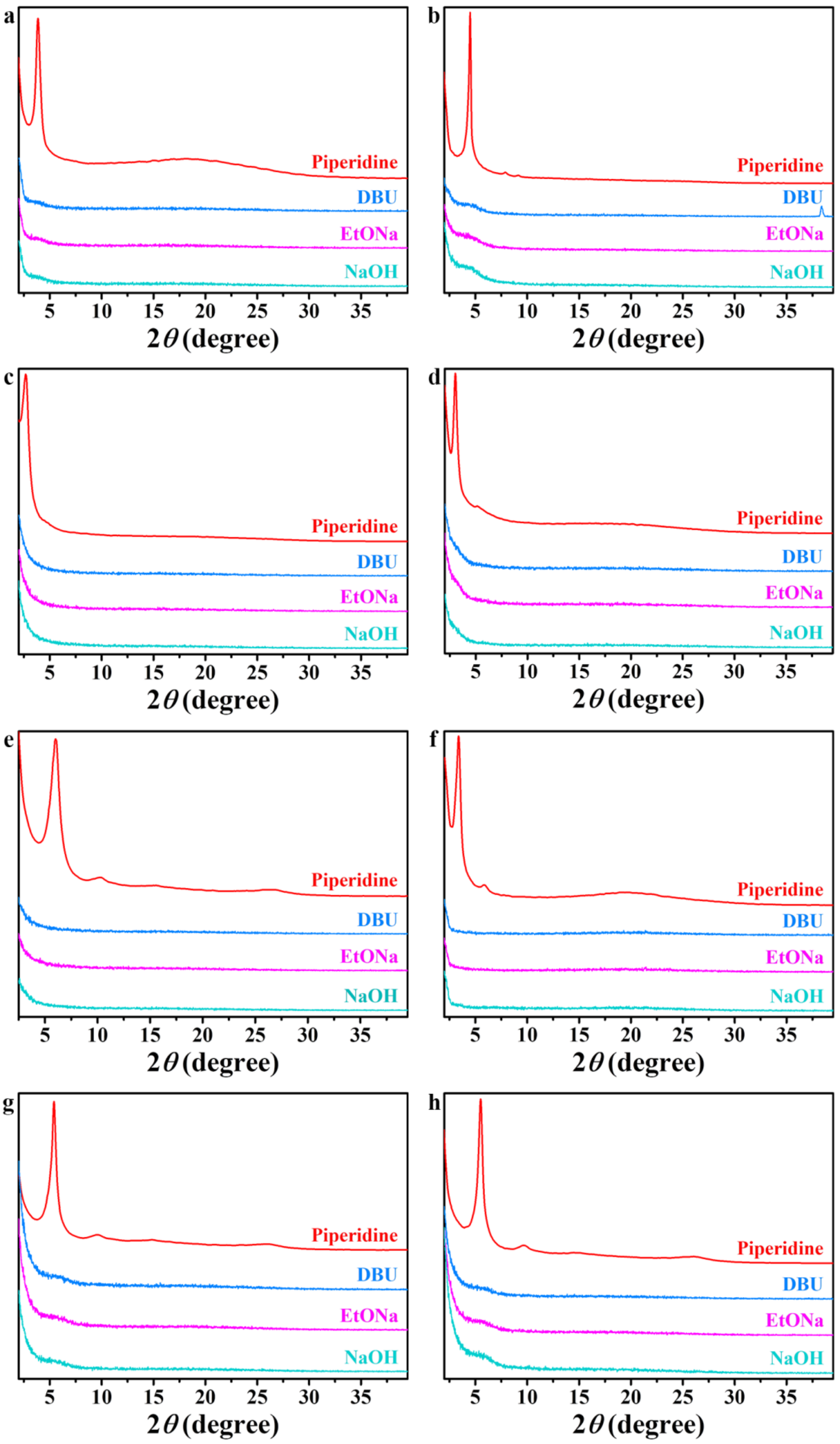

Supplementary Fig. 1 | PXRD patterns of attempts to synthesize DAFB-DCTP (a), BTTA-DCTP (b), TDA-DCTP (c), EDA-DCTP (d), TPA-DCTP (e), BDA-DCTP (f), TFPB-DCTP (g), and TFPT-DCTP (h) under different catalyst conditions.

TDA-DCTP, BDA-DCTP, and TFPB-DCTP were synthesized based on the reported method.[3]

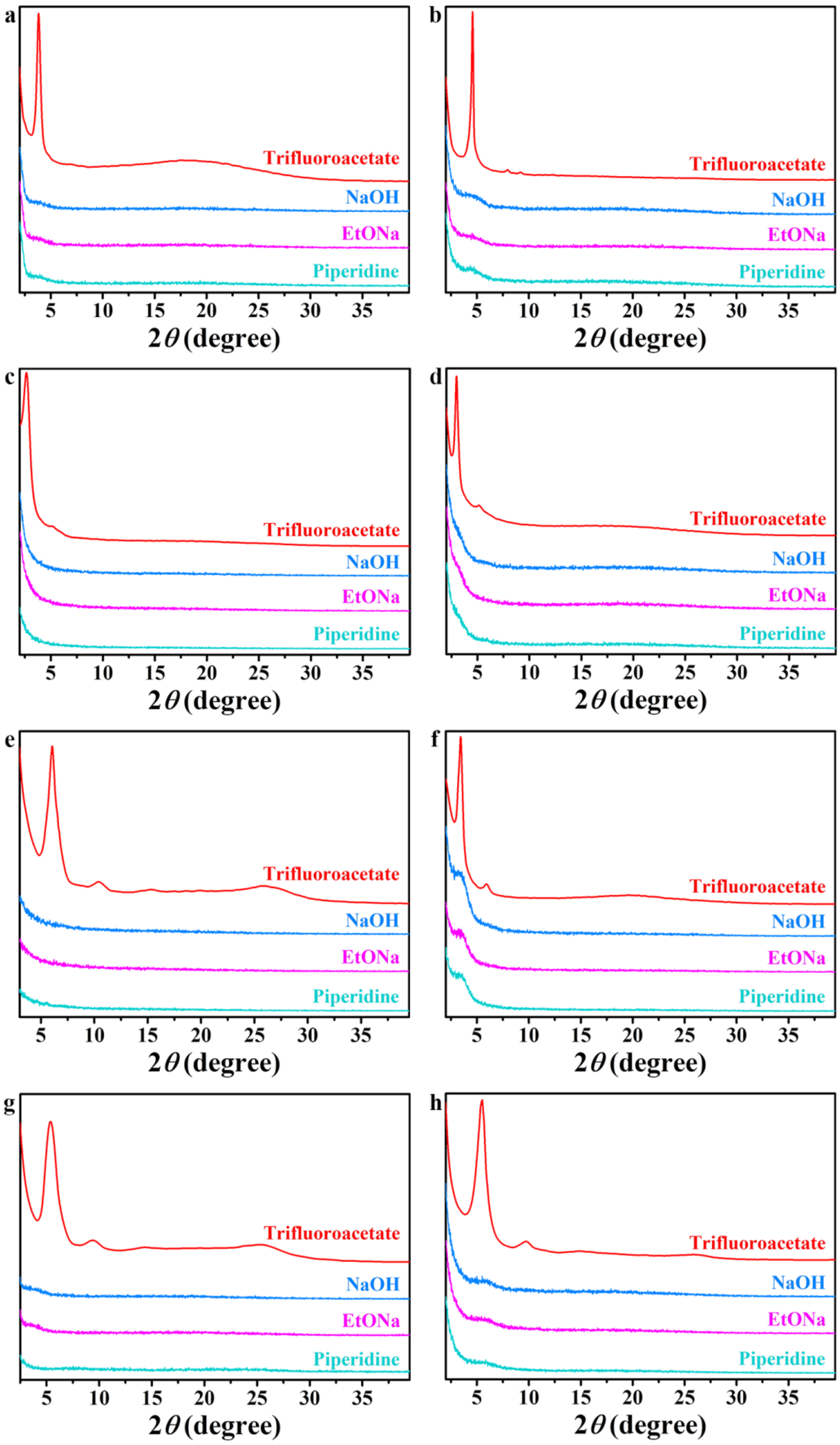

Supplementary Fig. 2 | PXRD patterns of attempts to synthesize DAFB-TMT (a), BTTA-TMT (b), TDA-TMT (c), EDA-TMT (d), TPA-TMT (e), BDA-TMT (f), TFPB-TMT (g), and TFPT-TMT (h) under different catalyst conditions.

TFPT-TMT was synthesized based on the reported method.[4]

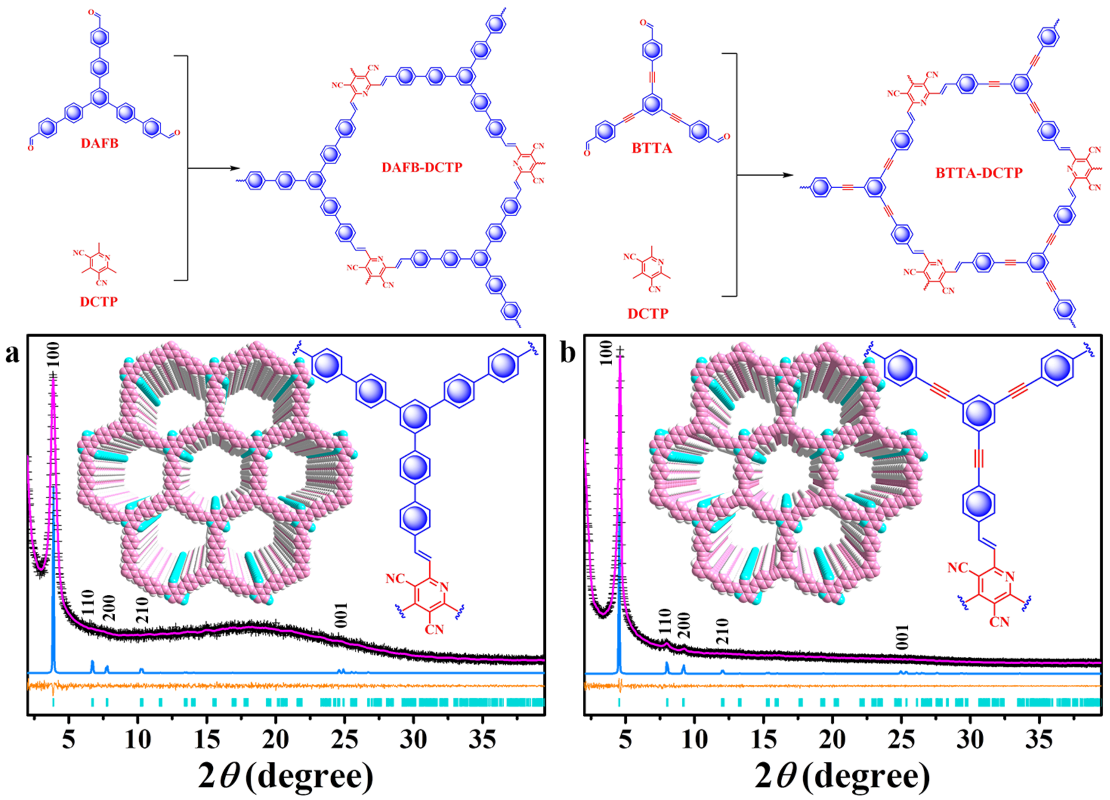

Supplementary Fig. 3 | PXRD patterns of DAFB-DCTP (a) and BTTA-DCTP (b): comparison between the experimental (black cross) and Pawley refined (pink line) profiles, the eclipsed stacking mode (blue line), the refinement differences (orange line), and the Bragg positions (green bar). Their crystal structures were elucidated by comparing the diffraction results with the structural simulations. The PXRD patterns of DAFB-DCTP and BTTA-DCTP showed highly intense reflection peaks at about 3.83 and 4.51 (2θ), respectively, which could be ascribed to the (100) facet, indicating the highly crystalline skeleton of DAFB-DCTP and BTTA-DCTP. The experimental PXRD patterns agreed well with the simulated AA stacking models, as demonstrated by the negligible difference, suggesting the validity of the slipped eclipsed AA stacking.

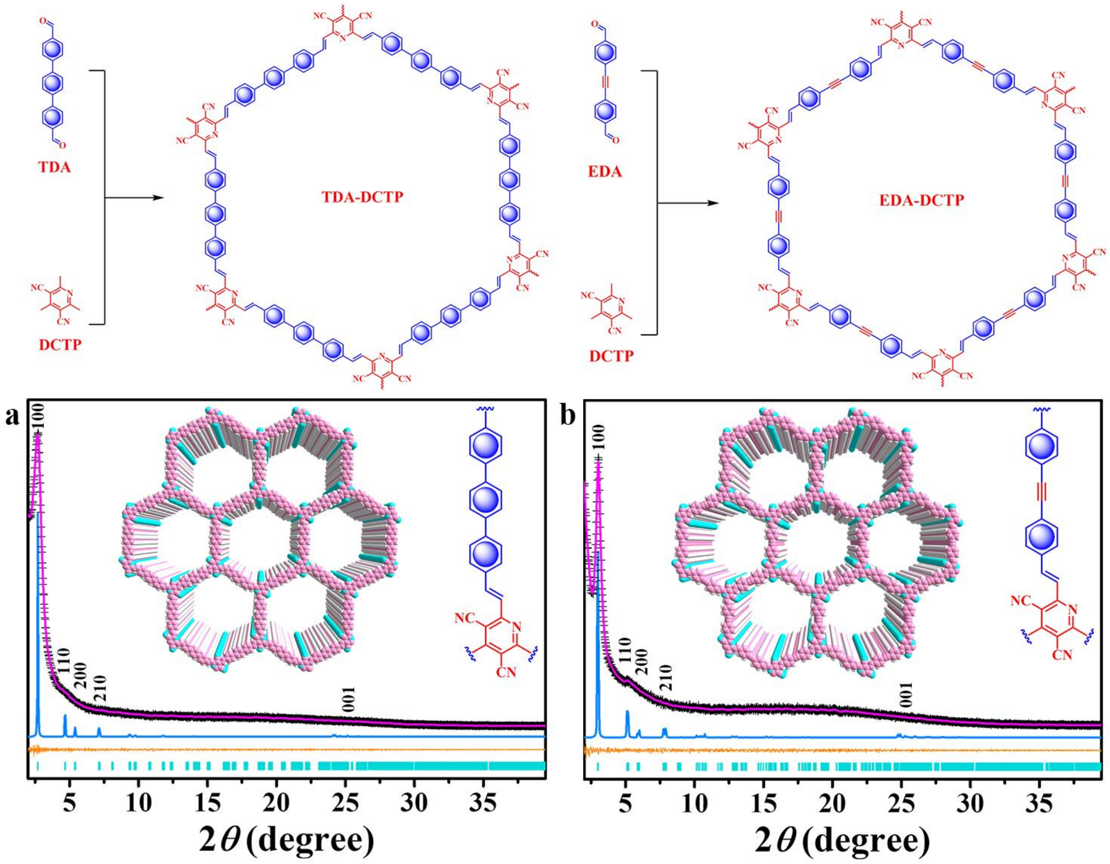

Supplementary Fig. 4 | PXRD patterns of TDA-DCTP (a) and EDA-DCTP (b): comparison between the experimental (black cross) and Pawley refined (pink line) profiles, the eclipsed stacking mode (blue line), the refinement differences (orange line), and the Bragg positions (green bar). Their crystal structures were elucidated by comparing the diffraction results with the structural simulations. The PXRD patterns of TDA-DCTP and EDA-DCTP showed highly intense reflection peaks at about 2.71 and 3.00 (2θ), respectively, which could be ascribed to the (100) facet, indicating the highly crystalline skeleton of TDA-DCTP and EDA-DCTP. The experimental PXRD patterns agreed well with the simulated AA stacking models, as demonstrated by the negligible difference, suggesting the validity of the slipped eclipsed AA stacking.

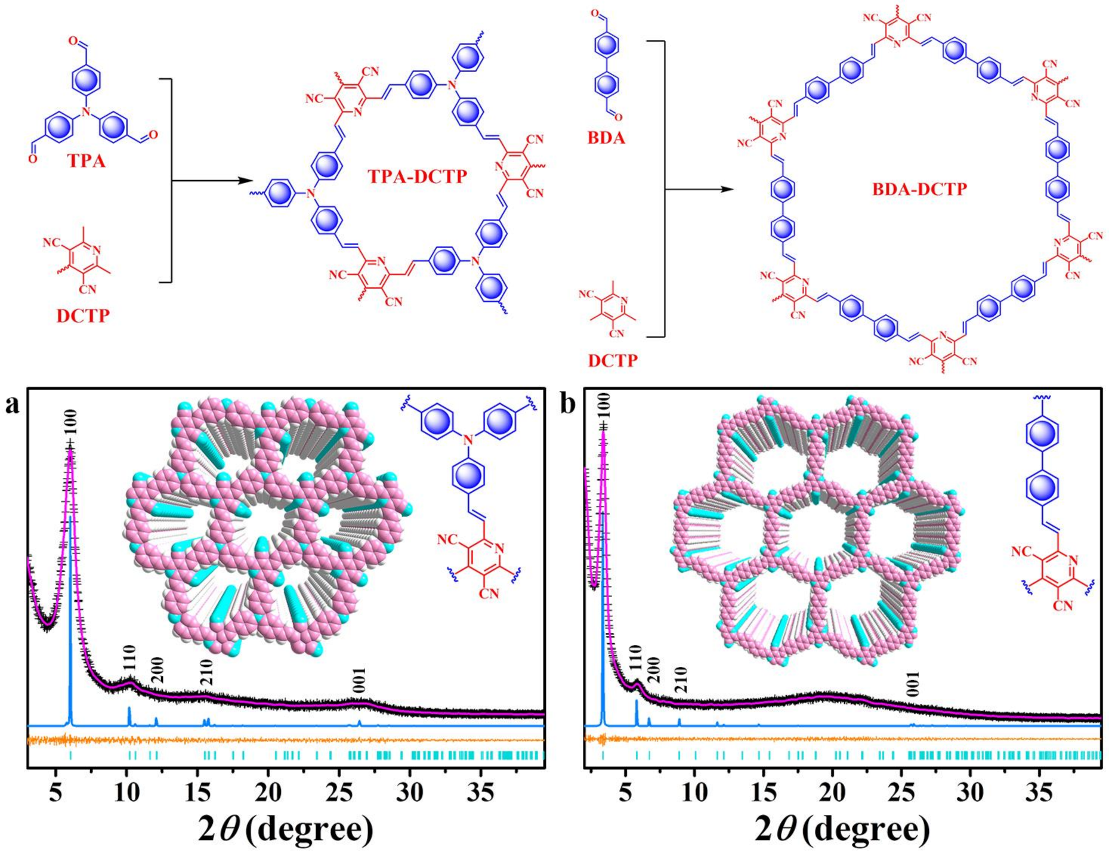

Supplementary Fig. 5 | PXRD patterns of TPA-DCTP (a) and BDA-DCTP (b): comparison between the experimental (black cross) and Pawley refined (pink line) profiles, the eclipsed stacking mode (blue line), the refinement differences (orange line), and the Bragg positions (green bar). Their crystal structures were elucidated by comparing the diffraction results with the structural simulations. The PXRD patterns of TPA-DCTP and BDA-DCTP showed highly intense reflection peaks at about 6.09 and 3.35 (2θ), respectively, which could be ascribed to the (100) facet, indicating the highly crystalline skeleton of TPA-DCTP and BDA-DCTP. The experimental PXRD patterns agreed well with the simulated AA stacking models, as demonstrated by the negligible difference, suggesting the validity of the slipped eclipsed AA stacking.

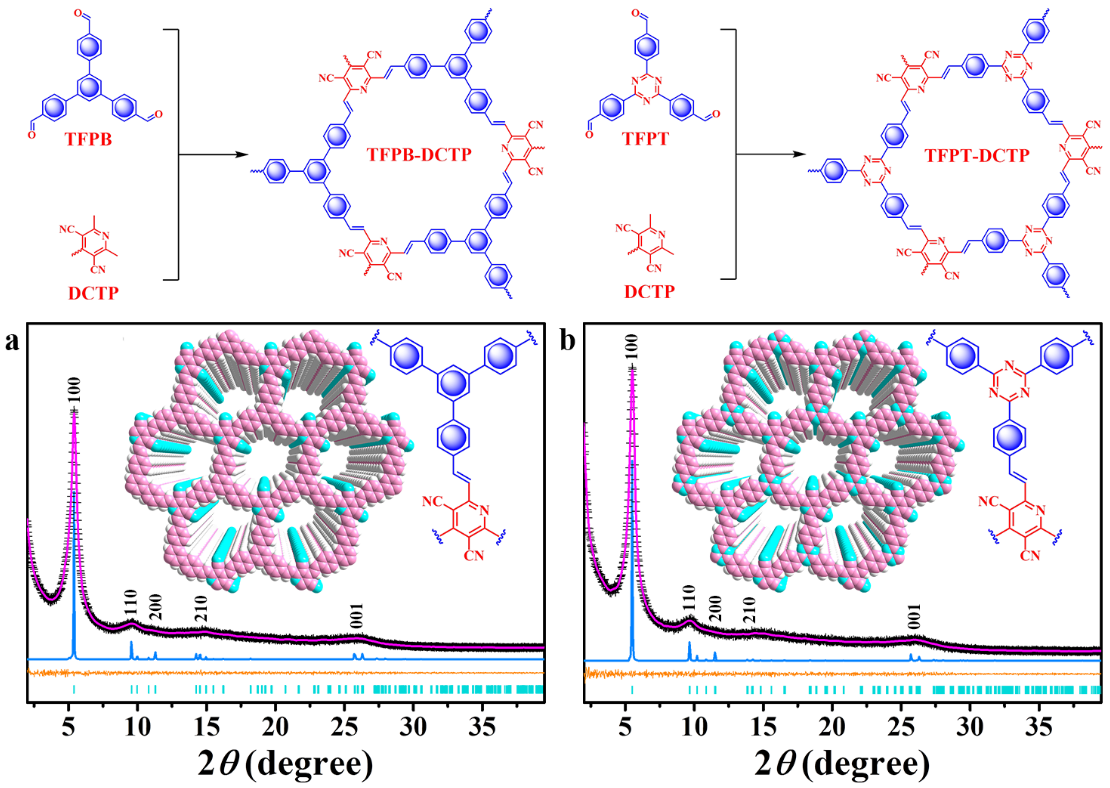

Supplementary Fig. 6 | PXRD patterns of TFPB-DCTP (a) and TFPT-DCTP (b): comparison between the experimental (black cross) and Pawley refined (pink line) profiles, the eclipsed stacking mode (blue line), the refinement differences (orange line), and the Bragg positions (green bar). Their crystal structures were elucidated by comparing the diffraction results with the structural simulations. The PXRD patterns of TFPB-DCTP and TFPT-DCTP showed highly intense reflection peaks at about 5.41 and 5.50 (2θ), respectively, which could be ascribed to the (100) facet, indicating the highly crystalline skeleton of TFPB-DCTP and TFPT-DCTP. The experimental PXRD patterns agreed well with the simulated AA stacking models, as demonstrated by the negligible difference, suggesting the validity of the slipped eclipsed AA stacking.

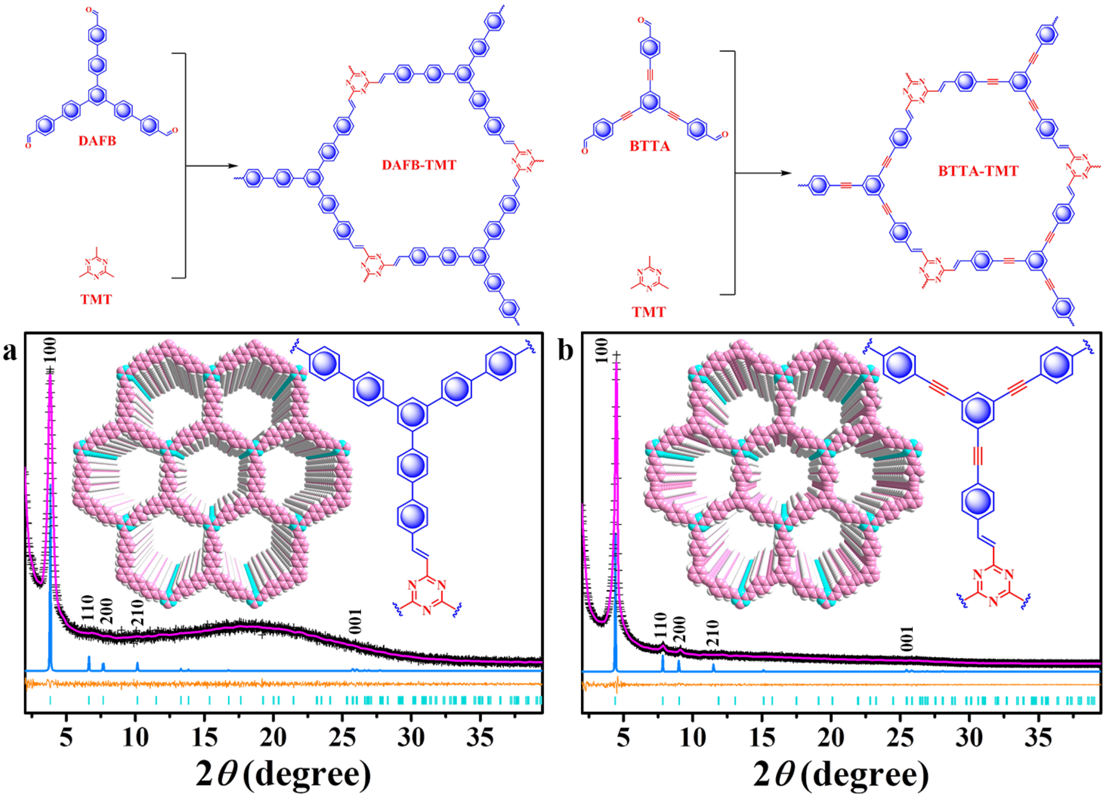

Supplementary Fig. 7 | PXRD patterns of DAFB-TMT (a) and BTTA-TMT (b): comparison between the experimental (black cross) and Pawley refined (pink line) profiles, the eclipsed stacking mode (blue line), the refinement differences (orange line), and the Bragg positions (green bar). Their crystal structures were elucidated by comparing the diffraction results with the structural simulations. The PXRD patterns of DAFB-TMT and BTTA-TMT showed highly intense reflection peaks at about 3.80 and 4.38 (2θ), respectively, which could be ascribed to the (100) facet, indicating the highly crystalline skeleton of DAFB-TMT and BTTA-TMT. The experimental PXRD patterns agreed well with the simulated AA stacking models, as demonstrated by the negligible difference, suggesting the validity of the slipped eclipsed AA stacking.

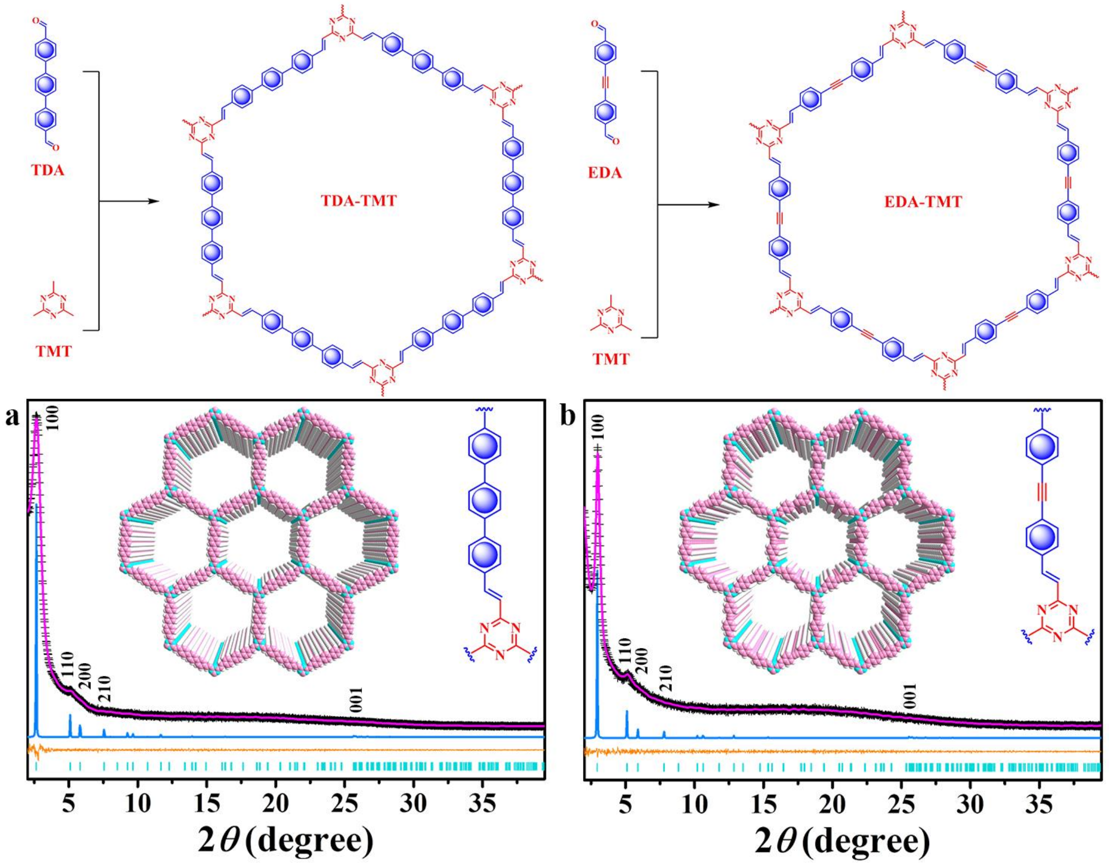

Supplementary Fig. 8 | PXRD patterns of TDA-TMT (a) and EDA-TMT (b): comparison between the experimental (black cross) and Pawley refined (pink line) profiles, the eclipsed stacking mode (blue line), the refinement differences (orange line), and the Bragg positions (green bar). Their crystal structures were elucidated by comparing the diffraction results with the structural simulations. The PXRD patterns of TDA-TMT and EDA-TMT showed highly intense reflection peaks at about 2.60 and 2.92 (2θ), respectively, which could be ascribed to the (100) facet, indicating the highly crystalline skeleton of TDA-TMT and EDA-TMT. The experimental PXRD patterns agreed well with the simulated AA stacking models, as demonstrated by the negligible difference, suggesting the validity of the slipped eclipsed AA stacking.

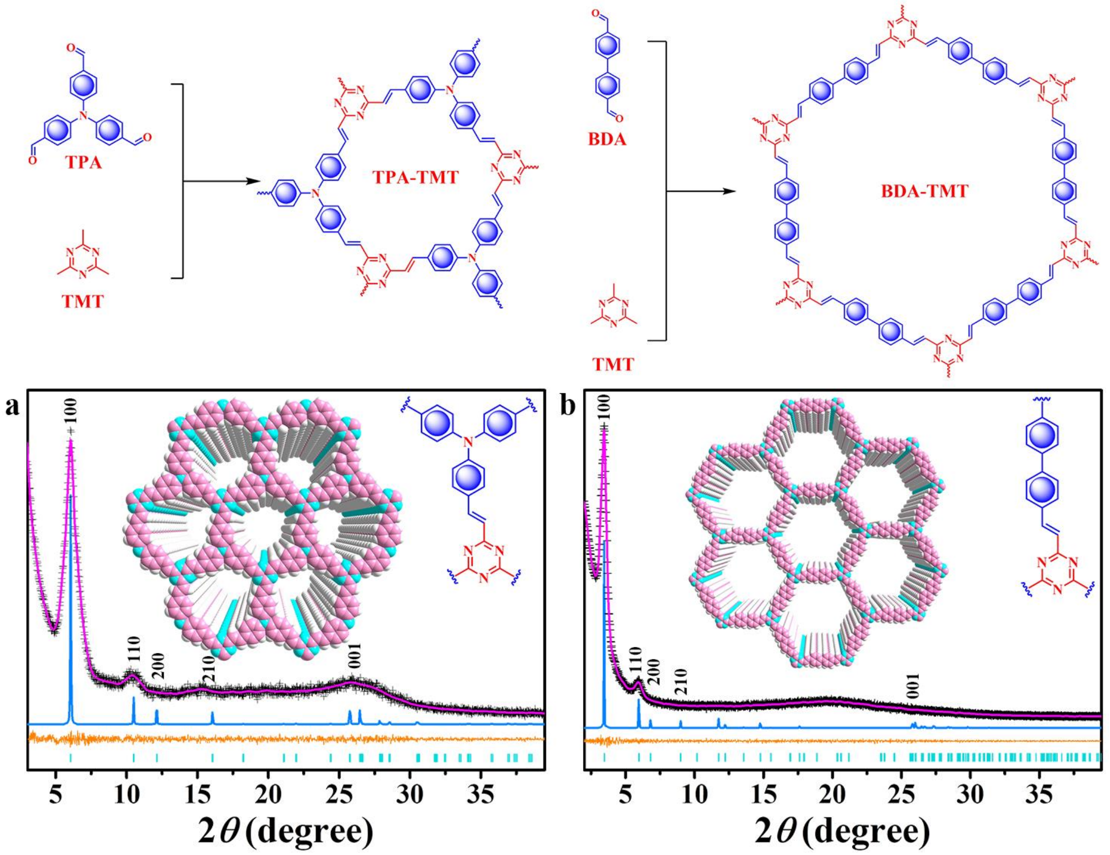

Supplementary Fig. 9 | PXRD patterns of TPA-TMT (a) and BDA-TMT (b): comparison between the experimental (black cross) and Pawley refined (pink line) profiles, the eclipsed stacking mode (blue line), the refinement differences (orange line), and the Bragg positions (green bar). Their crystal structures were elucidated by comparing the diffraction results with the structural simulations. The PXRD patterns of TPA-TMT and BDA-TMT showed highly intense reflection peaks at about 6.01 and 3.44 (2θ), respectively, which could be ascribed to the (100) facet, indicating the highly crystalline skeleton of TPA-TMT and BDA-TMT. The experimental PXRD patterns agreed well with the simulated AA stacking models, as demonstrated by the negligible difference, suggesting the validity of the slipped eclipsed AA stacking.

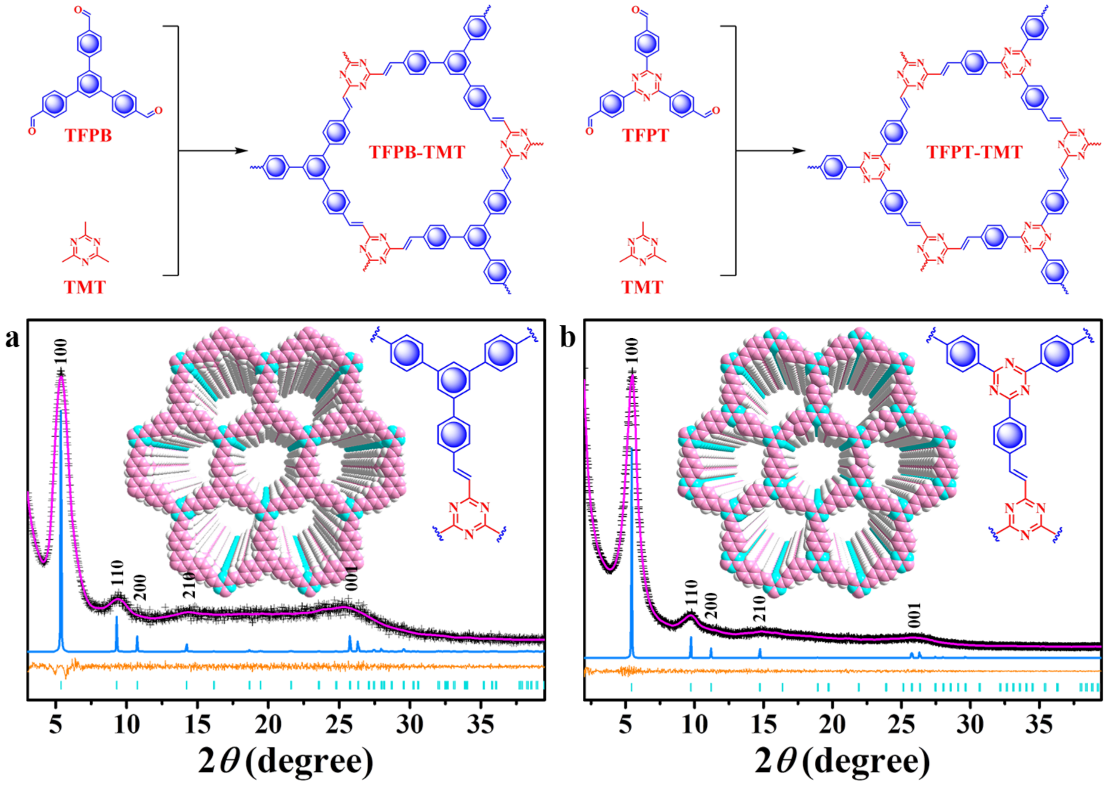

Supplementary Fig. 10 | PXRD patterns of TFPB-TMT (a) and TFPT-TMT (b): comparison between the experimental (black cross) and Pawley refined (pink line) profiles, the eclipsed stacking mode (blue line), the refinement differences (orange line), and the Bragg positions (green bar). Their crystal structures were elucidated by comparing the diffraction results with the structural simulations. The PXRD patterns of TFPB-TMT and TFPT-TMT showed highly intense reflection peaks at about 5.37 and 5.41 (2θ), respectively, which could be ascribed to the (100) facet, indicating the highly crystalline skeleton of TFPB-TMT and TFPT-TMT. The experimental PXRD patterns agreed well with the simulated AA stacking models, as demonstrated by the negligible difference, suggesting the validity of the slipped eclipsed AA stacking.

Supplementary Table 1 | Fractional atomic coordinates for the eclipsed AA-stacking unit cell of TPA-DCTP.

Space group: PM<br>***a = 17.3171 Å, b = 3.4619 Å, c = 17.3333 Å***<br>***α = γ = 90°, β = 122.5481°***							
**Atom**	***x*** **(Å)**	***y*** **(Å)**	***z*** **(Å)**	**Atom**	***x*** **(Å)**	***y*** **(Å)**	***z*** **(Å)**
N1	0.57824	2.5	1.41199	C28	0.74114	2.5	1.5783
C2	0.51685	2.5	1.45288	C29	0.43488	2.5	1.23348
C3	0.41832	2.5	1.40235	C30	0.39424	2.5	1.13733
C4	0.36237	2.5	1.43811	C31	0.44731	2.5	1.10034
C5	0.39878	2.5	1.53078	C32	0.54015	2.5	1.16116
C6	0.49315	2.5	1.58622	C33	0.57905	2.5	1.256
C7	0.54779	2.5	1.54852	C34	0.99487	2.5	1.65971
C8	0.33475	2.5	1.56348	C35	0.413	2.5	1.00111
C9	0.35957	2.5	1.65174	H36	0.37111	2.5	1.33509
C10	0.29894	2.5	1.69122	H37	0.28826	2.5	1.3918
C11	0.34315	2.5	1.78875	H38	0.52674	2.5	1.65987
C12	0.29015	2.5	1.82988	H39	0.61118	2.5	1.60467
N13	0.19741	2.5	1.77419	H40	0.26481	2.5	1.50906
C14	0.15171	2.5	1.68164	H41	0.43205	2.5	1.70044
C15	0.20121	2.5	1.6379	H42	0.27195	2.5	1.94515
C16	0.32511	2.5	1.93045	H43	0.01859	2.5	1.55437
C17	0.04956	2.5	1.62663	H44	0.74044	2.5	1.38892
C18	0.44208	2.5	1.84342	H45	0.88139	2.5	1.4696
N19	0.52146	2.5	1.88546	H46	0.87211	2.5	1.70922
C20	0.14857	2.5	1.53905	H47	0.72605	2.5	1.62763
N21	0.10403	2.5	1.45978	H48	0.37673	2.5	1.23433
C22	0.68562	2.5	1.47836	H49	0.32029	2.5	1.0918
C23	0.53238	2.5	1.30471	H50	0.58407	2.5	1.13377
C24	0.74968	2.5	1.45031	H51	0.64495	2.5	1.27319
C25	0.84542	2.5	1.5047	H52	1.02618	2.5	1.7332
C26	0.89218	2.5	1.59892	H53	0.467	2.5	0.98924
C27	0.83826	2.5	1.63517				

Supplementary Table 2 | Fractional atomic coordinates for the eclipsed AA-stacking unit cell of TFPT-DCTP.

Space group: PM ***a = 19.3832 Å, b = 3.4632 Å, c = 19.3021 Å*** ***α = γ = 90°, β = 123.7707°***							
**Atom**	***x* (Å)**	***y* (Å)**	***z* (Å)**	**Atom**	***x* (Å)**	***y* (Å)**	***z* (Å)**
C1	-0.53188	2.5	-0.49815	C30	-0.1792	2.5	-0.41873
C2	-0.58325	2.5	-0.46295	C31	-0.13258	2.5	-0.33087
C3	-0.67022	2.5	-0.51614	C32	-0.17601	2.5	-0.2918
C4	-0.71905	2.5	-0.48373	C33	-0.26333	2.5	-0.33914
C5	-0.68275	2.5	-0.39728	C34	-0.6489	2.5	-0.76426
C6	-0.59534	2.5	-0.34375	C35	-0.68344	2.5	-0.85043
C7	-0.54639	2.5	-0.37639	C36	-0.63159	2.5	-0.88033
C8	-0.73969	2.5	-0.36813	C37	-0.54533	2.5	-0.8224
C9	-0.71579	2.5	-0.28786	C38	-0.5112	2.5	-0.73721
C10	-0.76929	2.5	-0.2523	C39	-0.0397	2.5	-0.27862
C11	-0.72768	2.5	-0.16344	C40	-0.66172	2.5	-0.96993
C12	-0.7743	2.5	-0.12641	H41	-0.70061	2.5	-0.58314
N13	-0.8583	2.5	-0.17733	H42	-0.78594	2.5	-0.52676
C14	-0.90121	2.5	-0.26153	H43	-0.56348	2.5	-0.27686
C15	-0.85794	2.5	-0.30119	H44	-0.47947	2.5	-0.33387
C16	-0.74147	2.5	-0.03517	H45	-0.8034	2.5	-0.41795
C17	-0.99367	2.5	-0.31103	H46	-0.64995	2.5	-0.24322
N18	-0.44749	2.5	-0.44703	H47	-0.78934	2.5	-0.02279
C19	-0.40113	2.5	-0.4798	H48	-1.02452	2.5	-0.37726
N20	-0.43986	2.5	-0.56349	H49	-0.29814	2.5	-0.53261
C21	-0.52337	2.5	-0.61524	H50	-0.15139	2.5	-0.45387
N22	-0.56934	2.5	-0.58224	H51	-0.14227	2.5	-0.22448
C23	-0.63806	2.5	-0.11296	H52	-0.29531	2.5	-0.30766
N24	-0.56618	2.5	-0.07408	H53	-0.68964	2.5	-0.7424
C25	-0.90724	2.5	-0.39115	H54	-0.75014	2.5	-0.89252
N26	-0.94864	2.5	-0.46332	H55	-0.50361	2.5	-0.84314
C27	-0.30889	2.5	-0.42671	H56	-0.44426	2.5	-0.69491
C28	-0.56225	2.5	-0.70675	H57	-0.00828	2.5	-0.21146
C29	-0.26535	2.5	-0.46534	H58	-0.61255	2.5	-0.97921

Supplementary Table 3 | Fractional atomic coordinates for the eclipsed AA-stacking unit cell of TFPB-DCTP.

Space group: PM							
a = 19.5434 Å, b = 3.4607 Å, c = 19.5168 Å							
α = γ = 90°, β = 122.9112°							
**Atom**	***x*** **(Å)**	***y*** **(Å)**	***z*** **(Å)**	**Atom**	***x*** **(Å)**	***y*** **(Å)**	***z*** **(Å)**
C1	0.50048	2.5	0.50577	C32	0.85887	2.5	0.71226
C2	0.44834	2.5	0.54212	C33	0.77303	2.5	0.66423
C3	0.36207	2.5	0.49202	C34	0.38464	2.5	0.23563
C4	0.3143	2.5	0.5251	C35	0.34948	2.5	0.15075
C5	0.34953	2.5	0.60952	C36	0.39886	2.5	0.11893
C6	0.43498	2.5	0.66055	C37	0.48329	2.5	0.17354
C7	0.48324	2.5	0.6276	C38	0.51837	2.5	0.25735
C8	0.29345	2.5	0.63919	C39	0.99576	2.5	0.72886
C9	0.31657	2.5	0.71805	C40	0.36828	2.5	0.03062
C10	0.26363	2.5	0.75379	H41	0.32852	2.5	0.42658
C11	0.30401	2.5	0.84085	H42	0.24859	2.5	0.48377
C12	0.25782	2.5	0.87798	H43	0.46605	2.5	0.72613
N13	0.17528	2.5	0.82876	H44	0.54791	2.5	0.67134
C14	0.13363	2.5	0.74623	H45	0.23099	2.5	0.59089
C15	0.17663	2.5	0.70669	H46	0.3812	2.5	0.76117
C16	0.28989	2.5	0.96769	H47	0.24302	2.5	0.98099
C17	0.04277	2.5	0.69798	H48	0.0138	2.5	0.63331
C18	0.58681	2.5	0.55599	H49	0.6162	2.5	0.62053
C19	0.63606	2.5	0.52358	H50	0.6308	2.5	0.41229
C20	0.59553	2.5	0.43791	H51	0.39984	2.5	0.38053
C21	0.51027	2.5	0.38435	H52	0.75196	2.5	0.47881
C22	0.46423	2.5	0.42012	H53	0.89063	2.5	0.55731
C23	0.39203	2.5	0.88928	H54	0.88985	2.5	0.77808
N24	0.46263	2.5	0.92647	H55	0.74288	2.5	0.69676
C25	0.12875	2.5	0.61857	H56	0.34264	2.5	0.25506
N26	0.08845	2.5	0.5479	H57	0.2839	2.5	0.11089
C27	0.72918	2.5	0.57765	H58	0.52334	2.5	0.15115
C28	0.47056	2.5	0.29183	H59	0.5837	2.5	0.29234
C29	0.77678	2.5	0.54309	H60	1.02502	2.5	0.79453
C30	0.86129	2.5	0.59061	H61	0.41624	2.5	0.0202
C31	0.90442	2.5	0.67622				

Supplementary Table 4 | Fractional atomic coordinates for the eclipsed AA-stacking unit cell of DAFB-DCTP.

Space group: PM ***a = 26.4646 Å, b = 3.6192 Å, c = 26.1299 Å*** ***α = γ = 90°, β = 120°***							
**Atom**	***x* (Å)**	***y* (Å)**	***z* (Å)**	**Atom**	***x* (Å)**	***y* (Å)**	***z* (Å)**
C1	0.40275	0.5	0.55218	C47	0.88455	0.5	0.6793
C2	0.36596	0.5	0.58028	C48	0.33163	0.5	0.08768
C3	0.30524	0.5	0.54636	C49	0.30276	0.5	0.02585
C4	0.27056	0.5	0.57262	C50	0.33451	0.5	-0.00361
C5	0.29599	0.5	0.63339	C51	0.39521	0.5	0.02945
C6	0.35647	0.5	0.66756	C52	0.42395	0.5	0.09111
C7	0.39112	0.5	0.64116	C53	1.04322	0.5	0.72432
C8	0.25833	0.5	0.65979	C54	0.30652	0.5	-0.06846
C9	0.27351	0.5	0.71181	C55	0.3236	0.5	0.83424
C10	0.23612	0.5	0.7395	N56	0.37204	0.5	0.86055
C11	0.26293	0.5	0.80119	C57	0.14668	0.5	0.64459
C12	0.2291	0.5	0.82914	N58	0.12377	0.5	0.59508
N13	0.17013	0.5	0.79529	H59	0.2846	0.5	0.49966
C14	0.14221	0.5	0.73534	H60	0.22389	0.5	0.5458
C15	0.17517	0.5	0.70655	H61	0.37665	0.5	0.71434
C16	0.25466	0.5	0.89428	H62	0.43744	0.5	0.66824
C17	0.07753	0.5	0.70058	H63	0.21658	0.5	0.63453
C18	0.45913	0.5	0.58258	H64	0.3158	0.5	0.73632
C19	0.49324	0.5	0.55568	H65	0.22902	0.5	0.91136
C20	0.47194	0.5	0.4978	H66	0.05744	0.5	0.65357
C21	0.41578	0.5	0.46772	H67	0.47613	0.5	0.62644
C22	0.38163	0.5	0.49452	H68	0.5355	0.5	0.57952
C23	0.50768	0.5	0.46856	H69	0.39868	0.5	0.42391
C24	0.4809	0.5	0.40763	H70	0.33923	0.5	0.4706
C25	0.51332	0.5	0.37843	H71	0.43442	0.5	0.38293
C26	0.57463	0.5	0.41268	H72	0.60045	0.5	0.39133
C27	0.60284	0.5	0.47452	H73	0.5892	0.5	0.54785
C28	0.56851	0.5	0.5013	H74	0.66939	0.5	0.58753
C29	0.66786	0.5	0.51169	H75	0.77506	0.5	0.64846
C30	0.48248	0.5	0.3124	H76	0.79061	0.5	0.50659
C31	0.69499	0.5	0.56964	H77	0.68467	0.5	0.44629
C32	0.7557	0.5	0.60465	H78	0.55162	0.5	0.30678
C33	0.79108	0.5	0.58237	H79	0.50136	0.5	0.19955
C34	0.76435	0.5	0.52452	H80	0.35409	0.5	0.19217
C35	0.70353	0.5	0.48979	H81	0.40458	0.5	0.29878
C36	0.5091	0.5	0.28262	H82	0.86747	0.5	0.54776
C37	0.48008	0.5	0.2208	H83	0.97298	0.5	0.60703
C38	0.42347	0.5	0.18697	H84	0.96685	0.5	0.75944
C39	0.39673	0.5	0.21661	H85	0.86013	0.5	0.69979
C40	0.42587	0.5	0.27822	H86	0.30626	0.5	0.10924
C41	0.8556	0.5	0.61891	H87	0.25605	0.5	0.00117
C42	0.39264	0.5	0.12115	H88	0.4203	0.5	0.00726
C43	0.88869	0.5	0.59367	H89	0.47064	0.5	0.11518
C44	0.94936	0.5	0.62772	H90	1.06293	0.5	0.77186
C45	0.97859	0.5	0.68773	H91	0.3313	0.5	-0.08555
C46	0.94545	0.5	0.71335				

Supplementary Table 5 | Fractional atomic coordinates for the eclipsed AA-stacking unit cell of BDA-DCTP.

Space group: P2/M ***a = 30.6081 Å, b = 3.6028 Å, c = 29.6434 Å*** ***α = γ = 90°, β = 120°***							
**Atom**	***x* (Å)**	***y* (Å)**	***z* (Å)**	**Atom**	***x* (Å)**	***y* (Å)**	***z* (Å)**
C1	1.48197	0.5	1.50989	C27	1.04913	0.5	1.56983
C2	1.43109	0.5	1.47703	C28	1.02781	0.5	1.51765
C3	1.39728	0.5	1.49569	C29	1.06011	0.5	1.49921
C4	1.41395	0.5	1.54779	C30	1.1124	0.5	1.53159
C5	1.4645	0.5	1.58077	C31	1.57222	0.5	1.28124
C6	1.49809	0.5	1.56188	C32	1.71861	0.5	1.44948
C7	1.37786	0.5	1.56676	N33	1.73684	0.5	1.49308
C8	1.38995	0.5	1.61298	N34	1.53139	0.5	1.25765
C9	1.35569	0.5	1.63514	H35	1.41755	0.5	1.43716
C10	1.37668	0.5	1.689	H36	1.35832	0.5	1.4699
C11	1.34662	0.5	1.71245	H37	1.47768	0.5	1.62081
N12	1.29645	0.5	1.68166	H38	1.53646	0.5	1.58781
C13	1.27412	0.5	1.6291	H39	1.34076	0.5	1.54148
C14	1.30384	0.5	1.605	H40	1.42765	0.5	1.63716
C15	1.36714	0.5	1.7696	H41	1.34297	0.5	1.78298
C16	1.21876	0.5	1.59733	H42	1.20151	0.5	1.55573
C17	1.41335	0.5	1.80429	H43	1.43689	0.5	1.79109
C18	1.43648	0.5	1.86101	H44	1.20515	0.5	1.65895
C19	1.18901	0.5	1.6171	H45	1.5083	0.5	1.86995
C20	1.1337	0.5	1.58314	H46	1.55114	0.5	1.96486
C21	1.48752	0.5	1.88977	H47	1.41424	0.5	1.96084
C22	1.51208	0.5	1.94405	H48	1.37101	0.5	1.86565
C23	1.48645	0.5	1.97093	H49	1.11704	0.5	1.64181
C24	1.43497	0.5	1.94168	H50	1.02527	0.5	1.58506
C25	1.4101	0.5	1.88718	H51	1.04489	0.5	1.45985
C26	1.10156	0.5	1.60227	H52	1.13603	0.5	1.51627

Supplementary Table 6 | Fractional atomic coordinates for the eclipsed AA-stacking unit cell of TDA-DCTP.

Space group: P2/M ***a = 37.9761 Å, b = 3.6924 Å, c = 37.5870 Å*** ***α = γ = 90°, β = 120°***							
**Atom**	***x* (Å)**	***y* (Å)**	***z* (Å)**	**Atom**	***x* (Å)**	***y* (Å)**	***z* (Å)**
C1	0.47568	0.5	1.51894	C35	0.08808	0.5	1.5539
C2	0.4501	0.5	1.53886	C36	0.11385	0.5	1.54085
C3	0.41063	0.5	1.51896	C37	0.15606	0.5	1.56567
C4	0.3865	0.5	1.53731	C38	0.479	0.5	1.95632
C5	0.40123	0.5	1.57631	C39	0.04291	0.5	1.52632
C6	0.44095	0.5	1.59671	C40	1.45757	0.5	0.97787
C7	0.46479	0.5	1.57801	C41	1.47821	0.5	1.02079
C8	0.37412	0.5	1.59415	C42	0.97447	0.5	0.5167
C9	0.38204	0.5	1.62869	C43	1.01664	0.5	0.54256
C10	0.35418	0.5	1.64579	H44	0.39851	0.5	1.48958
C11	0.37077	0.5	1.68819	H45	0.35627	0.5	1.52115
C12	0.34558	0.5	1.70578	H46	0.45395	0.5	1.62686
N13	0.30505	0.5	1.68102	H47	0.49439	0.5	1.59402
C14	0.28749	0.5	1.6397	H48	0.34605	0.5	1.57776
C15	0.31208	0.5	1.62134	H49	0.41024	0.5	1.64665
C16	0.36142	0.5	1.75056	H50	0.34293	0.5	1.76002
C17	0.24283	0.5	1.61457	H51	0.22934	0.5	1.58154
C18	0.51839	0.5	1.54272	H52	0.53366	0.5	1.57588
C19	0.54229	0.5	1.5241	H53	0.57494	0.5	1.54373
C20	0.41262	0.5	1.71263	H54	0.41233	0.5	1.76675
N21	0.44601	0.5	1.73216	H55	0.2312	0.5	1.66356
C22	0.29444	0.5	1.57831	H56	0.47024	0.5	1.83243
N23	0.28027	0.5	1.5439	H57	0.50503	0.5	1.90678
C24	0.39626	0.5	1.77809	H58	0.40252	0.5	1.90077
C25	0.41574	0.5	1.82338	H59	0.36663	0.5	1.82662
C26	0.21843	0.5	1.63062	H60	0.1613	0.5	1.64826
C27	0.17378	0.5	1.60449	H61	0.08764	0.5	1.60445
C28	0.45514	0.5	1.84711	H62	0.10158	0.5	1.51177
C29	0.47539	0.5	1.8899	H63	0.17471	0.5	1.55474
C30	0.45703	0.5	1.91041	H64	1.42473	0.5	0.96178
C31	0.41744	0.5	1.88651	H65	1.46035	0.5	1.03589
C32	0.39689	0.5	1.84356	H66	0.95563	0.5	0.53072
C33	0.14836	0.5	1.6185	H67	1.02846	0.5	0.57535
C34	0.10605	0.5	1.59338				

Supplementary Table 7 | Fractional atomic coordinates for the eclipsed AA-stacking unit cell of BTTA-DCTP.

Space group: PM ***a = 23.4169 Å, b = 3.5833 Å, c = 23.1605 Å*** ***α = γ = 90°, β = 120°***							
**Atom**	***x* (Å)**	***y* (Å)**	***z* (Å)**	**Atom**	***x* (Å)**	***y* (Å)**	***z* (Å)**
C1	-0.48934	0.5	0.37244	C35	-0.76939	0.5	0.8386
C2	-0.52683	0.5	0.40387	N36	-0.83578	0.5	0.79913
C3	-0.49582	0.5	0.47344	C37	-0.86578	0.5	0.7317
C4	-0.42721	0.5	0.51181	C38	-0.82709	0.5	0.70073
C5	-0.38944	0.5	0.48065	C39	-0.74227	0.5	0.91169
C6	-0.42051	0.5	0.41084	C40	-0.93873	0.5	0.69133
C7	-0.53463	0.5	0.5054	C41	-0.85769	0.5	0.63068
C8	-0.31886	0.5	0.5208	N42	-0.88225	0.5	0.5747
C9	-0.52217	0.5	0.30079	C43	-0.68511	0.5	-0.04459
C10	-0.25978	0.5	0.55532	C44	0.02213	0.5	0.71736
C11	-0.5675	0.5	0.53188	C45	-0.66079	0.5	0.84755
C12	-0.55088	0.5	0.24071	N46	-0.6059	0.5	0.8785
C13	-0.60702	0.5	0.56315	H47	-0.58004	0.5	0.37426
C14	-0.58541	0.5	0.16902	H48	-0.4033	0.5	0.56575
C15	-0.67459	0.5	0.52469	H49	-0.39139	0.5	0.38659
C16	-0.71338	0.5	0.55487	H50	-0.6972	0.5	0.47141
C17	-0.68513	0.5	0.62369	H51	-0.76573	0.5	0.52439
C18	-0.61678	0.5	0.66228	H52	-0.59283	0.5	0.71577
C19	-0.5781	0.5	0.63196	H53	-0.52569	0.5	0.66207
C20	-0.55184	0.5	0.13595	H54	-0.49993	0.5	0.16401
C21	-0.58527	0.5	0.06642	H55	-0.55839	0.5	0.04172
C22	-0.65266	0.5	0.02886	H56	-0.73836	0.5	0.03547
C23	-0.6863	0.5	0.06245	H57	-0.67905	0.5	0.15754
C24	-0.65268	0.5	0.13222	H58	-0.18757	0.5	0.6859
C25	-0.18922	0.5	0.59654	H59	-0.06642	0.5	0.7551
C26	-0.1584	0.5	0.66405	H60	-0.05451	0.5	0.58546
C27	-0.08955	0.5	0.70334	H61	-0.17463	0.5	0.51697
C28	-0.05078	0.5	0.6754	H62	-0.77388	0.5	0.62586
C29	-0.08244	0.5	0.60798	H63	-0.66876	0.5	0.73811
C30	-0.15114	0.5	0.56884	H64	-0.77131	0.5	0.92851
C31	-0.72829	0.5	0.65319	H65	-0.96103	0.5	0.63824
C32	-0.7144	0.5	0.71017	H66	-0.65893	0.5	-0.06348
C33	-0.75824	0.5	0.73947	H67	0.0439	0.5	0.77096
C34	-0.72955	0.5	0.80876				

Supplementary Table 8 | Fractional atomic coordinates for the eclipsed AA-stacking unit cell of EDA-DCTP.

Space group: P2/M ***a = 35.0731 Å, b = 3.6041 Å, c = 34.2450 Å*** ***α = γ = 90°, β = 120°***							
**Atom**	***x* (Å)**	***y* (Å)**	***z* (Å)**	**Atom**	***x* (Å)**	***y* (Å)**	***z* (Å)**
C1	0.46025	0.5	1.52756	C29	0.09304	0.5	1.52213
C2	0.41518	0.5	1.50053	C30	0.13871	0.5	1.5492
C3	0.38798	0.5	1.5195	C31	0.51175	0.5	1.49187
C4	0.40549	0.5	1.56564	C32	0.4902	0.5	1.9798
C5	0.45103	0.5	1.59273	C33	0.01964	0.5	1.51202
C6	0.47814	0.5	1.57365	C34	0.71149	0.5	1.43141
C7	0.37554	0.5	1.5843	N35	0.72753	0.5	1.46893
C8	0.38405	0.5	1.62265	C36	0.58092	0.5	1.28534
C9	0.35447	0.5	1.64197	N37	0.54432	0.5	1.26442
C10	0.3732	0.5	1.68845	H38	0.40117	0.5	1.46486
C11	0.3461	0.5	1.70809	H39	0.35313	0.5	1.49815
N12	0.30179	0.5	1.68103	H40	0.46606	0.5	1.62867
C13	0.28218	0.5	1.63571	H41	0.51311	0.5	1.59483
C14	0.30849	0.5	1.61553	H42	0.34519	0.5	1.56538
C15	0.36368	0.5	1.75725	H43	0.4144	0.5	1.64213
C16	0.23345	0.5	1.60795	H44	0.3443	0.5	1.76758
C17	0.40145	0.5	1.78788	H45	0.21879	0.5	1.57263
C18	0.42276	0.5	1.83725	H46	0.41902	0.5	1.77625
C19	0.20701	0.5	1.62425	H47	0.22116	0.5	1.65998
C20	0.15857	0.5	1.59499	H48	0.4862	0.5	1.84746
C21	0.4679	0.5	1.8634	H49	0.52481	0.5	1.92975
C22	0.48997	0.5	1.91019	H50	0.40412	0.5	1.92232
C23	0.46713	0.5	1.93158	H51	0.36493	0.5	1.84014
C24	0.42203	0.5	1.90596	H52	0.14625	0.5	1.64855
C25	0.39983	0.5	1.85899	H53	0.06568	0.5	1.60062
C26	0.13173	0.5	1.61337	H54	0.07833	0.5	1.48695
C27	0.08595	0.5	1.58617	H55	0.15827	0.5	1.53433
C28	0.06658	0.5	1.54042				

Supplementary Table 9 | Fractional atomic coordinates for the eclipsed AA-stacking unit cell of TPA-TMT.

Space group: P-6<br>***a = b = 16.8557 Å, c = 3.4595 Å***<br>***α = β = 90°, γ = 120 °***							
**Atom**	***x* (Å)**	***y* (Å)**	***z* (Å)**	**Atom**	***x* (Å)**	***y* (Å)**	***z* (Å)**
C1	-0.60526	-0.37928	0.5	N10	-0.4249	-0.72442	0.5
C2	-0.63655	-0.47634	0.5	H11	-0.69927	-0.532	0.5
C3	-0.58281	-0.51887	0.5	H12	-0.61656	-0.59292	0.5
C4	-0.48868	-0.468	0.5	H13	-0.37671	-0.3323	0.5
C5	-0.451	-0.37467	0.5	H14	-0.45594	-0.26735	0.5
C6	-0.50607	-0.33364	0.5	H15	-0.35413	-0.45978	0.5
C7	-0.42722	-0.50767	0.5	H16	-0.52724	-0.64856	0.5
C8	-0.45557	-0.59765	0.5	N17	-0.66667	-0.33333	0.5
C9	-0.39134	-0.63283	0.5				

Supplementary Table 10 | Fractional atomic coordinates for the eclipsed AA-stacking unit cell of TFPT-TMT.

Space group: P-6<br>***a = b = 18.7517 Å, c = 3.4602 Å***<br>***α = β = 90°, γ = 120°***							
**Atom**	***x* (Å)**	***y* (Å)**	***z* (Å)**	**Atom**	***x* (Å)**	***y* (Å)**	***z* (Å)**
C1	-0.62999	-0.38022	0.5	C10	-0.36431	-0.61501	0.5
C2	-0.58997	-0.4314	0.5	N11	-0.2819	-0.58428	0.5
C3	-0.63758	-0.51777	0.5	N12	-0.58373	-0.29693	0.5
C4	-0.59984	-0.56587	0.5	H13	-0.70417	-0.54812	0.5
C5	-0.51371	-0.52912	0.5	H14	-0.63825	-0.63227	0.5
C6	-0.46593	-0.44252	0.5	H15	-0.39957	-0.41093	0.5
C7	-0.50372	-0.39441	0.5	H16	-0.4654	-0.328	0.5
C8	-0.47758	-0.58387	0.5	H17	-0.52037	-0.64964	0.5
C9	-0.39657	-0.5578	0.5	H18	-0.35121	-0.49322	0.5

Supplementary Table 11 | Fractional atomic coordinates for the eclipsed AA-stacking unit cell of TFPB-TMT.

Space group: P-6 ***a = b = 19.0061 Å, c = 3.4580 Å*** ***α = β = 90°, γ = 120°***							
**Atom**	***x* (Å)**	***y* (Å)**	***z* (Å)**	**Atom**	***x* (Å)**	***y* (Å)**	***z* (Å)**
C1	-0.56619	-0.4122	0.5	C11	-0.61806	-0.37149	0.5
C2	-0.60144	-0.49796	0.5	C12	-0.58245	-0.28616	0.5
C3	-0.55387	-0.53511	0.5	H13	-0.66585	-0.53899	0.5
C4	-0.46882	-0.48845	0.5	H14	-0.58495	-0.60058	0.5
C5	-0.43287	-0.40389	0.5	H15	-0.36733	-0.36614	0.5
C6	-0.48006	-0.3664	0.5	H16	-0.44636	-0.3012	0.5
C7	-0.41486	-0.5242	0.5	H17	-0.34996	-0.48206	0.5
C8	-0.44064	-0.60413	0.5	H18	-0.50436	-0.64885	0.5
C9	-0.38425	-0.63603	0.5	H19	-0.51834	-0.25025	0.5
N10	-0.41463	-0.71735	0.5				

Supplementary Table 12 | Fractional atomic coordinates for the eclipsed AA-stacking unit cell of DAFB-TMT.

Space group: PM ***a = 26.4646 Å, b = 3.6192 Å, c = 26.1299 Å*** ***α = γ = 90°, β = 120°***							
**Atom**	***x* (Å)**	***y* (Å)**	***z* (Å)**	**Atom**	***x* (Å)**	***y* (Å)**	***z* (Å)**
C1	0.40275	0.5	0.55218	C47	0.88455	0.5	0.6793
C2	0.36596	0.5	0.58028	C48	0.33163	0.5	0.08768
C3	0.30524	0.5	0.54636	C49	0.30276	0.5	0.02585
C4	0.27056	0.5	0.57262	C50	0.33451	0.5	-0.00361
C5	0.29599	0.5	0.63339	C51	0.39521	0.5	0.02945
C6	0.35647	0.5	0.66756	C52	0.42395	0.5	0.09111
C7	0.39112	0.5	0.64116	C53	1.04322	0.5	0.72432
C8	0.25833	0.5	0.65979	C54	0.30652	0.5	-0.06846
C9	0.27351	0.5	0.71181	C55	0.3236	0.5	0.83424
C10	0.23612	0.5	0.7395	N56	0.37204	0.5	0.86055
C11	0.26293	0.5	0.80119	C57	0.14668	0.5	0.64459
C12	0.2291	0.5	0.82914	N58	0.12377	0.5	0.59508
N13	0.17013	0.5	0.79529	H59	0.2846	0.5	0.49966
C14	0.14221	0.5	0.73534	H60	0.22389	0.5	0.5458
C15	0.17517	0.5	0.70655	H61	0.37665	0.5	0.71434

Supplementary Table 13 | Fractional atomic coordinates for the eclipsed AA-stacking unit cell of BDA-TMT.

Space group: P6/M ***a = b = 30.3917 Å, c = 3.4663 Å*** ***α = β = 90°, γ = 120°***							
**Atom**	***x* (Å)**	***y* (Å)**	***z* (Å)**	**Atom**	***x* (Å)**	***y* (Å)**	***z* (Å)**
C1	1.5122	1.48346	0.5	C9	1.64835	1.36587	0.5
C2	1.48263	1.42977	0.5	N10	1.69906	1.38404	0.5
C3	1.50521	1.39946	0.5	H11	1.44181	1.40979	0.5
C4	1.55806	1.42115	0.5	H12	1.481	1.35855	0.5
C5	1.58806	1.47432	0.5	H13	1.62898	1.49316	0.5
C6	1.56563	1.50481	0.5	H14	1.59142	1.54511	0.5
C7	1.5795	1.38662	0.5	H15	1.5526	1.34615	0.5
C8	1.62931	1.40189	0.5	H16	1.65778	1.44157	0.5

Supplementary Table 14 | Fractional atomic coordinates for the eclipsed AA-stacking unit cell of TDA-TMT.

Space group: P6/M ***a = b = 37.9922 Å, c = 3.4659 Å*** ***α = β = 90°, γ = 120°***							
**Atom**	***x* (Å)**	***y* (Å)**	***z* (Å)**	**Atom**	***x* (Å)**	***y* (Å)**	***z* (Å)**
C1	1.51922	0.47552	0.5	C12	1.5421	0.51838	0.5
C2	1.53946	0.44972	0.5	C13	1.52346	0.54212	0.5
C3	1.51644	0.40673	0.5	H14	1.48378	0.39025	0.5
C4	1.53517	0.3831	0.5	H15	1.51627	0.35031	0.5
C5	1.57751	0.40112	0.5	H16	1.63365	0.45927	0.5
C6	1.6009	0.4437	0.5	H17	1.60248	0.4998	0.5
C7	1.58229	0.46748	0.5	H18	1.57431	0.34165	0.5
C8	1.59539	0.37412	0.5	H19	1.65775	0.41889	0.5
C9	1.63538	0.38701	0.5	H20	1.57476	0.5348	0.5
C10	1.65134	0.3588	0.5	H21	1.54363	0.57445	0.5
N11	1.69202	0.374	0.5				

Supplementary Table 15 | Fractional atomic coordinates for the eclipsed AA-stacking unit cell of BTTA-TMT.

Space group: P-6 ***a = b = 23.2815 Å, c = 3.4855 Å*** ***α = β = 90°, γ = 120°***							
**Atom**	***x* (Å)**	***y* (Å)**	***z* (Å)**	**Atom**	***x* (Å)**	***y* (Å)**	***z* (Å)**
C1	-0.736	-0.36453	0.5	C12	-0.38614	-0.58055	0.5
C2	-0.70478	-0.40263	0.5	C13	-0.35918	-0.62582	0.5
C3	-0.60343	-0.41061	0.5	N14	-0.29267	-0.60018	0.5
C4	-0.5566	-0.13316	0.5	H15	-0.73428	-0.45628	0.5
C5	-0.54446	-0.48249	0.5	H16	-0.636	-0.57614	0.5
C6	-0.58236	-0.55168	0.5	H17	-0.58151	-0.64325	0.5
C7	-0.55117	-0.58971	0.5	H18	-0.39042	-0.46315	0.5
C8	-0.48162	-0.55918	0.5	H19	-0.44564	-0.39778	0.5
C9	-0.44388	-0.48927	0.5	H20	-0.48545	-0.65554	0.5
C10	-0.47527	-0.45139	0.5	H21	-0.35017	-0.52837	0.5
C11	-0.45157	-0.60244	0.5				

Supplementary Table 16 | Fractional atomic coordinates for the eclipsed AA-stacking unit cell of EDA-TMT.

Space group: P6/M ***a = b = 34.6739 Å, c = 3.4826 Å*** ***α =γ= 90°, β = 120°***							
**Atom**	***x* (Å)**	***y* (Å)**	***z* (Å)**	**Atom**	***x* (Å)**	***y* (Å)**	***z* (Å)**
C1	1.52938	0.46163	0.5	N10	1.69467	0.37785	0.5
C2	1.50317	0.41523	0.5	C11	1.49133	0.51133	0.5
C3	1.52329	0.38893	0.5	H12	1.4672	0.39945	0.5
C4	1.56991	0.4086	0.5	H13	1.50233	0.35305	0.5
C5	1.59603	0.45548	0.5	H14	1.63191	0.4724	0.5
C6	1.57578	0.48168	0.5	H15	1.59627	0.51763	0.5
C7	1.58919	0.37876	0.5	H16	1.56589	0.34322	0.5
C8	1.63294	0.39262	0.5	H17	1.65762	0.4275	0.5
C9	1.65014	0.36147	0.5				

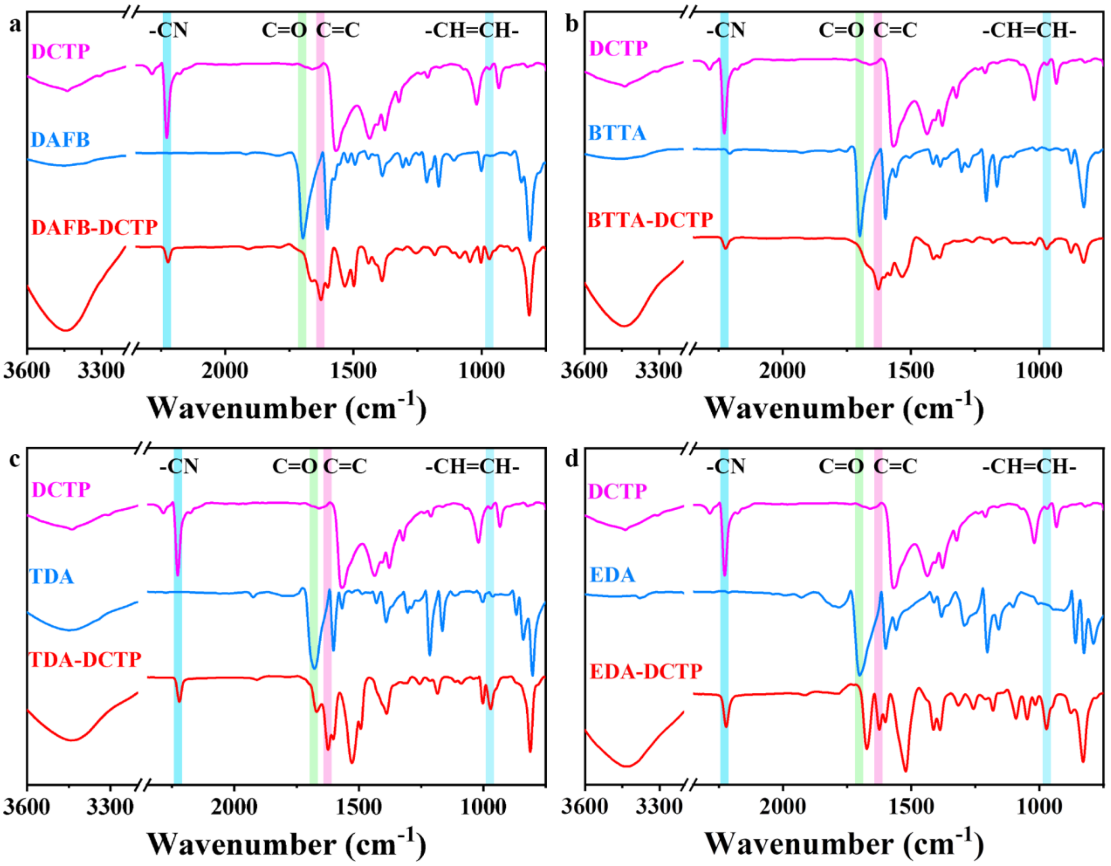

Supplementary Fig. 11 | FT-IR spectra of DAFB-DCTP (a), BTTA-DCTP (b), TDA-DCTP (c), and EDA-DCTP (d). The core of structure characterizations was to prove the successful formation of olefin linkages in the frameworks. In the FT-IR spectra of DAFB-DCTP, BTTA-DCTP, TDA-DCTP and EDA-DCTP, the stretching vibration peak of C=O (*ca.* 1698 cm^{-1}) completely disappeared and the two new peaks of the C=C in *trans*-configuration (at *ca.* 1625, and *ca.* 968 cm^{-1}) were found,[1] confirming that these COFs were completely condensed and the vinylene linkages were successfully formed.

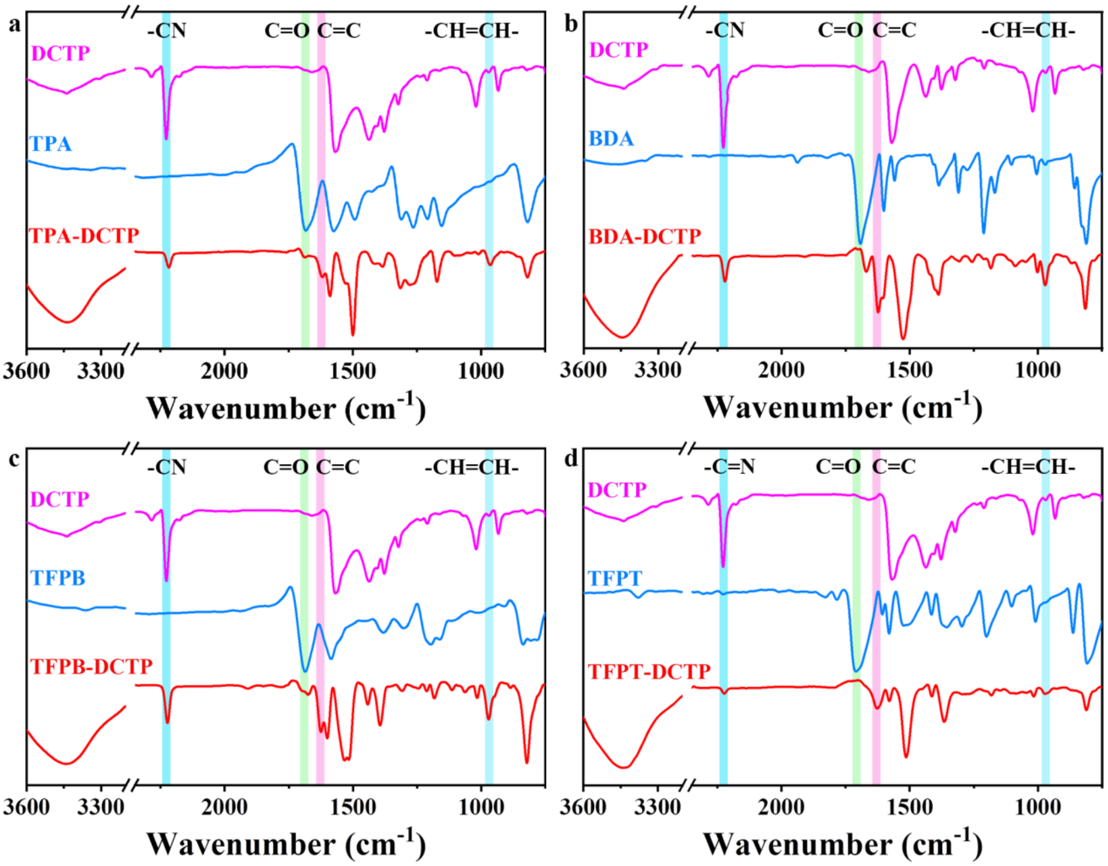

Supplementary Fig. 12 | FT-IR spectra of TPA-DCTP (a), BDA-DCTP (b), TFPB-DCTP (c), and TFPT-DCTP (d). The core of structure characterizations was to prove the successful formation of olefin linkages in the frameworks. In the FT-IR spectra of TPA-DCTP, BDA-DCTP, TFPB-DCTP and TFPT-DCTP, the stretching vibration peak of C=O (*ca.* 1682 cm^{-1}) completely disappeared and the two new peaks of the C=C in *trans*-configuration (at *ca.* 1625, and *ca.* 967 cm^{-1}) were found,[1] confirming that these COFs were completely condensed and the vinylene linkages were successfully formed.

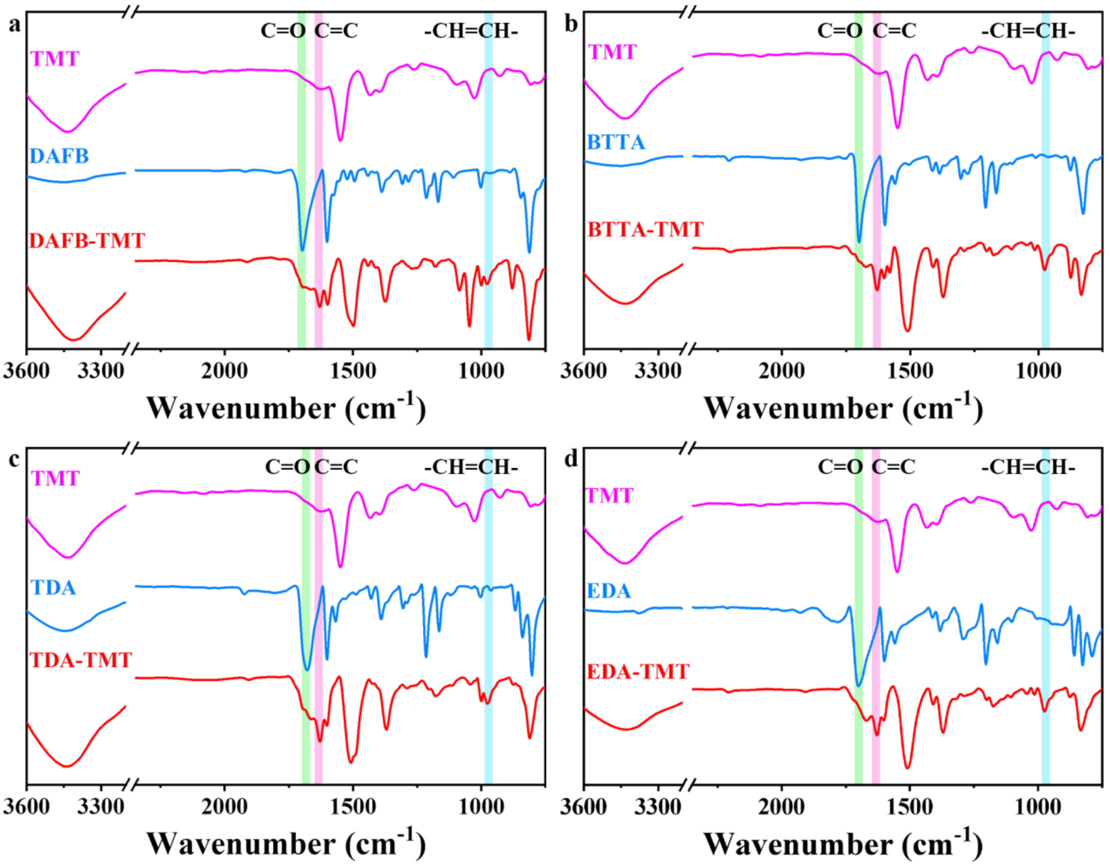

Supplementary Fig. 13 | FT-IR spectra of DAFB-TMT (a), BTTA-TMT (b), TDA-TMT (c), and EDA-TMT (d). The core of structure characterizations was to prove the successful formation of olefin linkages in the frameworks. In the FT-IR spectra of DAFB-TMT, BTTA-TMT, TDA-TMT and EDA-TMT, the stretching vibration peak of C=O (*ca.* 1698 cm^{-1}) completely disappeared and the two new peaks of the C=C in *trans*-configuration (at *ca.* 1630, and *ca.* 986 cm^{-1}) were found,[1] confirming that these COFs were completely condensed and the vinylene linkages were successfully formed.

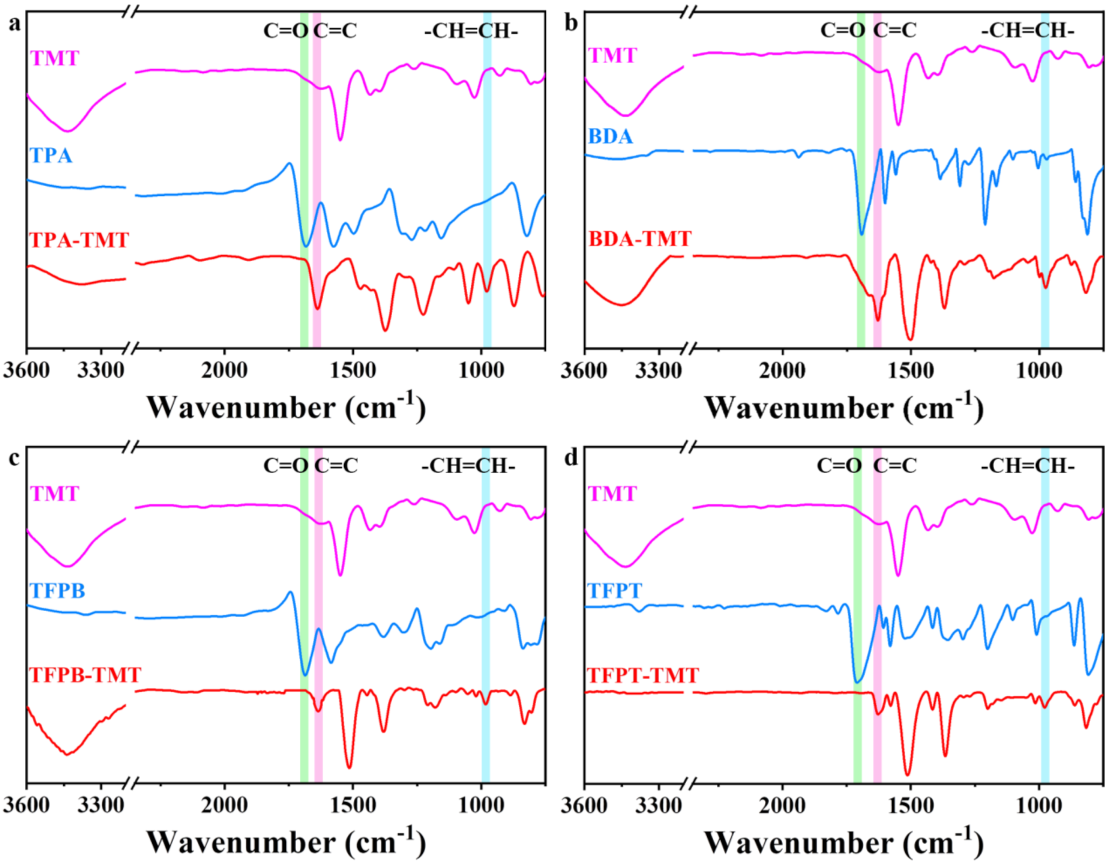

Supplementary Fig. 14 | FT-IR spectra of TPA-TMT (a), BDA-TMT (b), TFPB-TMT (c), and TFPT-TMT (d). The core of structure characterizations was to prove the successful formation of olefin linkages in the frameworks. In the FT-IR spectra of TPA-TMT, BDA-TMT, TFPB-TMT and TFPT-TMT, the stretching vibration peak of C=O (*ca.* 1682 cm^{-1}) completely disappeared and the two new peaks of the C=C in *trans*-configuration (at *ca.* 1639, and *ca.* 982 cm^{-1}) were found,[1] confirming that these COFs were completely condensed and the vinylene linkages were successfully formed.

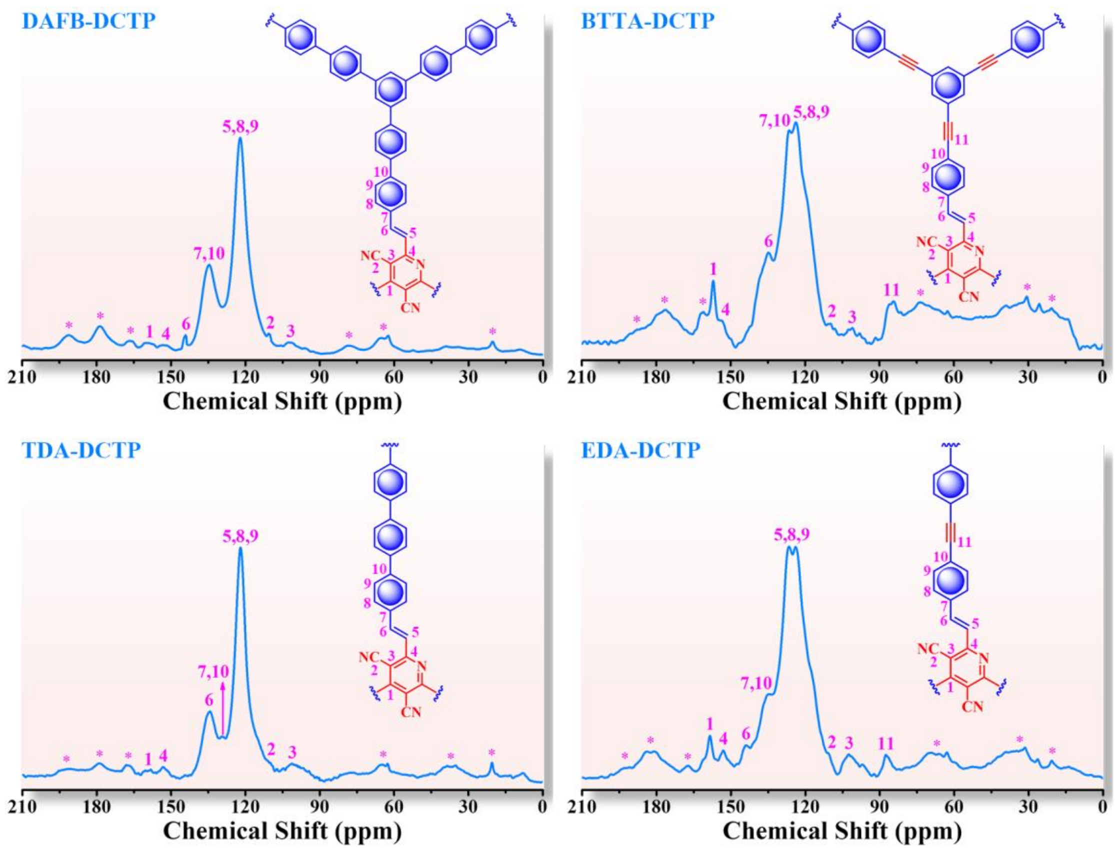

Supplementary Fig. 15 | Solid-state ^{13}C CP/MAS NMR spectra of DAFB-DCTP, BTTA-DCTP, TDA-DCTP and EDA-DCTP. The ^{13}C CP/MAS NMR spectra of DAFB-DCTP and TDA-DCTP further confirmed the successful formation of the vinylene linkages, supported by the signals located at *ca*. 135 and *ca*. 110 ppm ascribed to the carbon atoms in the vinylene linkages (-C=C-) and cyano (-CN) moieties. The ^{13}C cross-polarization/magic-angle-spinning (CP/MAS) NMR spectra of BTTA-DCTP and EDA-DCTP further confirmed the successful formation of the vinylene linkages (-C=C-), supported by the signals located at *ca*. 135, *ca*. 110 and *ca*. 87 ppm ascribed to the carbon atoms in the vinylene linkages (-C=C-), cyano (-CN) and acetylene (-C≡C-) moieties. Spinning side bands are marked with asterisk (*).

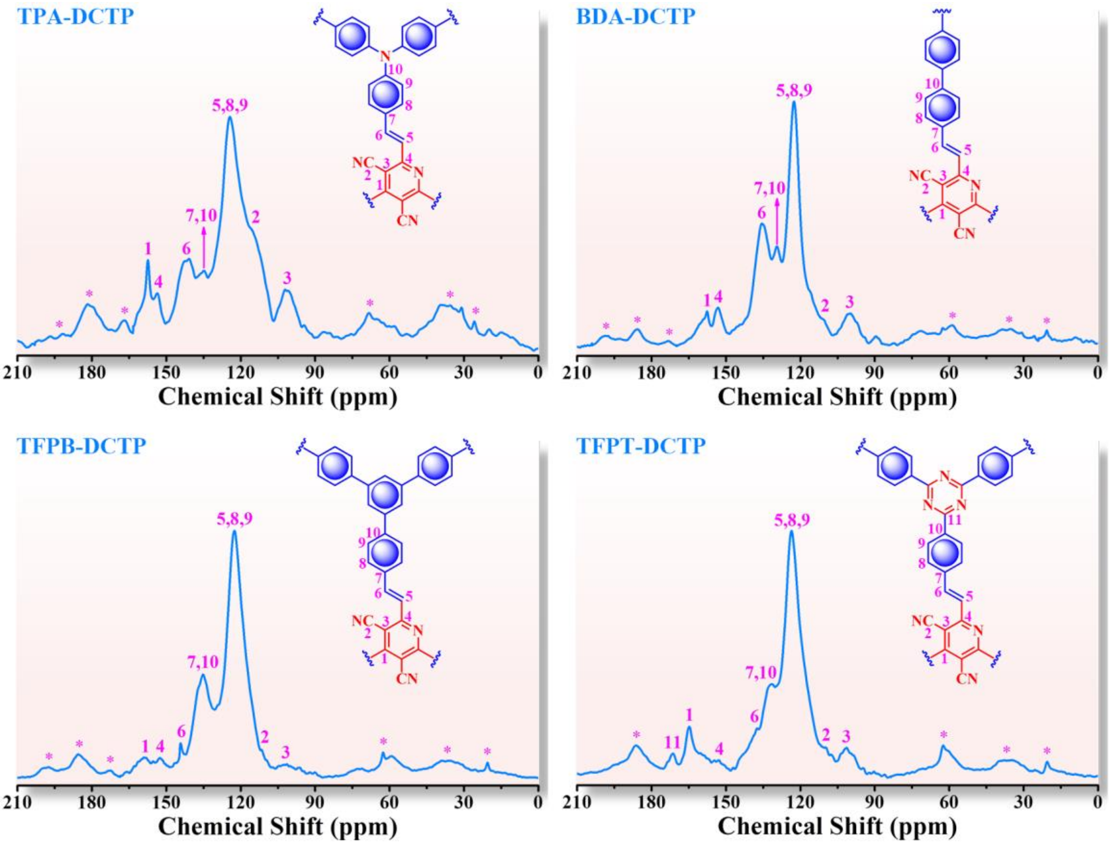

Supplementary Fig. 16 | Solid-state ^{13}C CP/MAS NMR spectra of TPA-DCTP, BDA-DCTP, TFPB-DCTP and TFPT-DCTP. The ^{13}C CP/MAS NMR spectra of TPA-DCTP, BDA-DCTP and TFPB-DCTP further confirmed the successful formation of the vinylene linkages, supported by the signals located at *ca.* 135 and *ca.* 110 ppm ascribed to the carbon atoms in the vinylene linkages (-C=C-) and cyano (-CN) moieties. The ^{13}C cross-polarization/magic-angle-spinning (CP/MAS) NMR spectrum of TFPT-DCTP further confirmed the successful formation of the vinylene linkages (-C=C-), supported by the signals located at *ca.* 135, *ca.* 110 and *ca.* 171 ppm ascribed to the carbon atoms in the vinylene linkages (-C=C-), cyano (-CN) and triazine moieties. Spinning side bands are marked with asterisk (*).

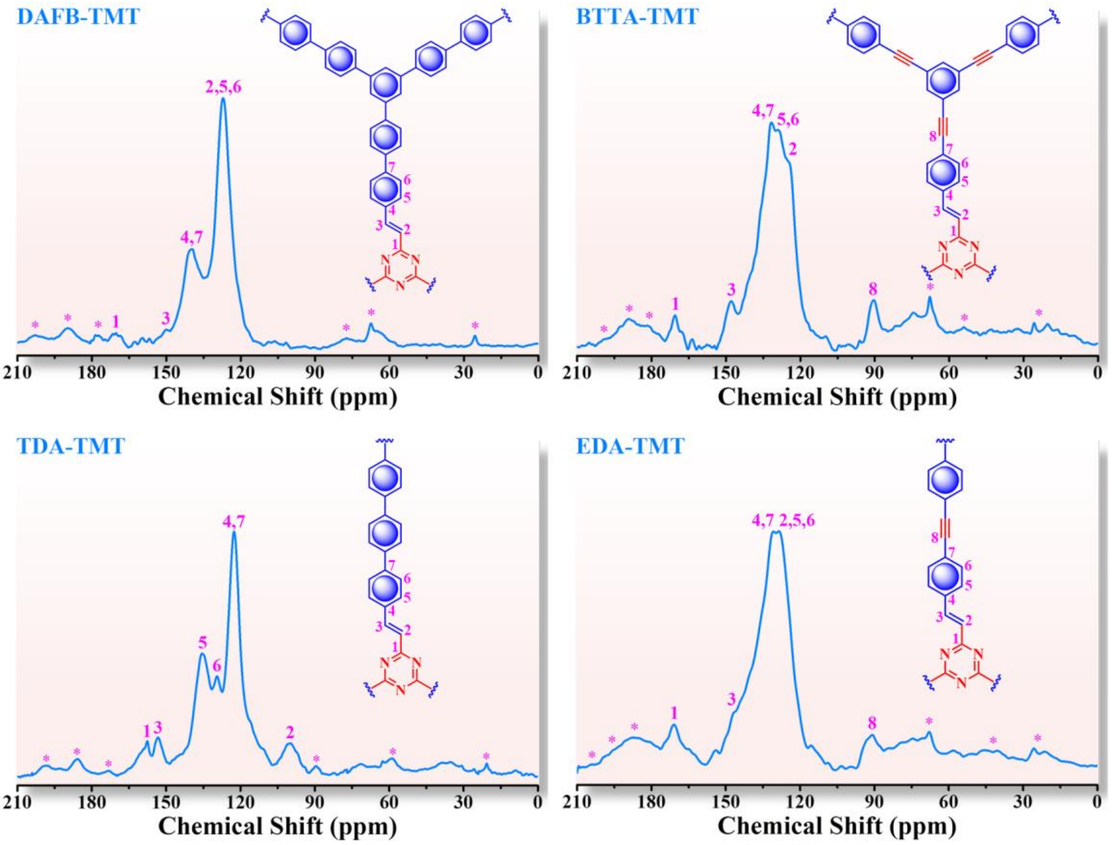

Supplementary Fig. 17 | Solid-state ^{13}C CP/MAS NMR spectra of DAFB-TMT, BTTA-TMT, TDA-TMT and EDA-TMT. The ^{13}C CP/MAS NMR spectra of DAFB-TMT and TDA-TMT further confirmed the successful formation of the vinylene linkages, supported by the signals located at *ca.* 150 and *ca.* 171 ppm ascribed to the carbon atoms in the vinylene linkages (-C=C-) and triazine moieties. The ^{13}C CP/MAS NMR spectra of BTTA-TMT and EDA-DCTP further confirmed the successful formation of the vinylene linkages (-C=C-), supported by the signals located at *ca.* 150, *ca.* 171 and *ca.* 87 ppm ascribed to the carbon atoms in the vinylene linkages (-C=C-), triazine and acetylene (-C≡C-) moieties. Spinning side bands are marked with asterisk (*).

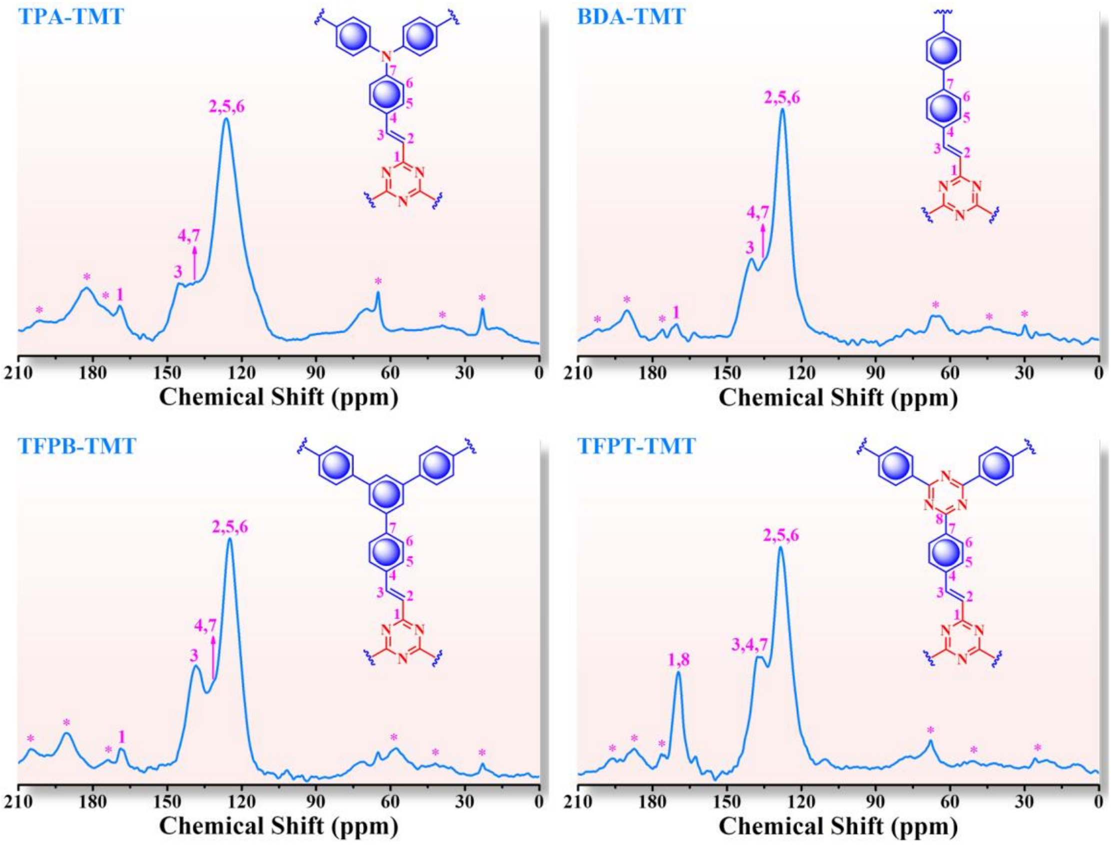

Supplementary Fig. 18 | Solid-state ^{13}C CP/MAS NMR spectra of TPA-TMT, BDA-TMT, TFPB-TMT and TFPT-TMT. The ^{13}C CP/MAS NMR spectra of TPA-TMT, BDA-TMT, TFPB-TMT and TFPT-TMT further confirmed the successful formation of the vinylene linkages, supported by the signals located at *ca.* 140 and *ca.* 171 ppm ascribed to the carbon atoms in the vinylene linkages (-C=C-) and triazine moieties. Spinning side bands are marked with asterisk (*).

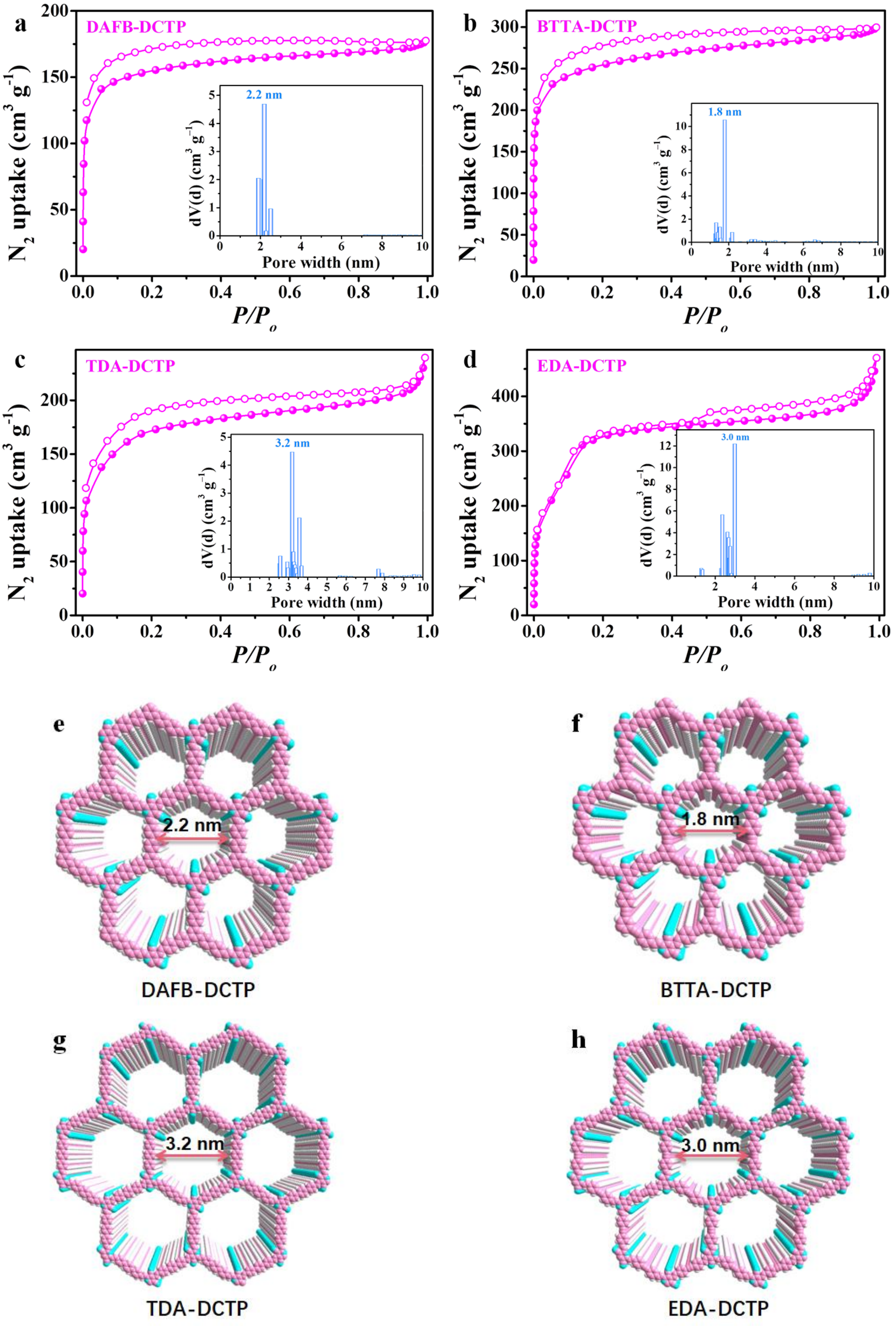

Supplementary Fig. 19 | N_2 adsorption–desorption isotherms of DAFB-DCTP (a), BTTA-DCTP (b), TDA-DCTP (c), and EDA-DCTP (d). Insets are the pore size

distributions calculated from nonlocal density functional theory (NLDFT) model. The porosities of DAFB-DCTP, BTTA-DCTP, TDA-DCTP, and EDA-DCTP were determined by N_2 adsorption-desorption isotherms at 77 K. By using the Brunauer–Emmett–Teller (BET) model, the surface areas of DAFB-DCTP, BTTA-DCTP, TDA-DCTP, and EDA-DCTP were calculated to be 578.1, 944.4, 630.5 and 1334.2 $m^2 g^{-1}$, respectively. Based on the NLDFT model, the pore size distributions of DAFB-DCTP, BTTA-DCTP, TDA-DCTP, and EDA-DCTP are centered at 2.2, 1.8, 3.2 and 3.0 nm, respectively, which is in good agreement with the AA-stacking model (e-h).

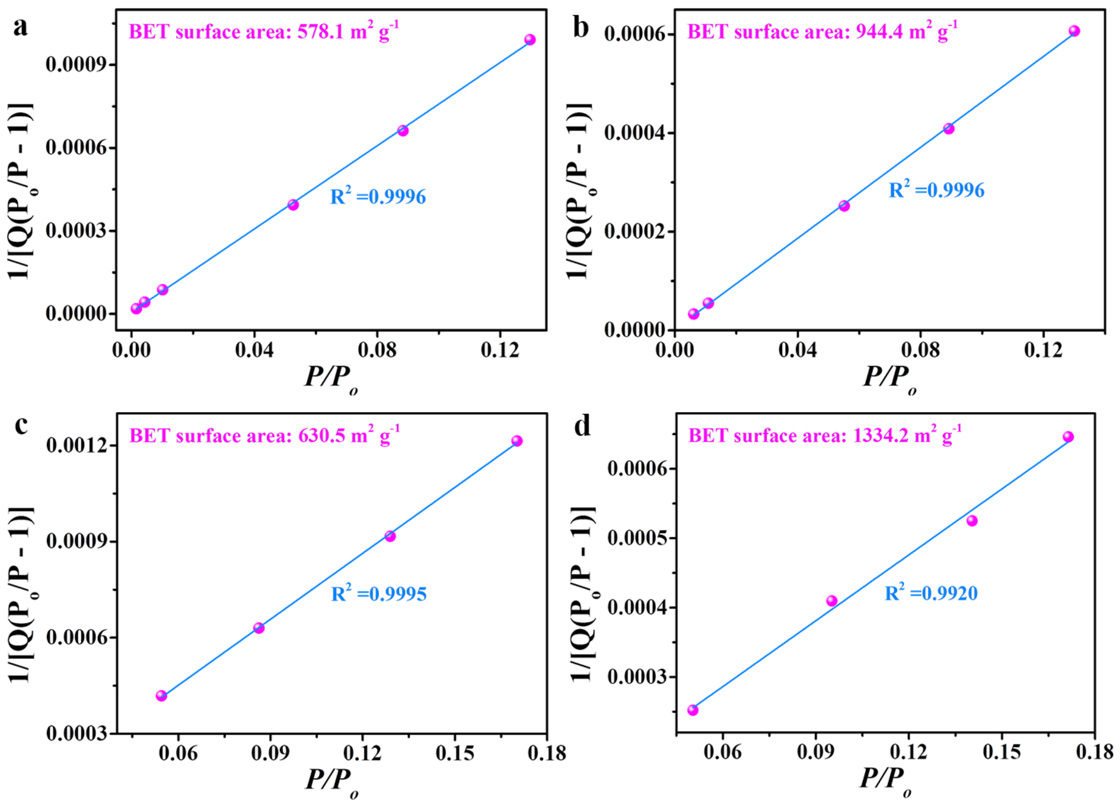

Supplementary Fig. 20 | BET surface area plot of DAFB-DCTP (a), BTTA-DCTP (b), TDA-DCTP (c), and EDA-DCTP (d) calculated from the isotherm.

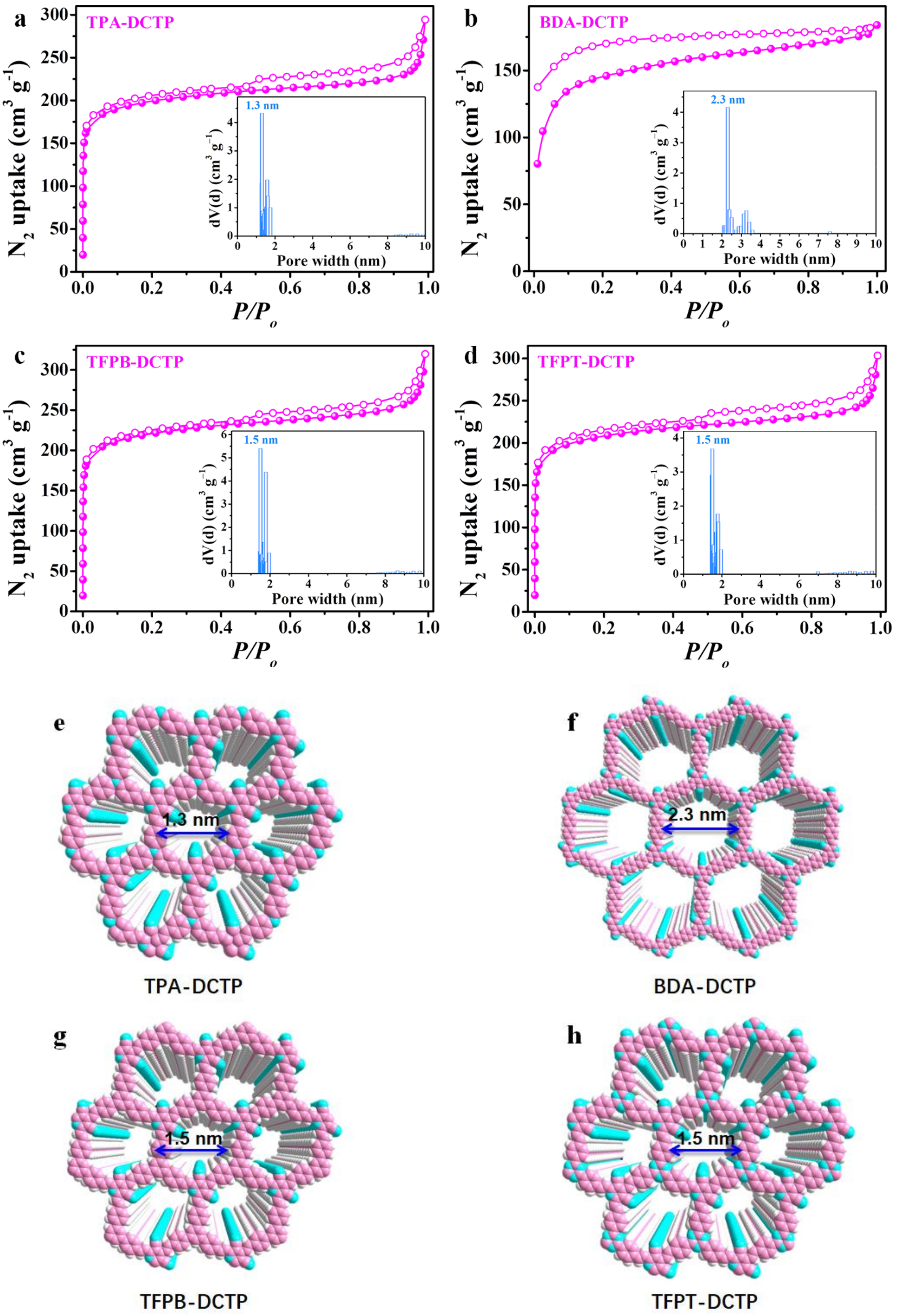

Supplementary Fig. 21 | N_2 adsorption–desorption isotherms of TPA-DCTP (a), BDA-DCTP (b), TFPB-DCTP (c), and TFPT-DCTP (d). Insets are the pore size

distributions calculated from NLDFT model. The porosities of TPA-DCTP, BDA-DCTP, TFPB-DCTP and TFPT-DCTP were determined by N_2 adsorption-desorption isotherms at 77 K. By using the BET model, the surface areas of TPA-DCTP, BDA-DCTP, TFPB-DCTP and TFPT-DCTP were calculated to be 756.5, 503.8, 822.0 and 733.8 m^2 g^{-1}, respectively. Based on the NLDFT model, the pore size distributions of TPA-DCTP, BDA-DCTP, TFPB-DCTP and TFPT-DCTP are centered at 1.3, 2.3, 1.5 and 1.5 nm, respectively, which is in good agreement with the AA-stacking model (e-h).

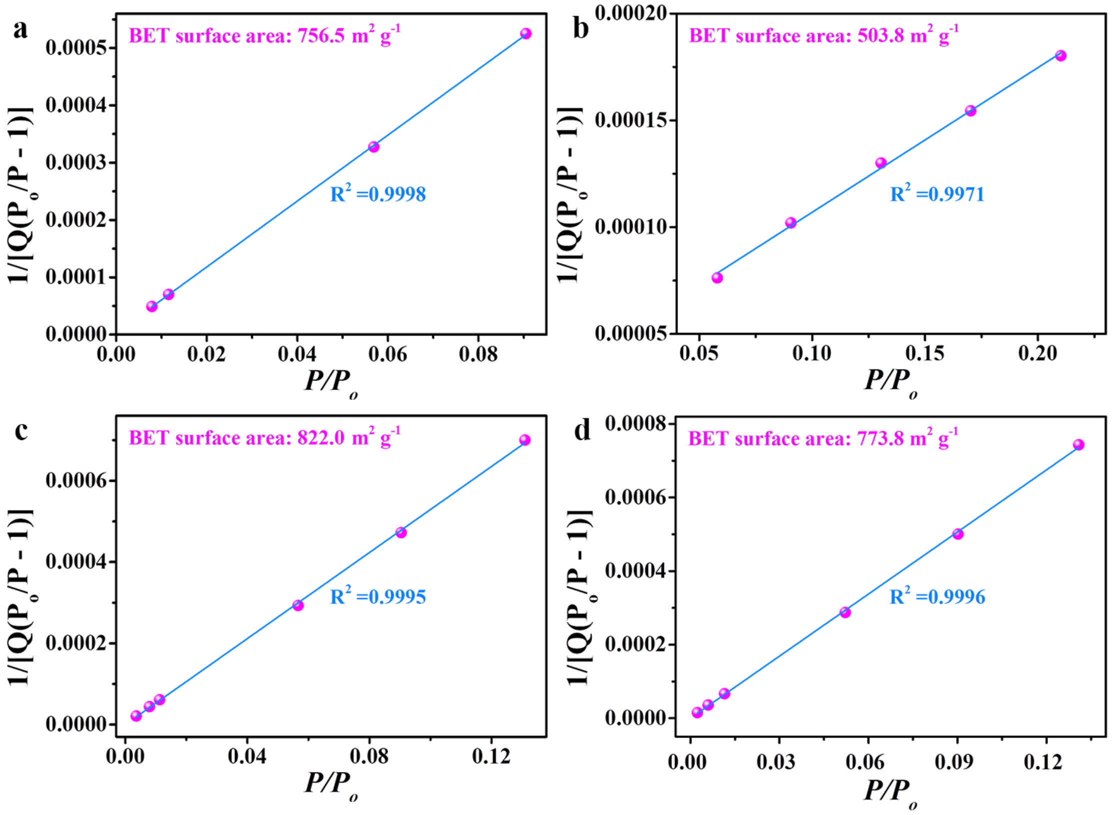

Supplementary Fig. 22 | BET surface area plot of TPA-DCTP (a), BDA-DCTP (b), TFPB-DCTP (c), and TFPT-DCTP (d) calculated from the isotherm.

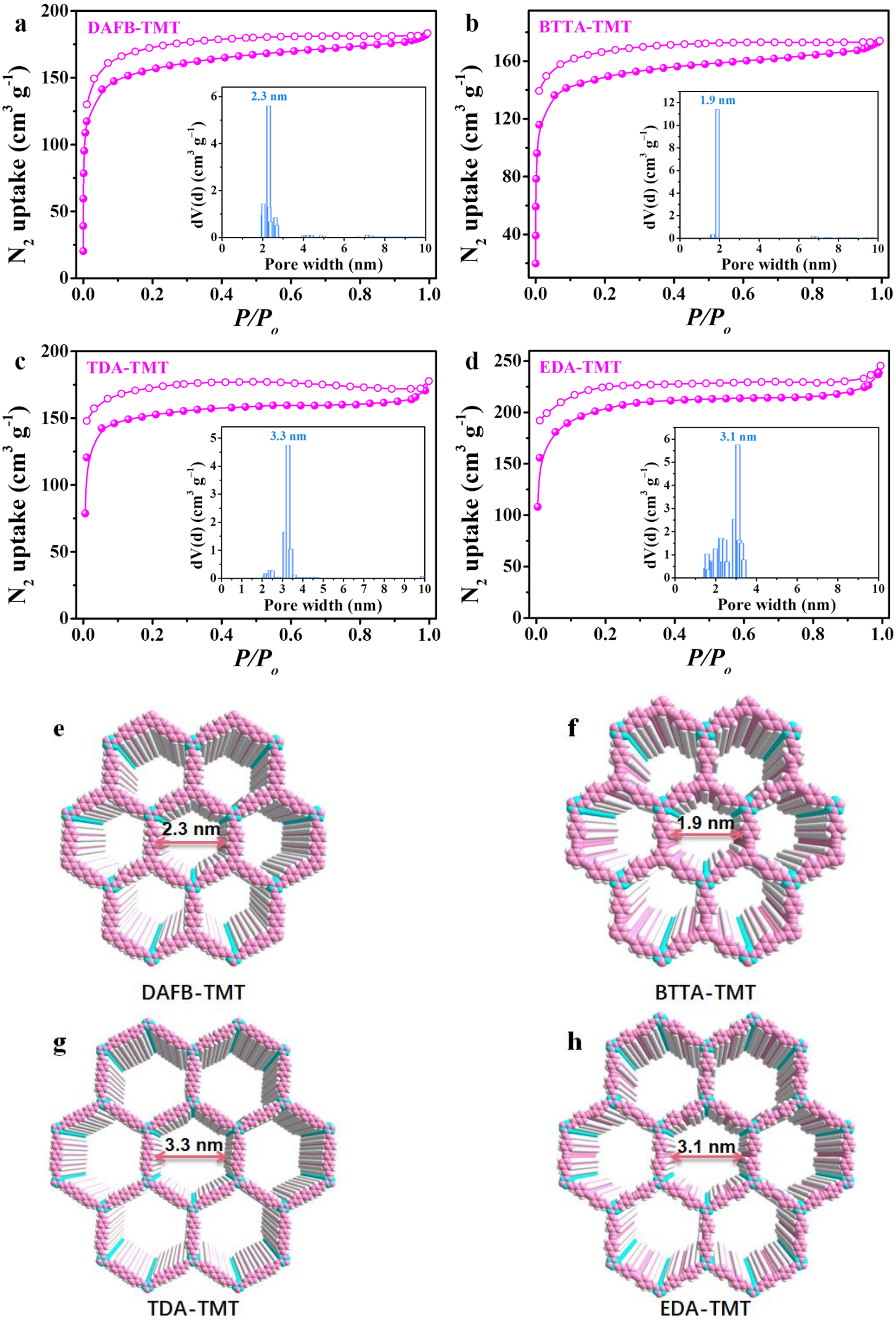

Supplementary Fig. 23 | N_2 adsorption–desorption isotherms of DAFB-TMT (a), BTTA-TMT (b), TDA-TMT (c), and EDA-TMT (d). Insets are the pore size distributions calculated from nonlocal NLDFT model. The porosities of DAFB-TMT,

BTTA-TMT, TDA-TMT and EDA-TMT were determined by N_2 adsorption-desorption isotherms at 77 K. By using the BET model, the surface areas of DAFB-TMT, BTTA-TMT, TDA-TMT and EDA-TMT were calculated to be 583.4, 555.9, 501.7 and 612.7 m^2 g^{-1}, respectively. Based on the NLDFT model, the pore size distributions of DAFB-TMT, BTTA-TMT, TDA-TMT and EDA-TMT are centered at 2.3, 1.9, 3.3 and 3.1 nm, respectively, which is in good agreement with the AA-stacking model (e-h).

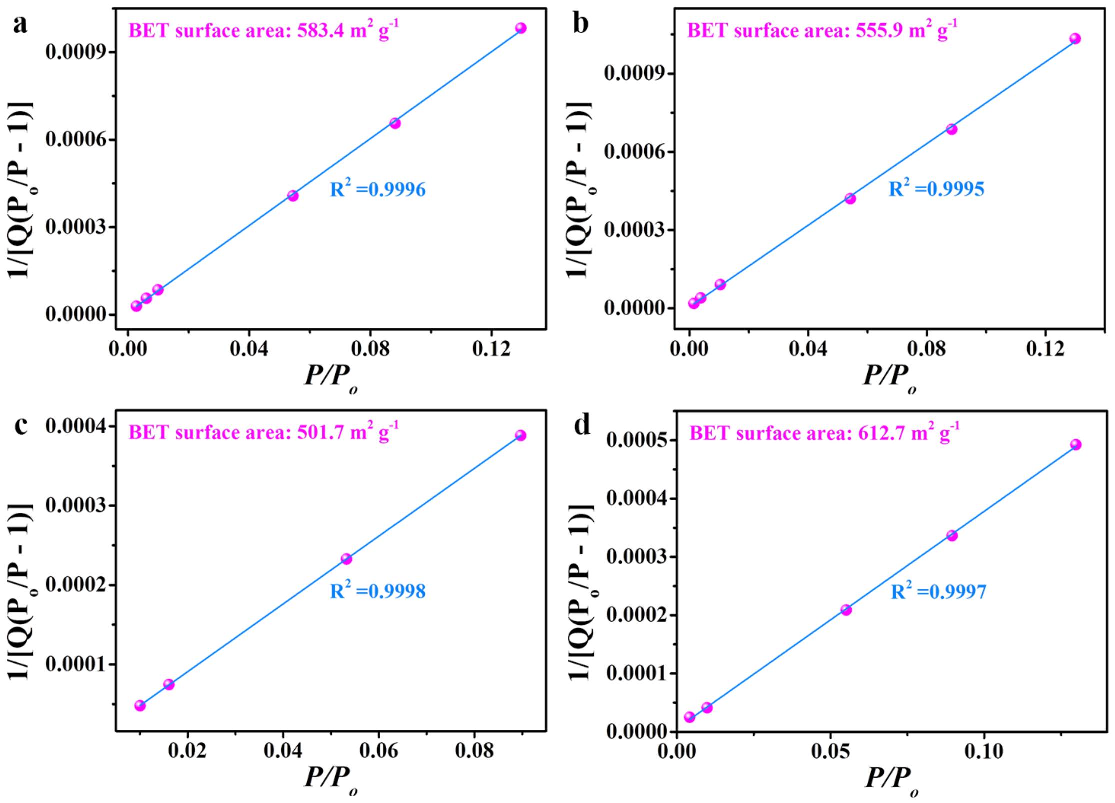

Supplementary Fig. 24 | BET surface area plot of DAFB-TMT (a), BTTA-TMT (b), TDA-TMT (c), and EDA-TMT (d) calculated from the isotherm.

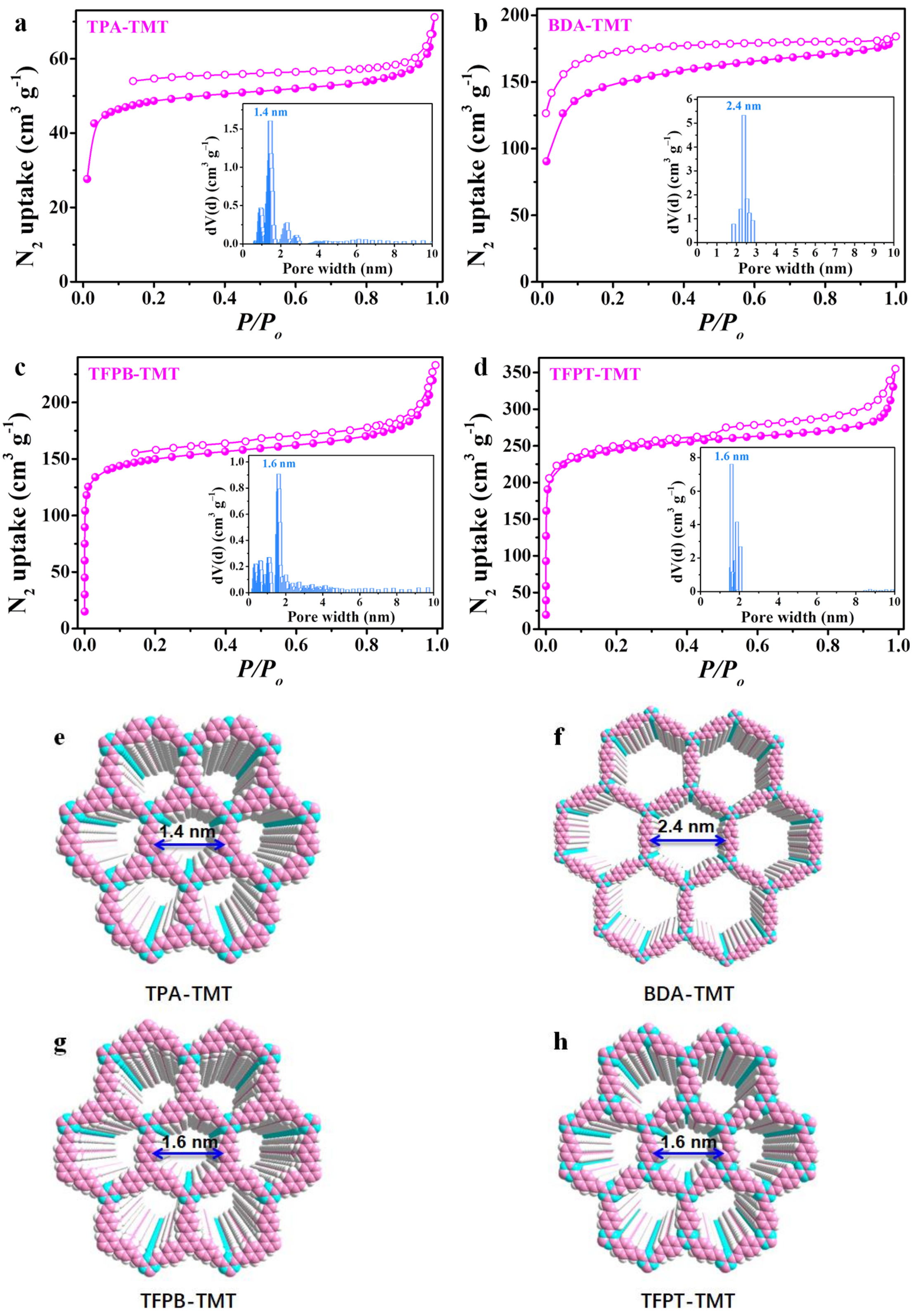

Supplementary Fig. 25 | N_2 adsorption–desorption isotherms of TPA-TMT (a), BDA-TMT (b), TFPB-TMT (c), and TFPT-TMT (d). Insets are the pore size distributions calculated from NLDFT model. The porosities of TPA-TMT, BDA-TMT, TFPB-TMT, and TFPT-TMT were determined by N_2 adsorption-desorption isotherms

at 77 K. By using the Brunauer–Emmett–Teller (BET) model, the surface areas of TPA-TMT, BDA-TMT, TFPB-TMT and TFPT-TMT were calculated to be 492.4, 524.0, 563.3 and 928.2 $m^2 g^{-1}$, respectively. Based on the NLDFT model, the pore size distributions of TPA-TMT, BDA-TMT, TFPB-TMT and TFPT-TMT are centered at 1.4, 2.4, 1.6 and 1.6 nm, respectively, which is in good agreement with the AA-stacking model (e-h).

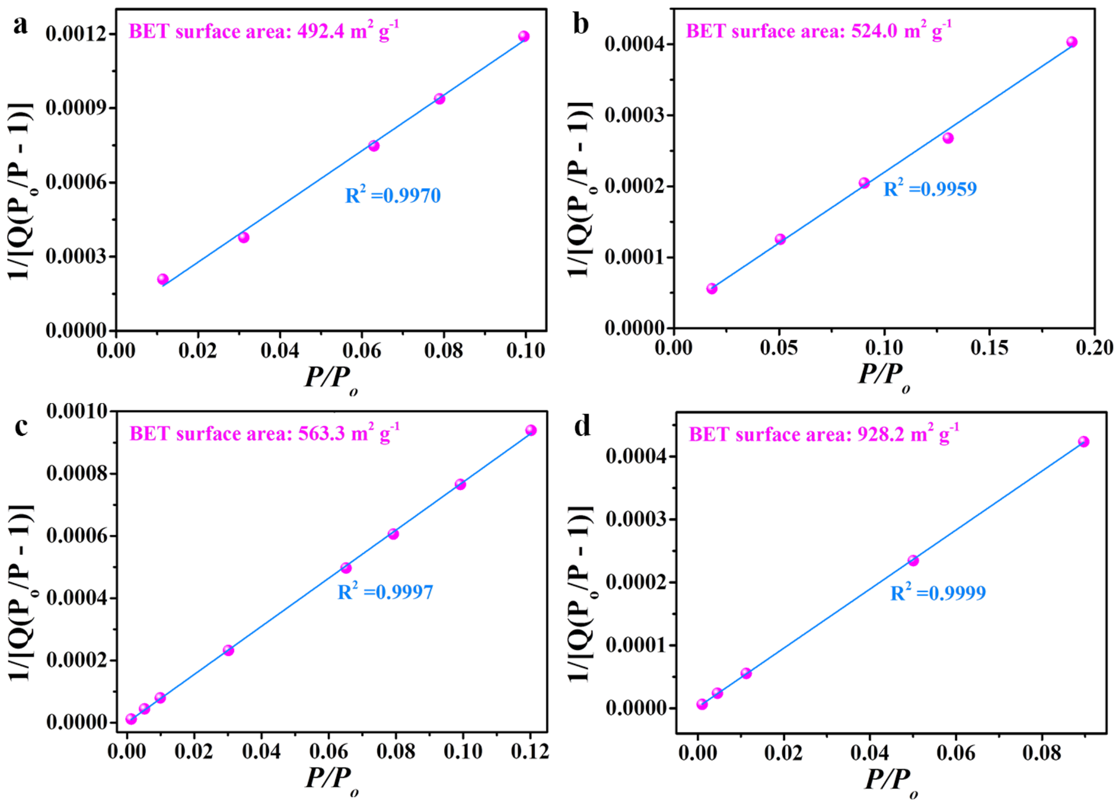

Supplementary Fig. 26 | BET surface area plot of TPA-TMT (a), BDA-TMT (b), TFPB-TMT (c), and TFPT-TMT (d) calculated from the isotherm.

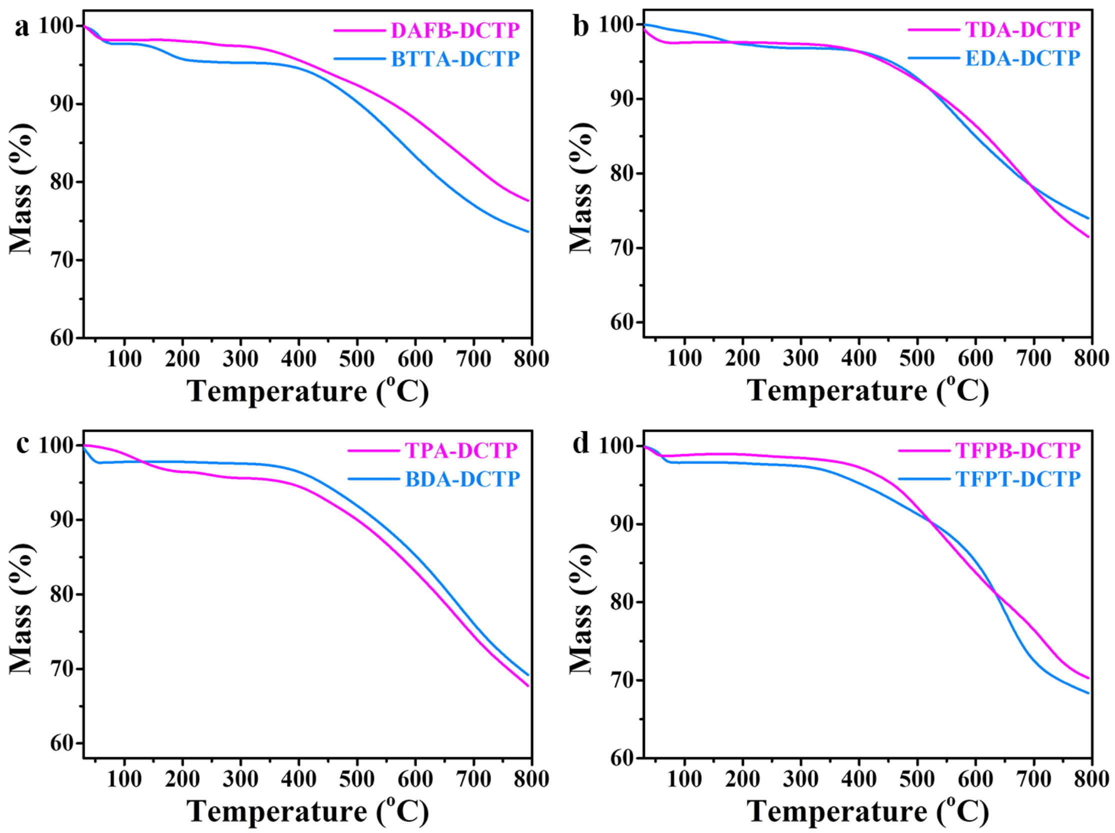

Supplementary Fig. 27 | TGA curves of DAFB-DCTP and BTTA-DCTP (a). TGA curves of TDA-DCTP and EDA-DCTP (b). TGA curves of TPA-DCTP and BDA-DCTP (c). TGA curves of TFPB-DCTP and TFPT-DCTP (d). All the TGA data indicate that olefin-linked COFs are thermally stable up to *ca*. 300 ℃.

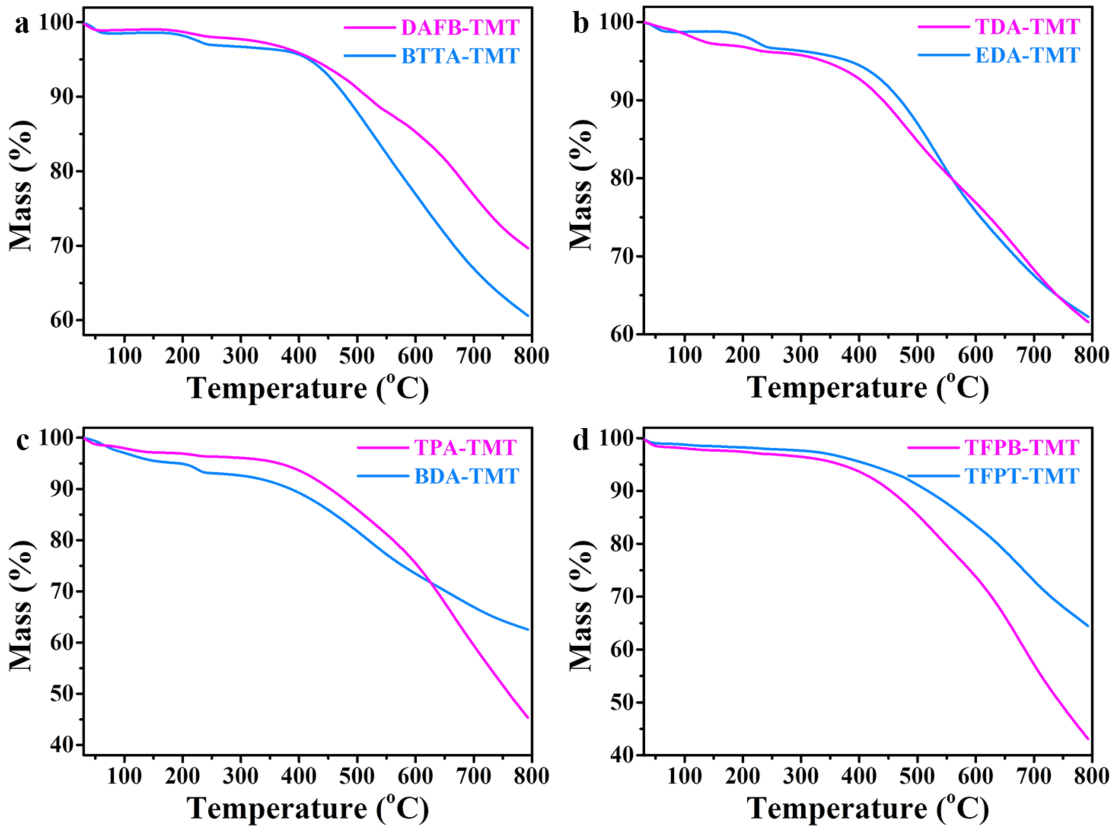

Supplementary Fig. 28 | TGA curves of DAFB-TMT and BTTA-TMT (a). TGA curves of TDA-TMT and EDA-TMT (b). TGA curves of TPA-TMT and BDA-TMT (c). TGA curves of TFPB-TMT and TFPT-TMT (d). All the TGA data indicate that olefin-linked COFs are thermally stable up to *ca*. 400 ℃.

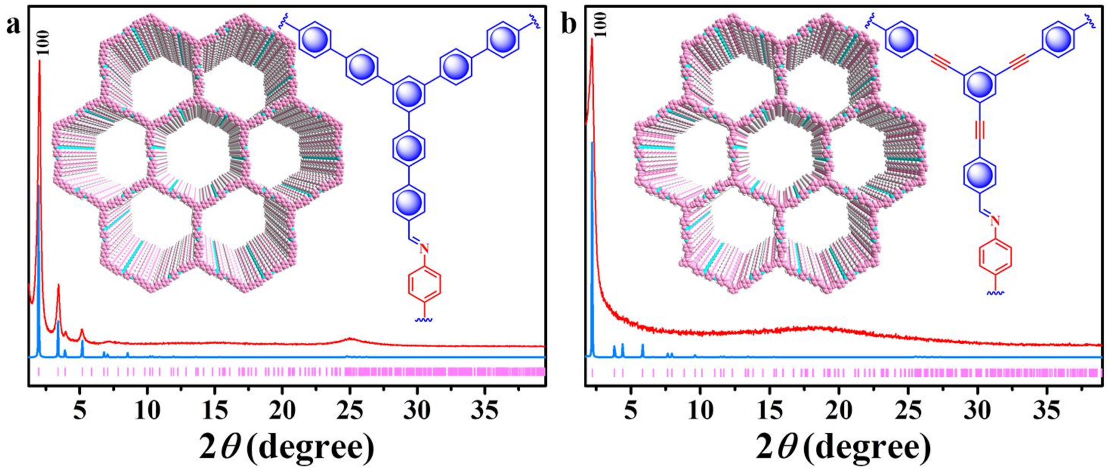

Supplementary Fig. 29 | PXRD patterns of DAFB-Pa (a) and BTTA-Pa (b). The experimental (red line) profiles, the eclipsed stacking mode (blue line), and the Bragg positions (pink bar). The PXRD patterns of DAFB-Pa and BTTA-Pa showed highly intense reflection peaks at about 2.0 and 2.2 (2*θ*), respectively, which could be ascribed to the (100) facet, indicating the highly crystalline skeleton of DAFB-Pa and BTTA-Pa. The experimental PXRD patterns agreed well with the simulated AA stacking models, suggesting the validity of the slipped eclipsed AA stacking.

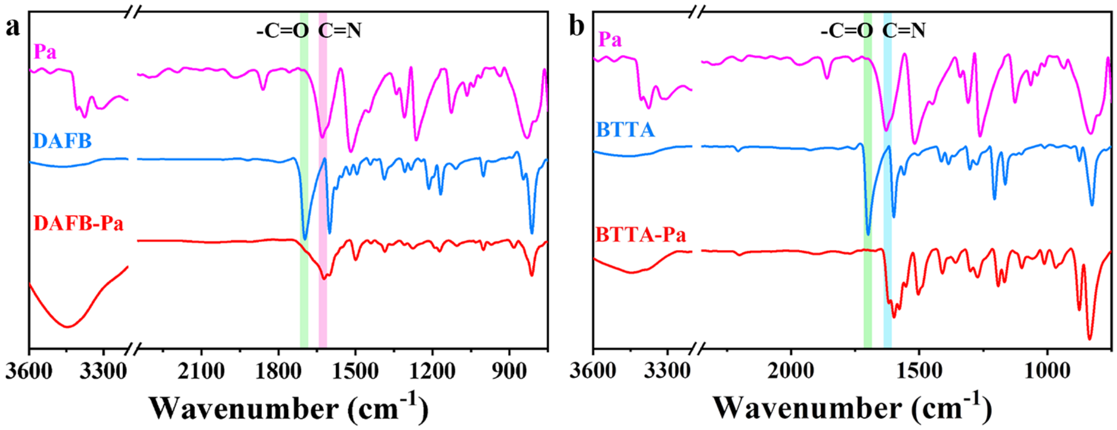

Supplementary Fig. 30 | FT-IR spectra of Pa, DAFB, and DAFB-Pa (a). FT-IR spectra of Pa, BTTA, and BTTA-Pa (b). In the FT-IR spectra of DAFB-Pa and BTTA-Pa, the stretching vibration peak of C=O (*ca.* 1696 cm^{-1}) completely disappeared and the two new peaks of C=N (*ca.* 1620 cm^{-1}) were found, confirming that these COFs were completely condensed and the imine linkages were successfully formed.

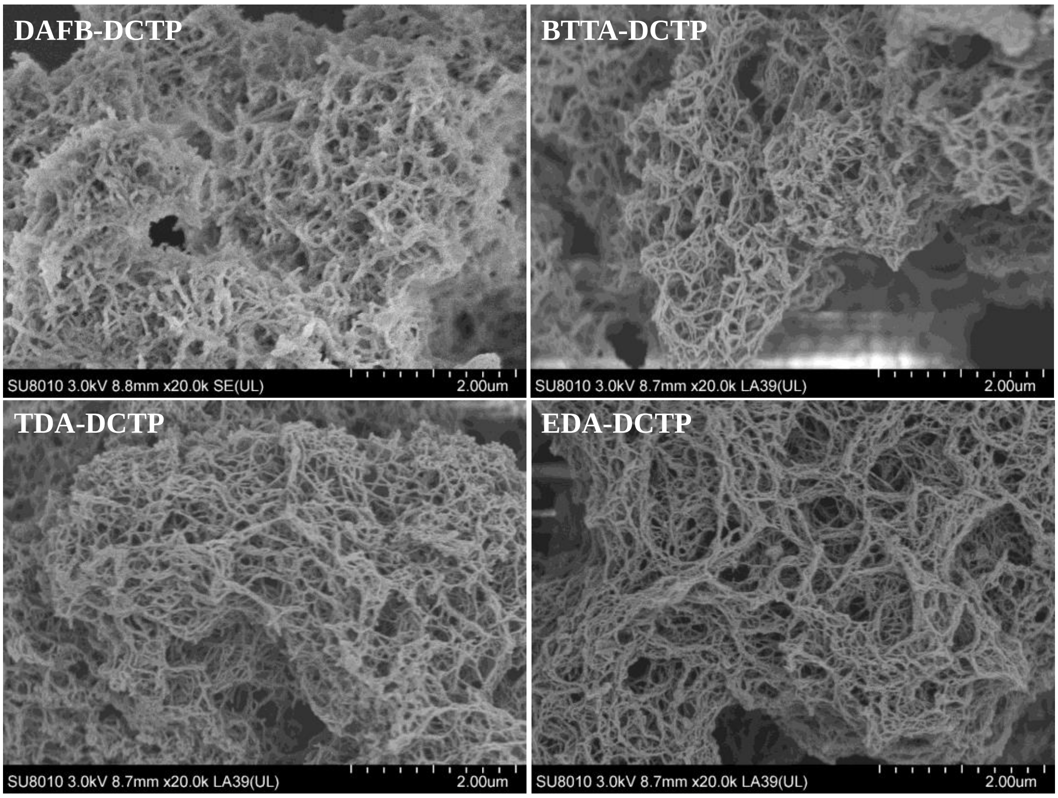

Supplementary Fig. 31 | SEM images of DAFB-DCTP, BTTA-DCTP, TDA-DCTP and EDA-DCTP.

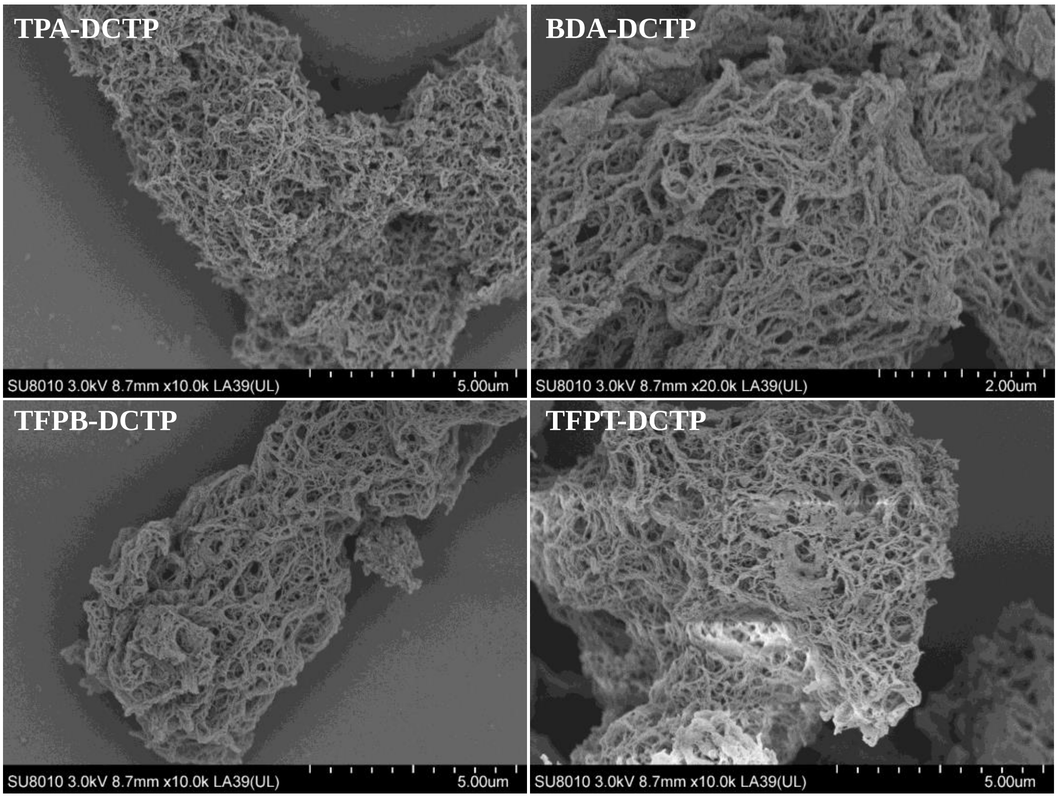

Supplementary Fig. 32 | SEM images of TPA-DCTP, BDA-DCTP, TFPB-DCTP and TFPT-DCTP.

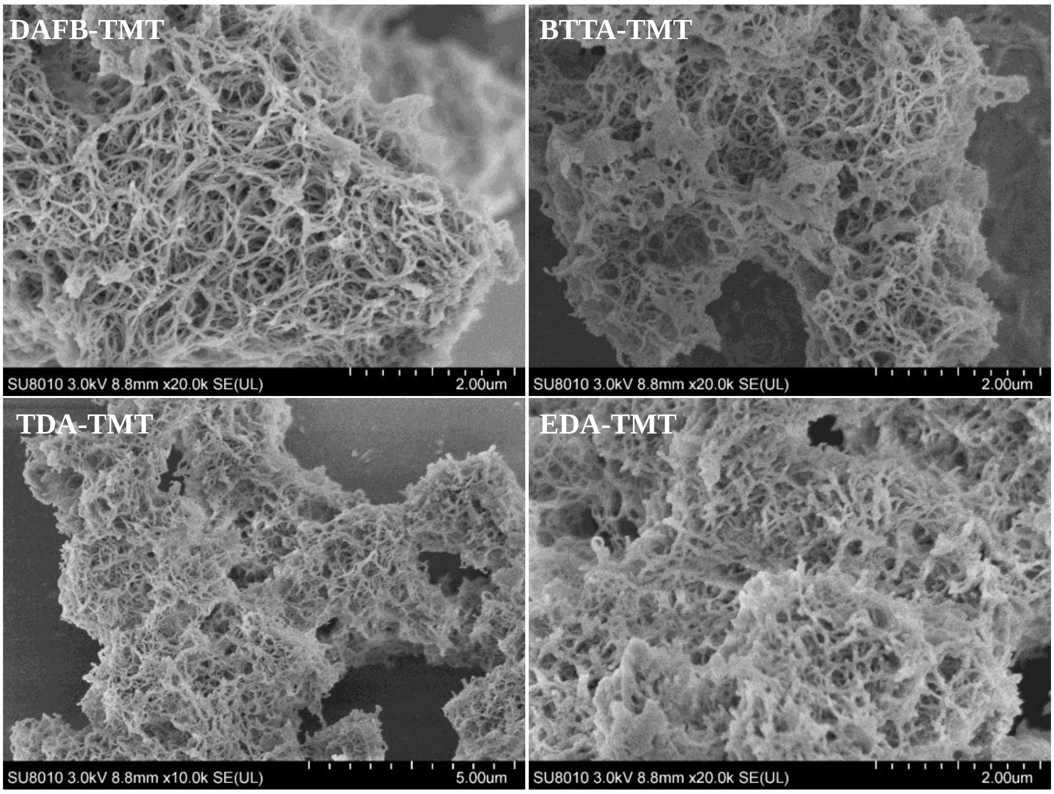

Supplementary Fig. 33 | SEM images of DAFB-TMT, BTTA-TMT, TDA-TMT and EDA-TMT.

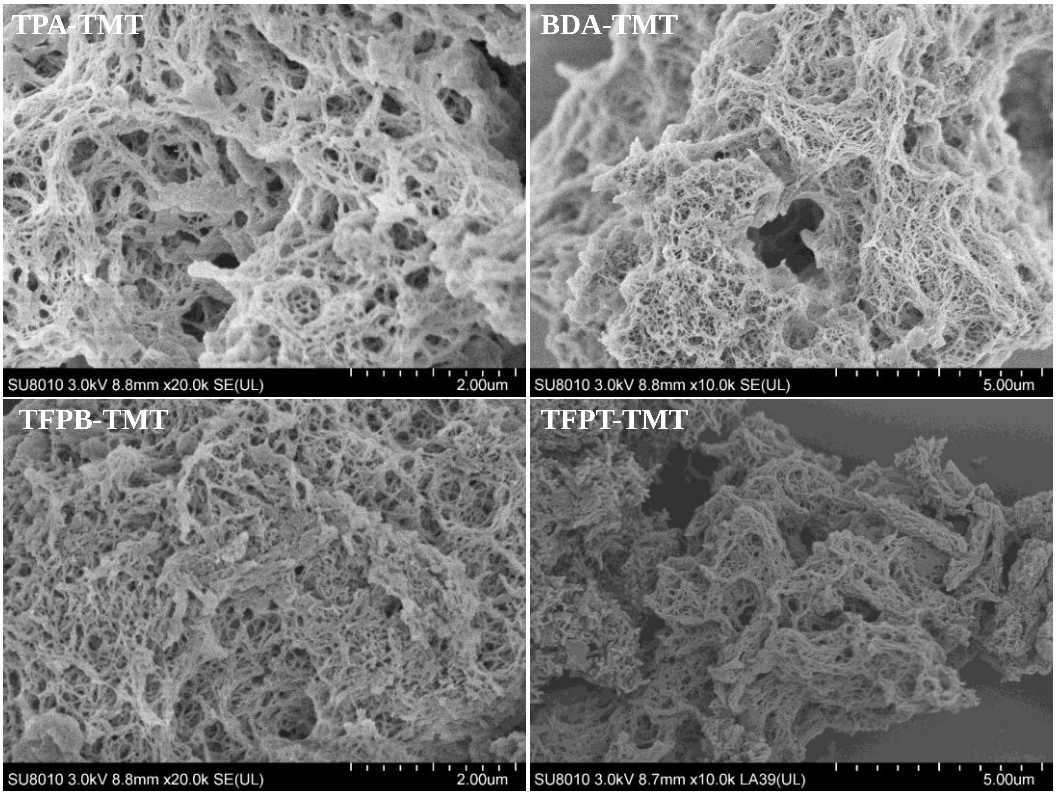

Supplementary Fig. 34 | SEM images of TPA-TMT, BDA-TMT, TFPB-TMT and TFPT-TMT.

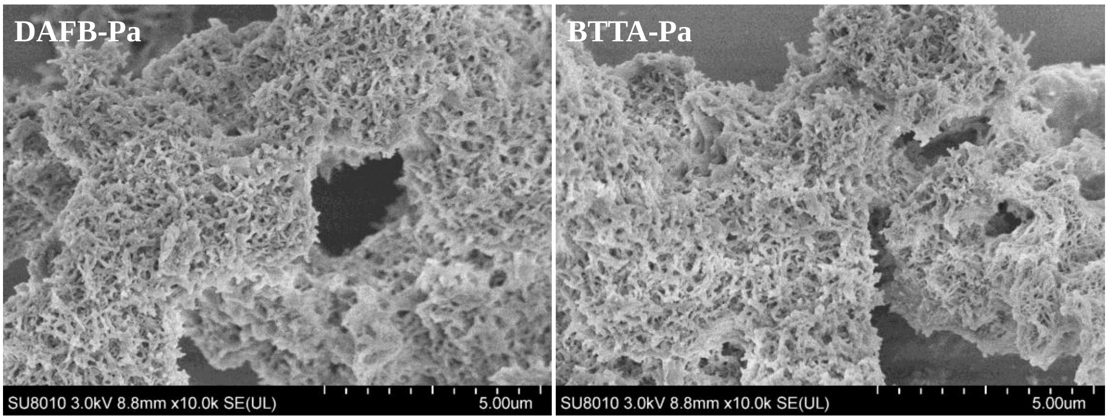

Supplementary Fig. 35 | SEM images of DAFB-Pa and BTTA-Pa.

Supplementary Table 17 | Fractional atomic coordinates for the eclipsed AA-stacking unit cell of DAFB-Pa.

Space group: P6/M ***a = b = 52.0567 Å, c = 3.5960 Å*** ***α = β = 90°, γ = 120°***							
**Atom**	***x* (Å)**	***y* (Å)**	***z* (Å)**	**Atom**	***x* (Å)**	***y* (Å)**	***z* (Å)**
C1	0.30264	-0.34637	0.5	C17	0.48507	-0.03127	0.5
C2	0.31596	-0.36351	0.5	C18	0.46907	-0.01628	0.5
C3	0.36023	-0.36974	0.5	C19	0.48374	0.01474	0.5
C4	0.3894	-0.35843	0.5	H20	0.30265	-0.38664	0.5
C5	0.40248	-0.37604	0.5	H21	0.40194	-0.3362	0.5
C6	0.38665	-0.40552	0.5	H22	0.42474	-0.36686	0.5
C7	0.35747	-0.41691	0.5	H23	0.34494	-0.43931	0.5
C8	0.34448	-0.39921	0.5	H24	0.32236	-0.40847	0.5
C9	0.40052	-0.42434	0.5	H25	0.36106	-0.46392	0.5
C10	0.38399	-0.45431	0.5	H26	0.38402	-0.49494	0.5
C11	0.39713	-0.472	0.5	H27	0.46655	-0.42071	0.5
C12	0.427	-0.46013	0.5	H28	0.44357	-0.38968	0.5
C13	0.44354	-0.43024	0.5	H29	0.50173	-0.07118	0.5
C14	0.43044	-0.41251	0.5	H30	0.44509	-0.02886	0.5
C15	0.47918	-0.08032	0.5	H31	0.47062	0.02551	0.5
N16	0.46881	-0.06311	0.5				

Supplementary Table 18 | Fractional atomic coordinates for the eclipsed AA-stacking unit cell of BTTA-Pa.

Space group: P6/M ***a = b = 46.2549 Å, c = 3.4936 Å*** ***α = β = 90°, γ = 120°***							
**Atom**	***x* (Å)**	***y* (Å)**	***z* (Å)**	**Atom**	***x* (Å)**	***y* (Å)**	***z* (Å)**
C1	-0.62593	-0.39293	0.5	C13	-0.63808	-0.37517	0.5
C2	-0.61143	-0.41411	0.5	C14	-0.65256	-0.35398	0.5
C3	-0.57672	-0.40004	0.5	C15	-0.63193	-0.31923	0.5
C4	-0.5626	-0.42064	0.5	H16	-0.56064	-0.37318	0.5
C5	-0.58312	-0.45556	0.5	H17	-0.5357	-0.40941	0.5
C6	-0.61794	-0.46948	0.5	H18	-0.63422	-0.4963	0.5
C7	-0.63201	-0.44884	0.5	H19	-0.6589	-0.45989	0.5
C8	-0.56873	-0.47775	0.5	H20	-0.5857	-0.50436	0.5
N9	-0.53655	-0.46542	0.5	H21	-0.47147	-0.43826	0.5
C10	-0.51884	-0.4836	0.5	H22	-0.43829	-0.466	0.5
C11	-0.48396	-0.46524	0.5	H23	-0.60504	-0.30832	0.5
C12	-0.46511	-0.48129	0.5				

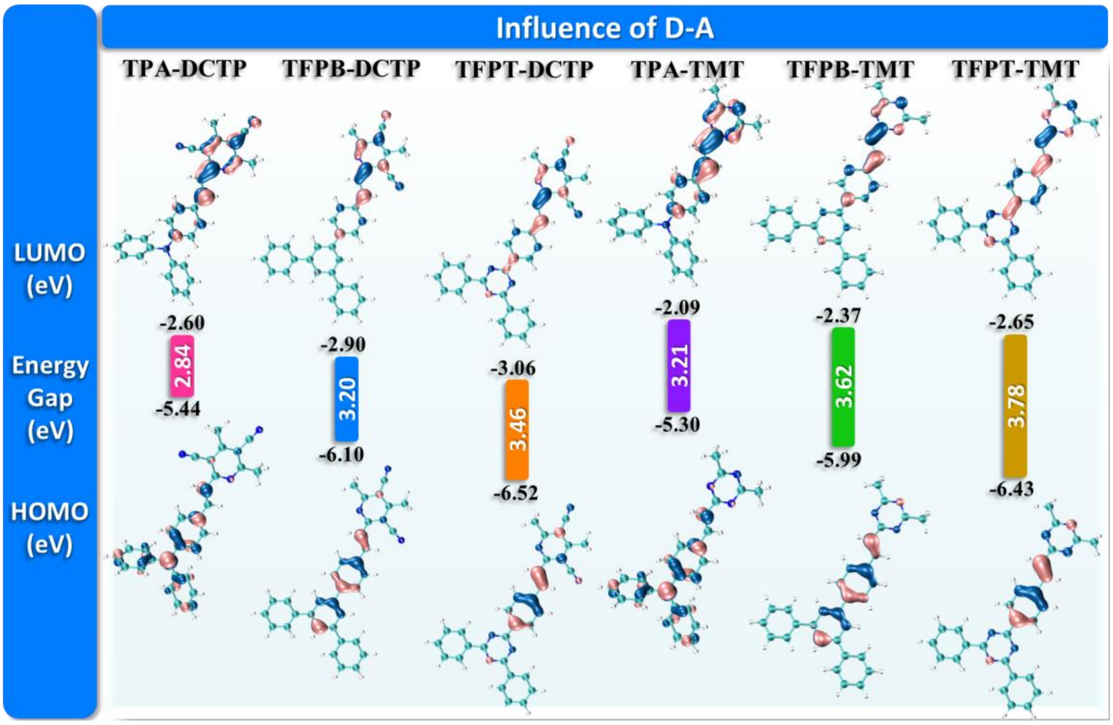

Supplementary Fig. 36 | DFT calculations to COFs' building blocks. The analysis of HOMOs, LUMOs and calculated energy gaps.

Supplementary Table 19 | Excited states analysis of COFs building blocks.

	TPA-DCTP	TFPB-DCTP	TFPT-DCTP	TPA-TMT	TFPB-TMT	TFPT-TMT
**t (Å)**	1.80	0.69	-2.83	1.48	-0.71	-3.03
**S_r (a.u.)**	0.55	0.65	0.78	0.58	0.73	0.76
**D (Å)**	5.34	4.71	1.05	4.71	3.17	0.63
**H (Å)**	3.91	4.30	4.11	3.62	4.12	3.88
**Δ_σ (Å)**	-0.20	-0.67	0.48	-0.14	-0.25	0.96
**E (eV)**	2.61	2.94	3.19	2.93	3.36	3.52
**Electron hole distribution**	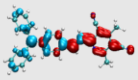	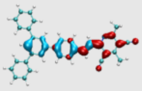	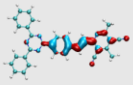	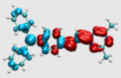	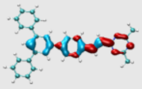	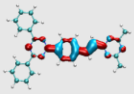

t index represents the separation degree between holes and electrons. The larger *t* index indicated more significant separation of holes and electrons, and a smaller *t* indicats a higher overlap of holes and electrons.

S_r index represents the overlap degree of holes and electrons.

D index represents the distance between the hole and the electronic centroid.

H index represents the overall average distribution breadths of electrons and holes.

Δ_σ index represents the difference in the overall spatial distributions of electrons and holes.

E represents the excitation energy of this state.

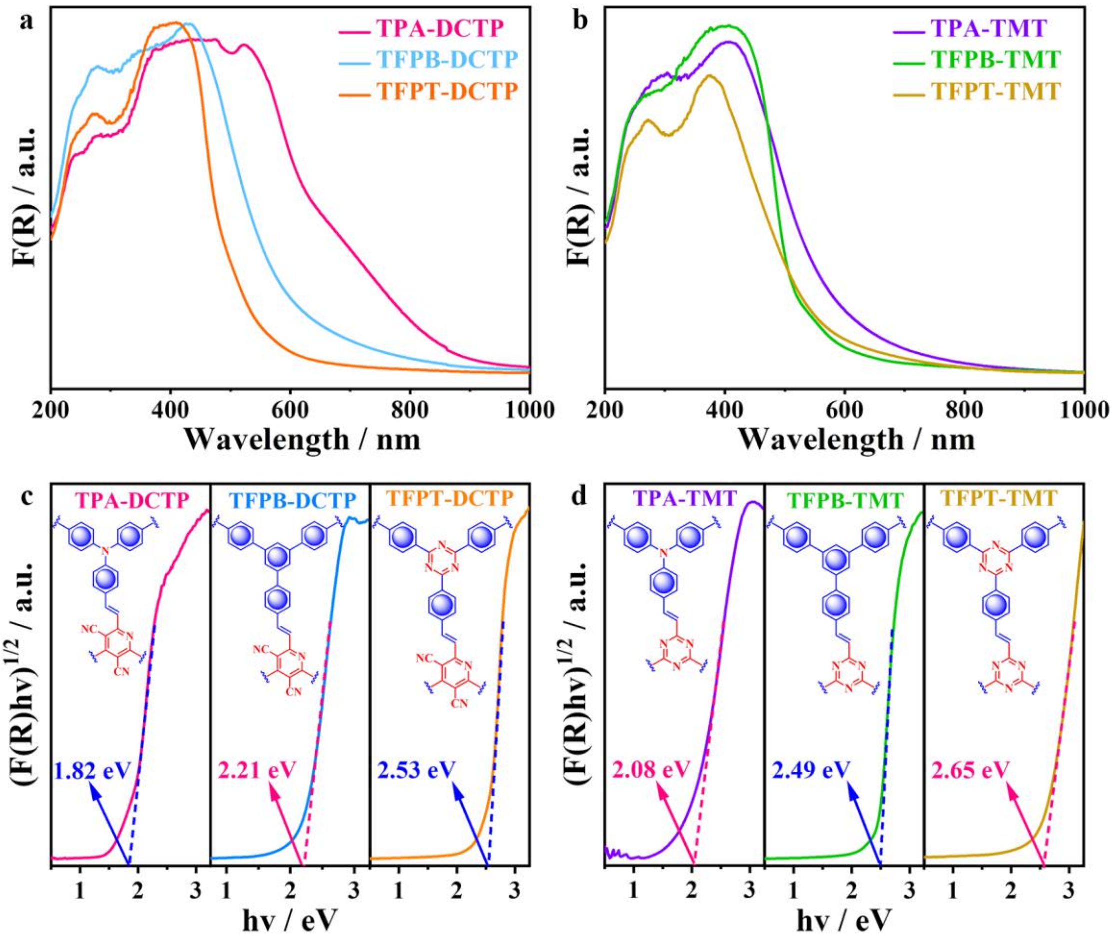

Supplementary Fig. 37 | UV/Vis DRS of TPA-DCTP, TFPB-DCTP and TFPT-DCTP (a). UV/Vis DRS of TPA-TMT, TFPB-TMT and TFPT-TMT (b). Band gaps of TPA-DCTP, TFPB-DCTP and TFPT-DCTP determined from the Kubelka-Munk function (c). Band gaps of TPA-TMT, TFPB-TMT and TFPT-TMT determined from the Kubelka-Munk function (d). Since the focus is on photoelectric activity research, we fully assessed the olefin-linked COFs optical properties. The UV/Vis DRS analysis of TPA-DCTP, TFPB-DCTP and TFPT-DCTP demonstrated that the absorption edge of *ca.* 890 nm for TPA-DCTP was significantly red-shifted by *ca.* 381 nm over that of *ca.* 509 nm for TFPT-DCTP, which might be due to the integration of the strong donor unit in the form of triphenylamine moiety significantly reducing the band gap and boosting charge transfer. Similarly, UV/Vis DRS analysis of TPA-TMT, TFPB-TMT and TFPT-TMT showed that the absorption edge of *ca.* 626 nm for TPA-TMT was significantly red-shifted by *ca.* 54 nm over that of *ca.* 572 nm for TFPT-TMT, further indicating that the introduction of triphenylamine moiety as a

strong donor unit significantly reducing the band gap and boosting charge transfer. Meanwhile, the band gaps of TPA-DCTP, TFPB-DCTP and TFPT-DCTP were calculated from the Kubelka-Munk function to be 1.82, 2.21 and 2.53 eV, respectively, which are much lower than the corresponding triazine-based COFs, indicating that the introduction of DCTP monomer as a strong acceptor unit in the framework significantly reducing the band gap and boosting charge transfer. These low band-gaps D-A COFs may therefore serve as new ECL emitters.

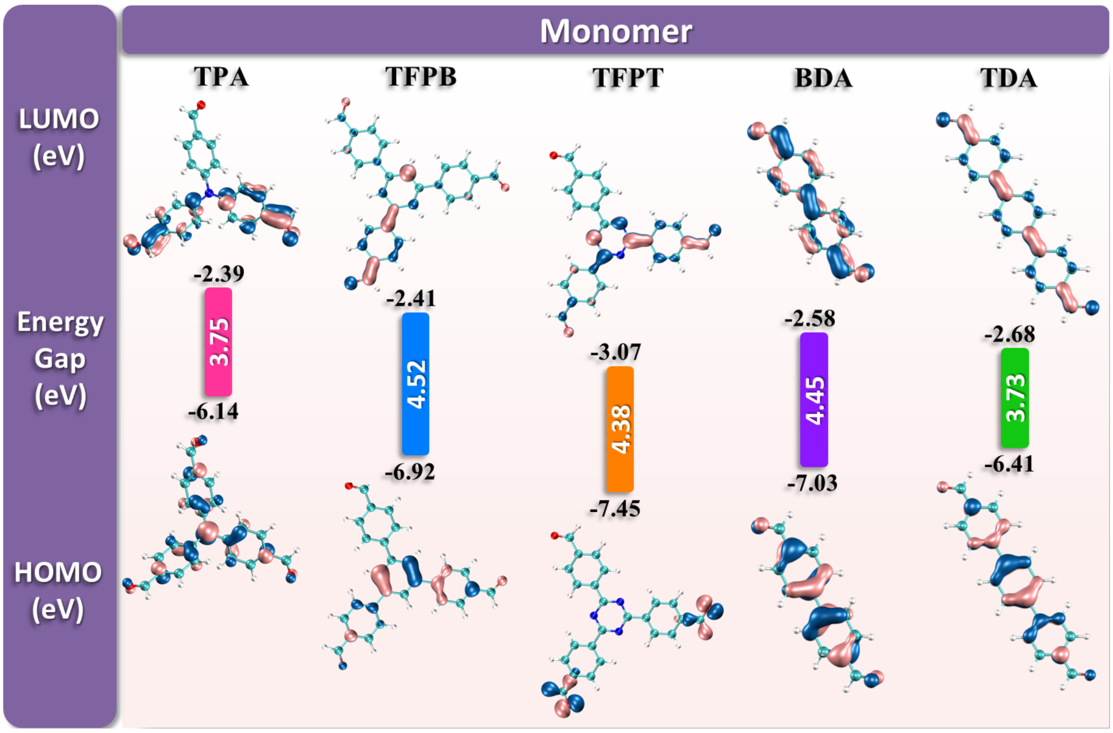

Supplementary Fig. 38 | DFT calculations to monomers. The analysis of HOMOs, LUMOs and calculated energy gaps.

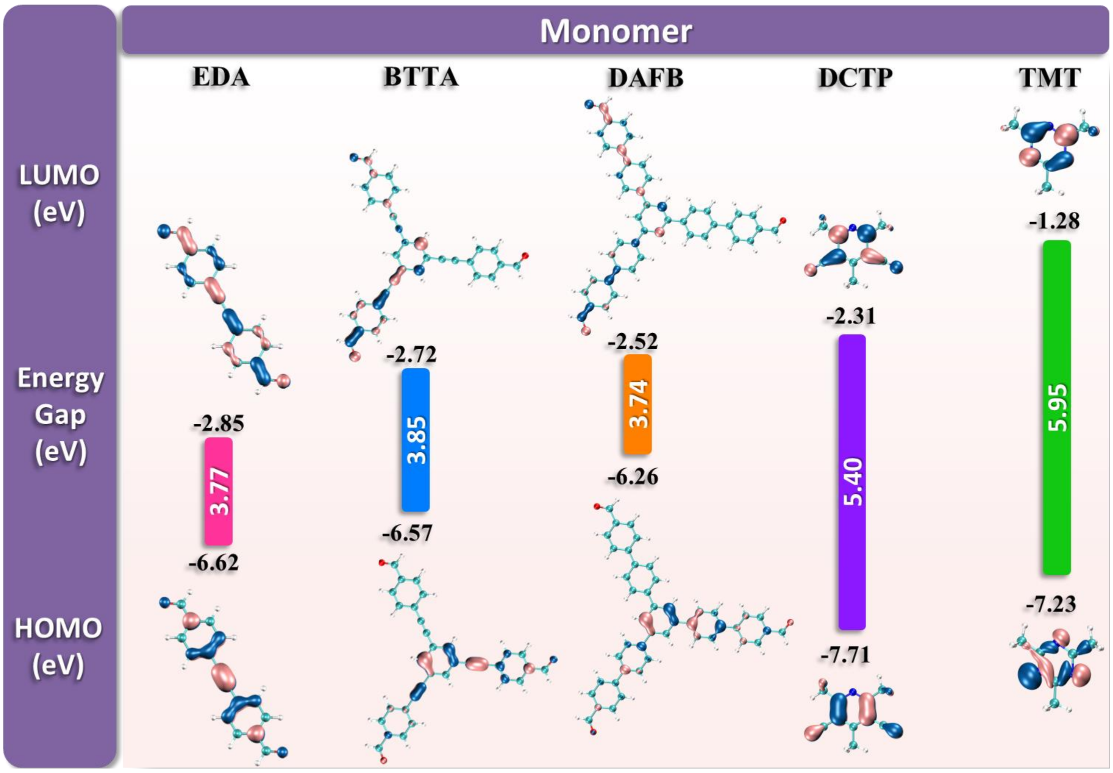

Supplementary Fig. 39 | DFT calculations to monomers. The analysis of HOMOs, LUMOs and calculated energy gaps.

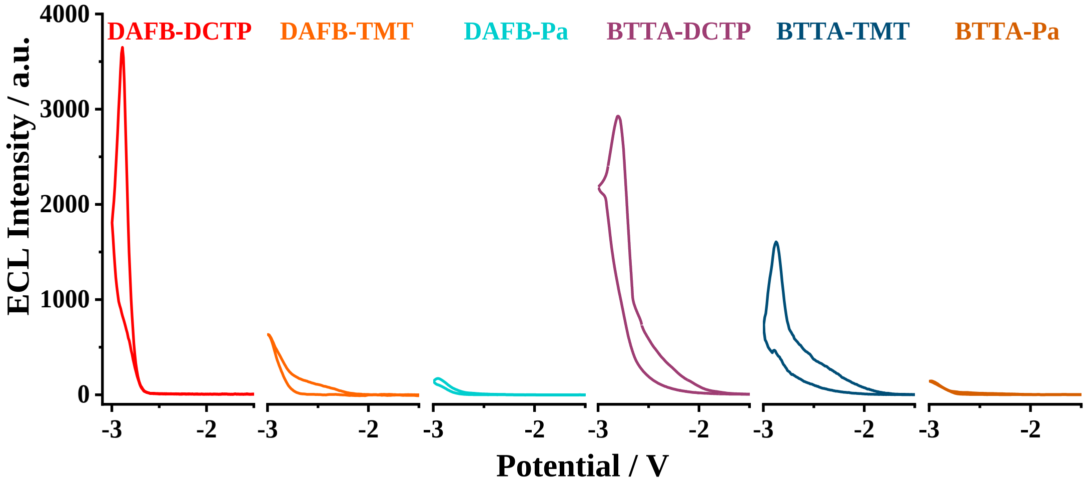

Supplementary Fig. 40 | Comparison of ECL intensities of COFs (DAFB-DCTP, DAFB-TMT, DAFB-Pa, BTTA-DCTP, BTTA-TMT, BTTA-Pa) in 0.1 M PBS with 0.1 M KCl between 0 V and -3.0 V, pH=7.5, PMT: 800 V, potential scan rate: 100 mV s^{-1}.

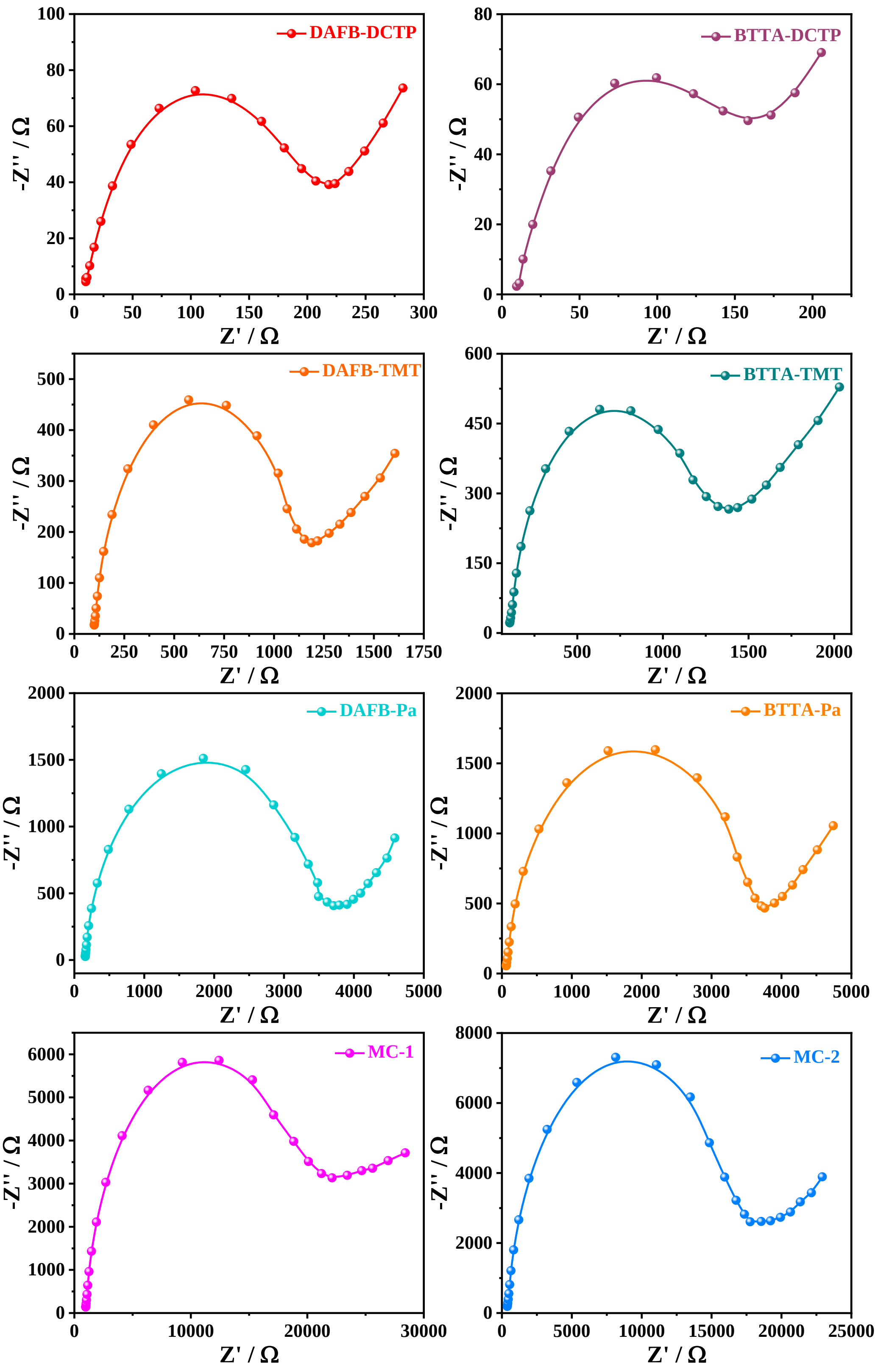

Supplementary Fig. 41 | EIS curves of DAFB-DCTP, BTTA-DCTP, DAFB-TMT, BTTA-TMT, DAFB-Pa, BTTA-Pa, MC-1, MC-2.

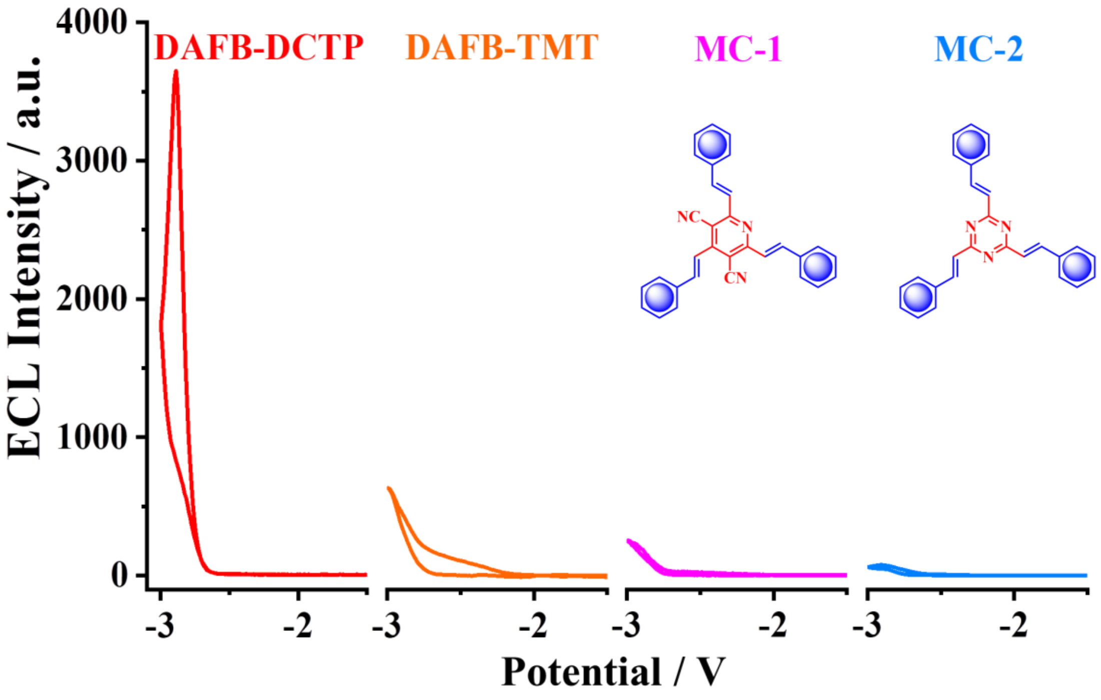

Supplementary Fig. 42 | Comparison of ECL intensities of COFs and model compounds (MC-1, MC-2) in 0.1 M PBS with 0.1 M KCl between 0 V and -3.0 V. pH 7.5, PMT: 800 V, potential scan rate: 100 mV s^{-1}.

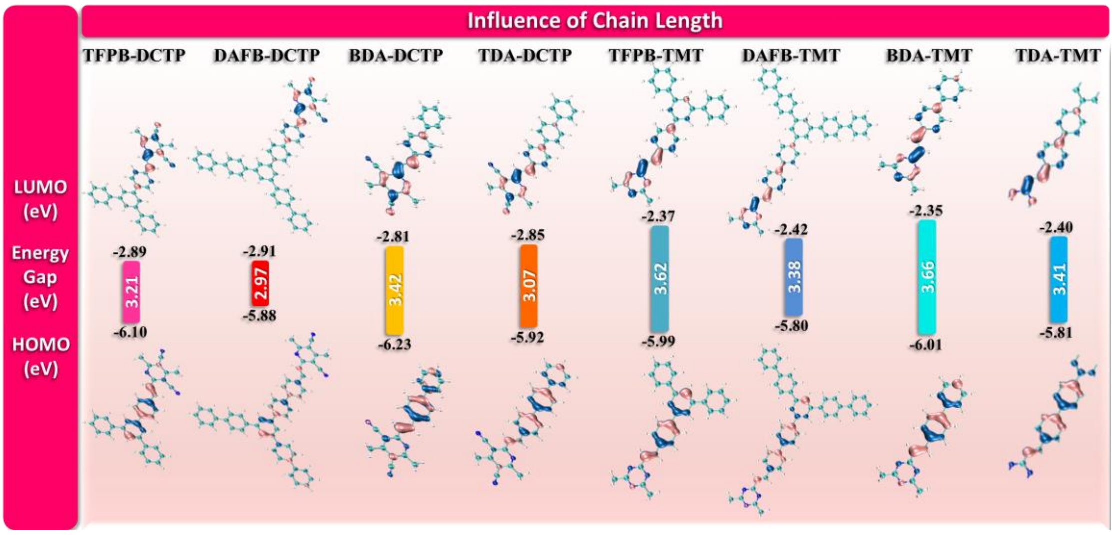

Supplementary Fig. 43 | DFT calculations to COFs' building blocks. The analysis of HOMOs, LUMOs and calculated energy gaps.

Supplementary Table 20 | Excited states analysis of COFs building blocks.

	TFPB-DCTP	DAFB-DCTP	BDA-DCTP	TDA-DCTP	TFPB-TMT	DAFB-TMT	BDA-TMT	TDA-TMT
t (Å)	0.69	3.95	-0.74	0.93	-0.71	0.52	-1.21	-0.52
S_r (a.u.)	0.65	0.51	0.72	0.64	0.73	0.69	0.76	0.72
D (Å)	4.71	8.83	3.03	5.50	3.17	5.63	2.44	3.96
H (Å)	4.30	5.33	3.99	4.75	4.12	5.48	3.84	4.64
Δ_σ (Å)	-0.67	-1.92	-0.43	-0.62	-0.25	-0.88	0.05	-0.14
E (eV)	2.94	2.72	3.18	2.83	3.36	3.09	3.44	3.15
Electron hole distribution	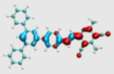	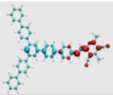	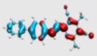	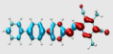	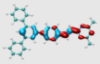	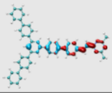	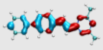	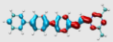

t index represents the separation degree between holes and electrons. The larger *t* index indicated more significant separation of holes and electrons, and a smaller *t* indicats a higher overlap of holes and electrons.

S_r index represents the overlap degree of holes and electrons.

D index represents the distance between the hole and the electronic centroid.

H index represents the overall average distribution breadths of electrons and holes.

Δ_σ index represents the difference in the overall spatial distributions of electrons and holes.

E represents the excitation energy of this state.

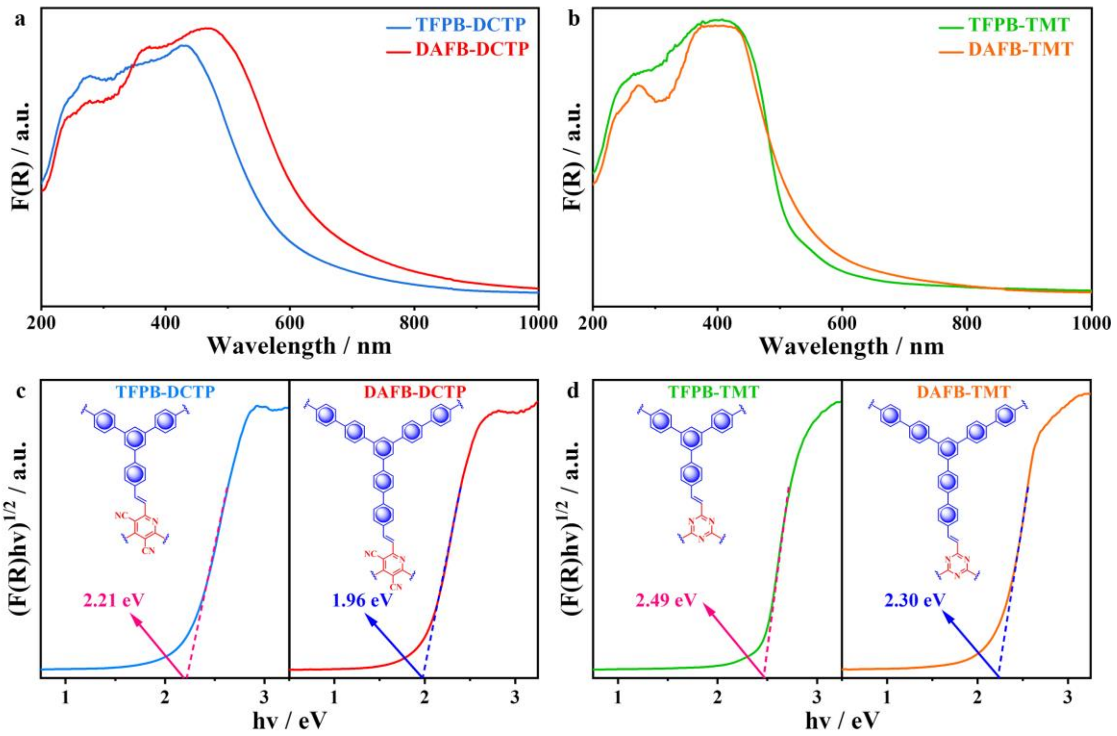

Supplementary Fig. 44 | UV/Vis DRS of TFPB-DCTP and DAFB-DCTP (a). UV/Vis DRS of TFPB-TMT and DAFB-TMT. (b). Band gaps of TFPB-DCTP and DAFB-DCTP determined from the Kubelka-Munk function (c). Band gaps of TFPB-TMT and DAFB-TMT determined from the Kubelka-Munk function (d). Since the focus is on photoelectric activity research, we fully assessed the olefin-linked COFs optical properties. The UV/Vis DRS analysis of TFPB-DCTP and DAFB-DCTP demonstrated that the absorption edge of *ca.* 679 nm for DAFB-DCTP was significantly red-shifted by *ca.* 88 nm over that of *ca.* 491 nm for TFPB-DCTP, which is likely due to the larger extended π-conjugation in DAFB-DCTP greatly enhancing π - electron delocalization within the 2D olefin-linked skeleton. Similarly, UV/Vis DRS analysis of TFPB-TMT and DAFB-TMT showed that the absorption edge of *ca.* 565 nm for DAFB-TMT was significantly red-shifted by *ca.* 39 nm over that of *ca.* 526 nm for TFPB-TMT, further indicating that the larger extended π-conjugation in DAFB-TMT greatly enhancing π - electron delocalization within the 2D olefin-linked skeleton. Meanwhile, the band gaps of TFPB-DCTP and DAFB-DCTP were calculated from the Kubelka-Munk function to be 2.21 and 1.96 eV, respectively, which are much lower than the corresponding triazine-based COFs, indicating that the introduction of DCTP monomer as a strong acceptor unit in the framework

significantly reducing the band gap and boosting charge transfer. These highly extended π-conjugation D-A COFs may therefore serve as new ECL emitters.

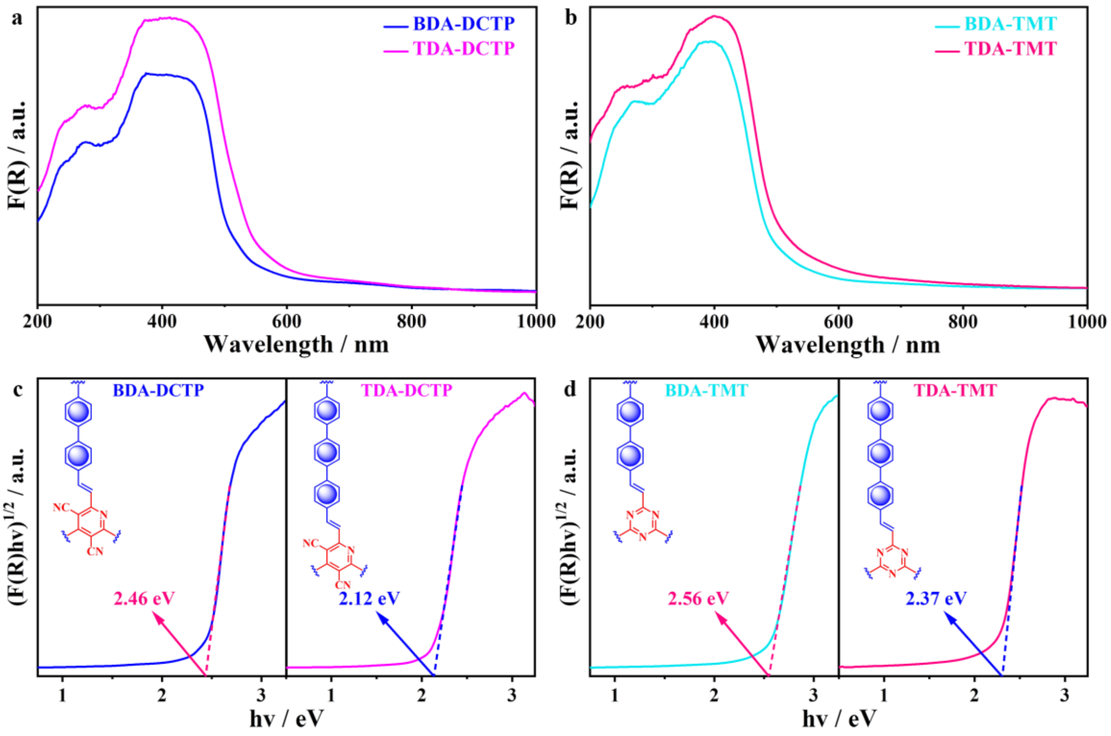

Supplementary Fig. 45 | UV/Vis DRS of BDA-DCTP and TDA-DCTP (a). UV/Vis DRS of BDA-TMT and TDA-TMT (b). Band gaps of BDA-DCTP and TDA-DCTP determined from the Kubelka-Munk function (c). Band gaps of BDA-TMT and TDA-TMT determined from the Kubelka-Munk function (d). Since the focus is on photoelectric activity research, we fully assessed the olefin-linked COFs optical properties. The UV/Vis DRS analysis of BDA-DCTP and TDA-DCTP demonstrated that the absorption edge of *ca.* 552 nm for TDA-DCTP was significantly red-shifted by *ca.* 40 nm over that of *ca.* 512 nm for BDA-DCTP, which is likely due to the larger extended π-conjugation in TDA-DCTP greatly enhancing π - electron delocalization within the 2D olefin-linked skeleton. Similarly, UV/Vis DRS analysis of BDA-TMT and TDA-TMT showed that the absorption edge of *ca.* 514 nm for TDA-TMT was significantly red-shifted by *ca.* 18 nm over that of *ca.* 496 nm for BDA-TMT, further indicating that the larger extended π-conjugation in TDA-TMT greatly enhancing π - electron delocalization within the 2D olefin-linked skeleton. Meanwhile, the band gaps of BDA-TMT and TDA-TMT were calculated from the Kubelka-Munk function

to be 2.56 and 2.37 eV, respectively, which are much lower than the corresponding triazine-based COFs, indicating that the introduction of DCTP monomer as a strong acceptor unit in the framework significantly reducing the band gap and boosting charge transfer. These highly extended π-conjugation D-A COFs may therefore serve as new ECL emitters.

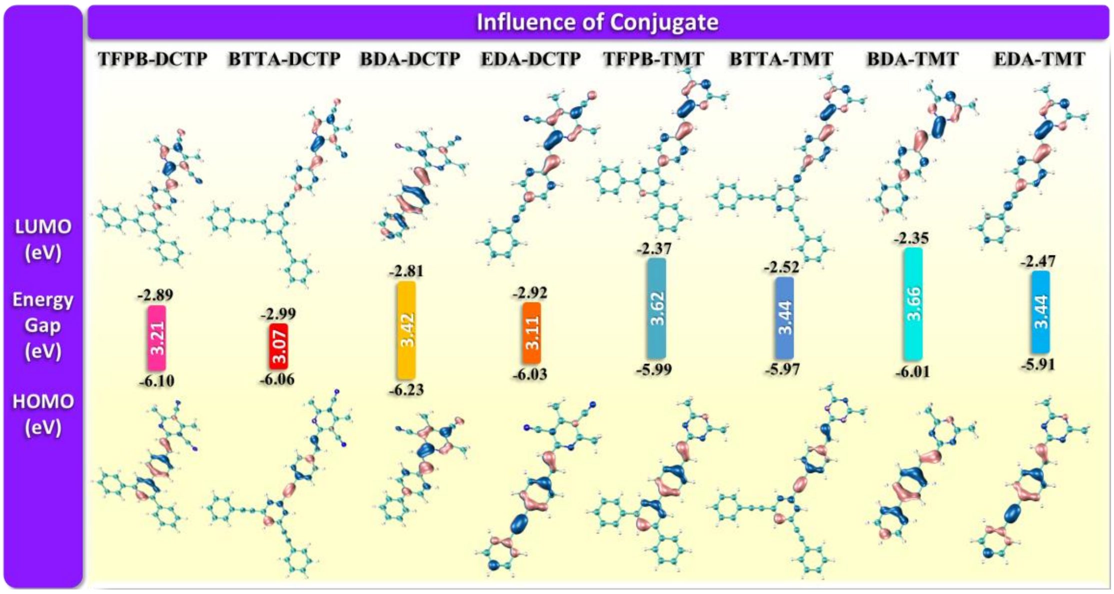

Supplementary Fig. 46 | DFT calculations to COFs' building blocks. The analysis of HOMOs, LUMOs and calculated energy gaps.

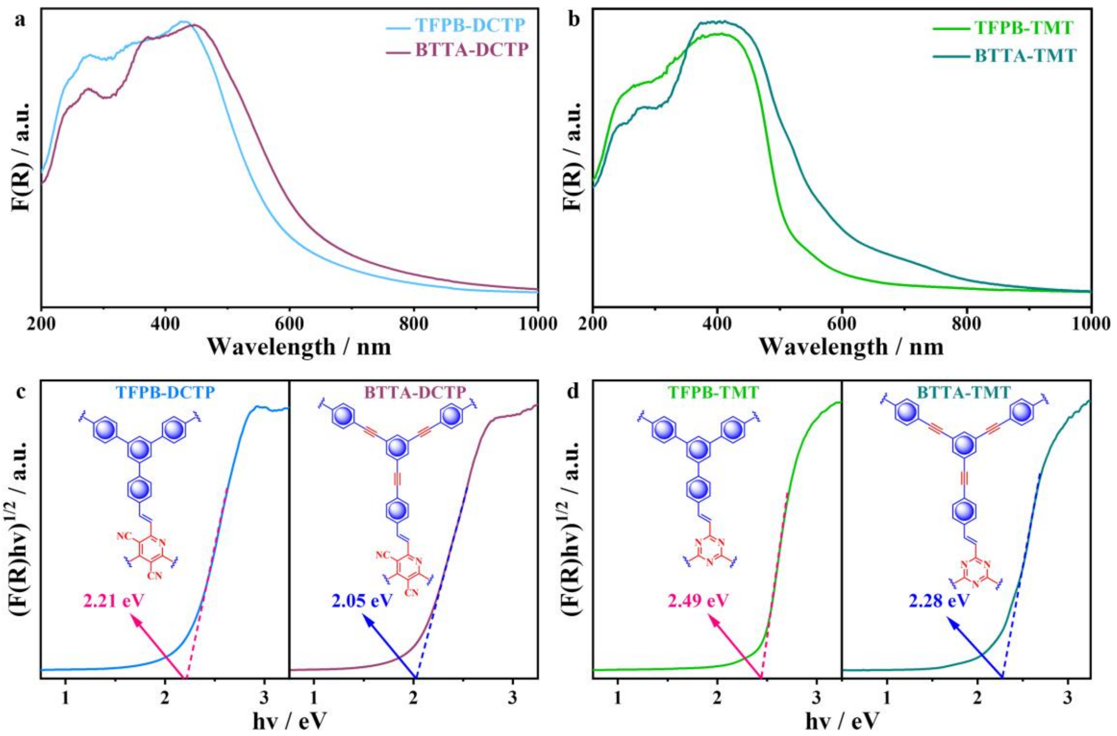

Supplementary Fig. 47 | UV/Vis DRS of TFPB-DCTP and BTTA-DCTP (a). UV/Vis DRS of TFPB-TMT and BTTA-TMT (b). Band gaps of TFPB-DCTP and BTTA-DCTP determined from the Kubelka-Munk function (c). Band gaps of TFPB-TMT and BTTA-TMT determined from the Kubelka-Munk function (d). Since the focus is on photoelectric activity research, we fully assessed the olefin-linked COFs optical properties. The UV/Vis DRS analysis of TFPB-DCTP and BTTA-DCTP demonstrated that the absorption edge of *ca.* 678 nm for BTTA-DCTP was significantly red-shifted by *ca.* 58 nm over that of *ca.* 620 nm for TFPB-DCTP, which is likely due to the highly planar π-conjugated acetylene (−C≡C−) moiety in BTTA-DCTP greatly enhancing π-electron delocalization within the 2D olefin-linked skeleton. Similarly, UV/Vis DRS analysis of TFPB-TMT and BTTA-TMT showed that the absorption edge of *ca.* 618 nm for BTTA-TMT was significantly red-shifted by *ca.* 93 nm over that of *ca.* 525 nm for TFPB-TMT, further indicating that the highly planar π-conjugated acetylene (−C≡C−) moiety in BTTA-DCTP greatly enhancing π-electron delocalization within the 2D olefin-linked skeleton. Meanwhile, the band gaps of TFPB-DCTP and BTTA-DCTP were calculated from the Kubelka-Munk function to be 2.21 and 2.05 eV, respectively, which are much lower than the corresponding triazine-based COFs, indicating that the introduction of DCTP

monomer as a strong acceptor unit in the framework significantly reducing the band gap and boosting charge transfer. These highly planar π - conjugated D-A COFs may therefore serve as new ECL emitters.

Supplementary Table 21 | Excited states analysis of COFs building blocks.

	TFPB-DCTP	BTTA-DCTP	BDA-DCTP	EDA-DCTP	TFPB-TMT	BTTA-TMT	BDA-TMT	EDA-TMT
t (Å)	0.69	1.75	-0.74	0.08	-0.71	-0.69	-1.21	-0.85
S_r (a.u.)	0.65	0.60	0.72	0.68	0.73	0.73	0.76	0.73
D (Å)	4.71	6.36	3.03	4.44	3.17	3.99	2.44	3.31
H (Å)	4.30	4.93	3.99	4.54	4.12	4.98	3.84	4.32
Δ_σ (Å)	-0.67	-1.16	-0.43	-0.34	-0.25	-0.36	0.05	0.03
E (eV)	2.94	2.81	3.18	2.89	3.36	3.18	3.44	3.22
Electron hole distribution	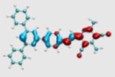	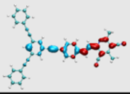	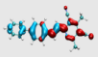	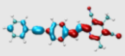	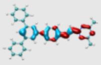	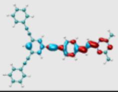	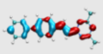	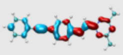

t index represents the separation degree between holes and electrons. The larger *t* index indicated more significant separation of holes and electrons, and a smaller *t* indicats a higher overlap of holes and electrons.

$\mathbf{S_r}$ index represents the overlap degree of holes and electrons.

D index represents the distance between the hole and the electronic centroid.

H index represents the overall average distribution breadths of electrons and holes.

$\boldsymbol{\Delta_\sigma}$ index represents the difference in the overall spatial distributions of electrons and holes.

E represents the excitation energy of this state.

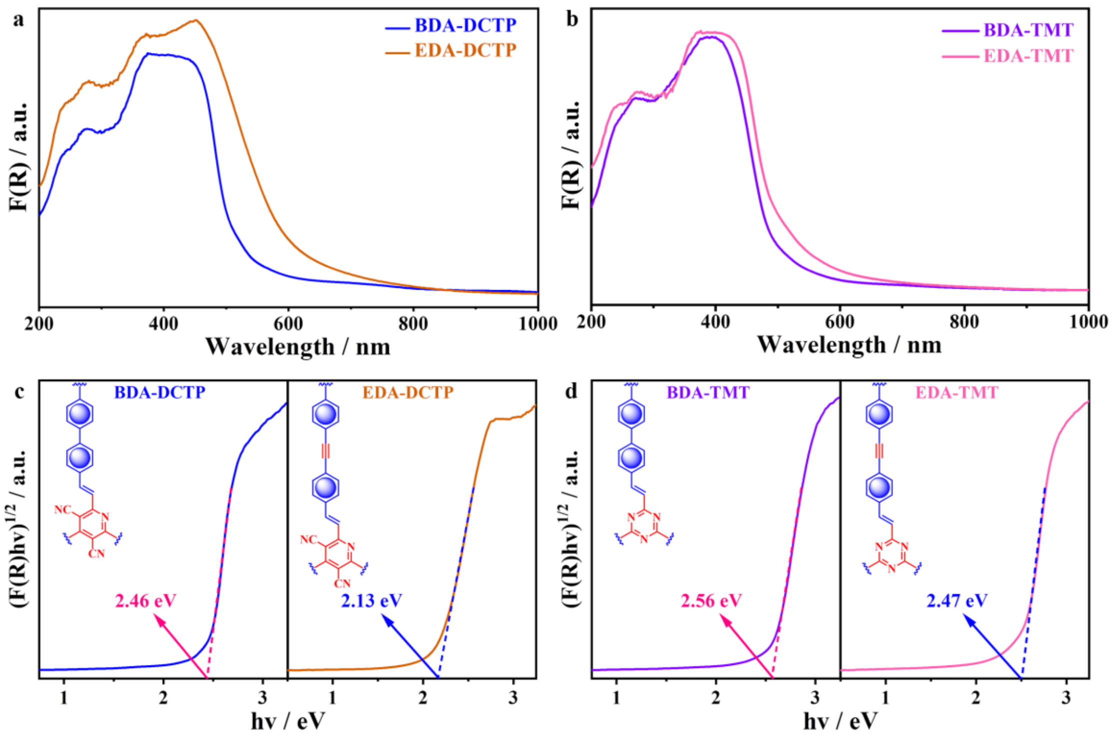

Supplementary Fig. 48 | UV/Vis DRS of BDA-DCTP and EDA-DCTP (a). UV/Vis DRS of BDA-TMT and EDA-TMT (b). Band gaps of BDA-DCTP and EDA-DCTP determined from the Kubelka-Munk function (c). Band gaps of BDA-TMT and EDA-TMT determined from the Kubelka-Munk function (d). Since the focus is on photoelectric activity research, we fully assessed the olefin-linked COFs optical properties. The UV/Vis DRS analysis of BDA-DCTP and EDA-DCTP demonstrated that the absorption edge of *ca.* 616 nm for EDA-DCTP was significantly red-shifted by *ca.* 101 nm over that of *ca.* 515 nm for BDA-DCTP, which is likely due to the highly planar π - conjugated acetylene (−C≡C−) moiety in EDA-DCTP greatly enhancing π - electron delocalization within the 2D olefin-linked skeleton. Similarly, UV/Vis DRS analysis of BDA-TMT and EDA-TMT showed that the absorption edge of *ca.* 579 nm for EDA-TMT was significantly red-shifted by *ca.* 70 nm over that of *ca.* 509 nm for BDA-TMT, further indicating that the highly planar π - conjugated acetylene (−C≡C−) moiety in EDA-TMT greatly enhancing π - electron delocalization within the 2D olefin-linked skeleton. Meanwhile, the band gaps of BDA-DCTP and EDA-DCTP were calculated from the Kubelka-Munk function to be 2.46 and 2.13 eV, respectively, which are much lower than the corresponding triazine-based COFs, indicating that the introduction of DCTP monomer as a strong

acceptor unit in the framework significantly reducing the band gap and boosting charge transfer. These highly planar π - conjugated D-A COFs may therefore serve as new ECL emitters.

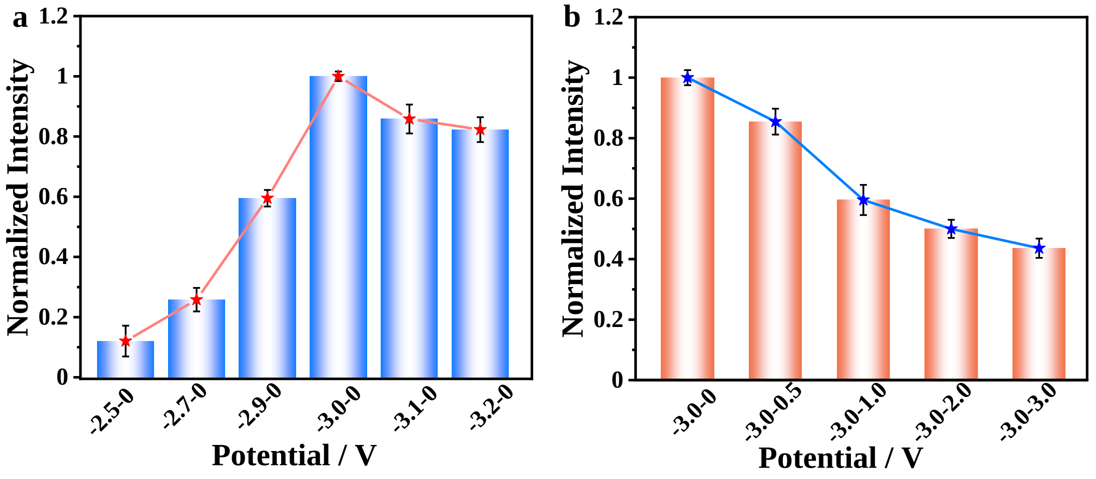

Supplementary Fig. 49 | Normalized ECL intensity of DAFB-DCTP under different cathode (a) and anode (b) scanning potentials in 0.1 M PBS with 0.1 M KCl; pH 7.5; scan rate, 100 mV s^{-1}, PMT=800 V. Error bars represent S.D. n=3 independent experiments.

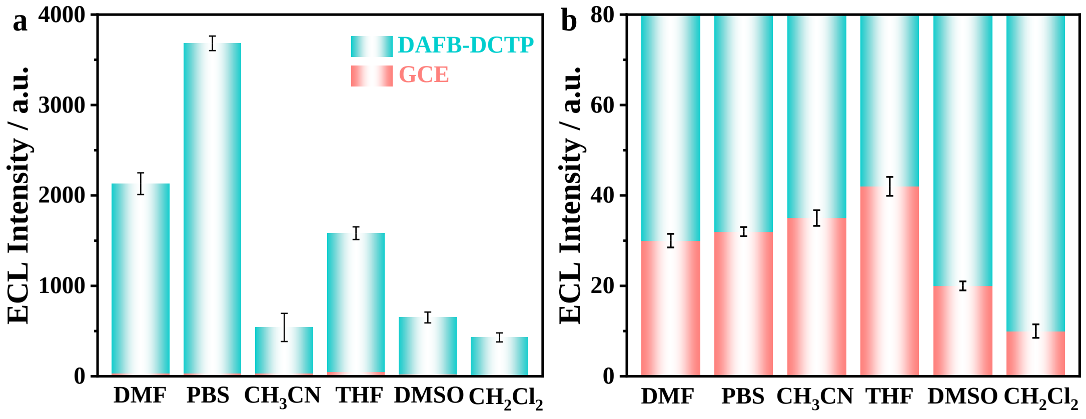

Supplementary Fig. 50 | (a) ECL intensity of DAFB-DCTP/GCE (green) and bare GCE (pink) in different solvents, PMT=800 V, scan rate, 100 mV s^{-1}. (b) is an enlarged view of the (a) from 0 to 80 a.u. Error bars represent S.D. n=3 independent experiments.

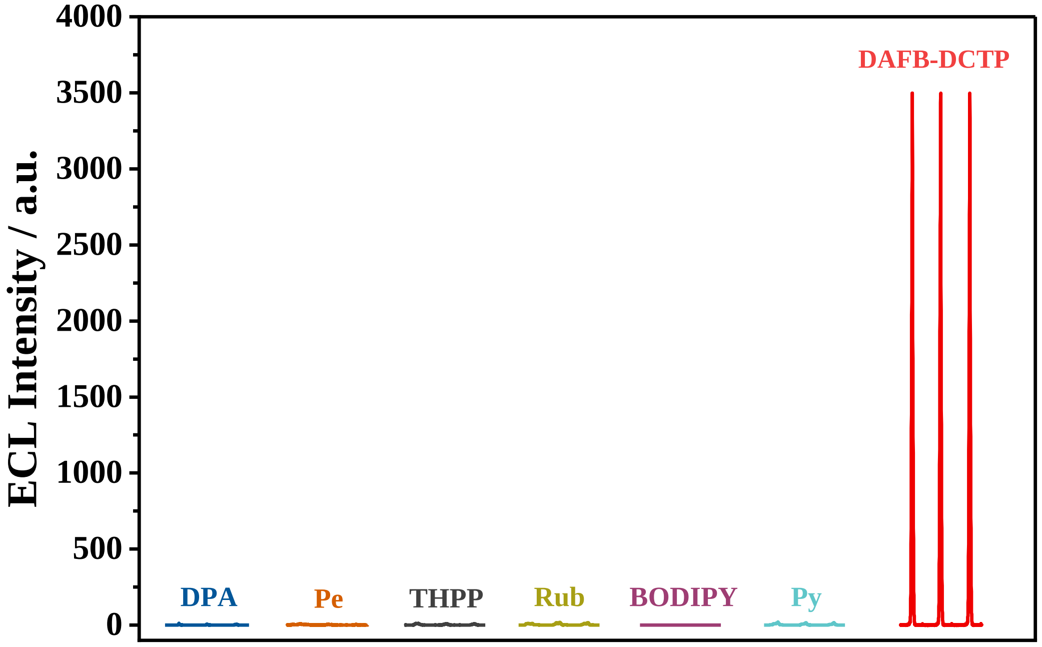

Supplementary Fig. 51 | The ECL−time curves of 1 mM 10 μL DPA, Pe, THPP, Rub, BODIPY, Py, and DAFB-DCTP modified GCE. All the buffer solutions throughout the ECL experiments were 0.1 M PBS with 0.1 M KCl between 0 V and -3.0 V. PMT= 800 V, pH 7.5, potential scan rate: 100 mV s^{-1}.

Supplementary Table 22 | Comparison of the Φ_{ECL} of different ECL emitters.

Type	ECL luminophores	Coreactant	Solvent	ECL efficiency (%)	Refs
Organic phosphor	pyrene-18C6	BPO	CH_3CN with 0.1 M $TBAPF_6$	24	5
	tetraphenylethylene	TEA	acetonitrile	1.35	6
	2,1,3-benzothiadiazole derivatives	BPO	MeCN	0.05-7	7
	TDAF-1/TDAF-2	BPO	MeCN:Bz (1:1,V:V)/ 0.1 M TBAP	3/8	8
	triple-component Polymer dots	TPrA	PBS	23.1	9
	polydopamine nanospheres	$K_2S_2O_8$	PBS	2.44	10
	TCPP	$K_2S_2O_8$	PBS	0.22	11
	spirobifluorene-bridged bipolar	Electrochemical annihilation	PhH:MeCN (4:1)	0.01-0.26	12
	phenanthrene derivatives	Electrochemical annihilation	MeCN:Bz (1:1) with 0.1 M $TBAPF_6$	0.4-25	13
Inorganic phosphor	$[Ir(df\text{-}ppy)_2(ptb)]^+$	TPrA	Dichloromethane:methyl alcohol (3:1)	6.2	14
	$[Ru(bpy)_2(PVP)_{10}]^{2+}$	$Na_2C_2O_4$	PBS	0.15	15
	[Ru-PANI]	TPA	PBS	1.00	16
	Cd-In-S NCs	TPA	PBS	2.10	17
	BN QDs	$K_2S_2O_8$	PBS	1.04	18
This work	DAFB-DCTP	dissolved oxygen	PBS	32.5	

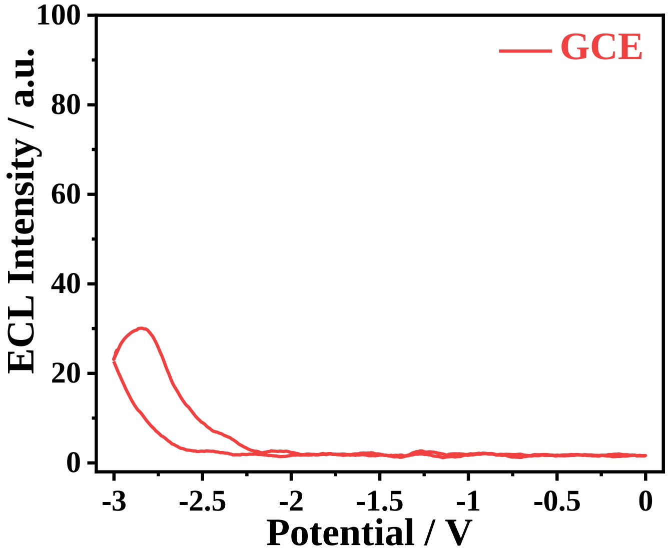

Supplementary Fig. 52 | The ECL− potential curves of GCE in 0.1 M PBS with 0.1 M KCl, pH 7.5, PMT=800 V, potential scan rate: 100 mV s^{-1}.

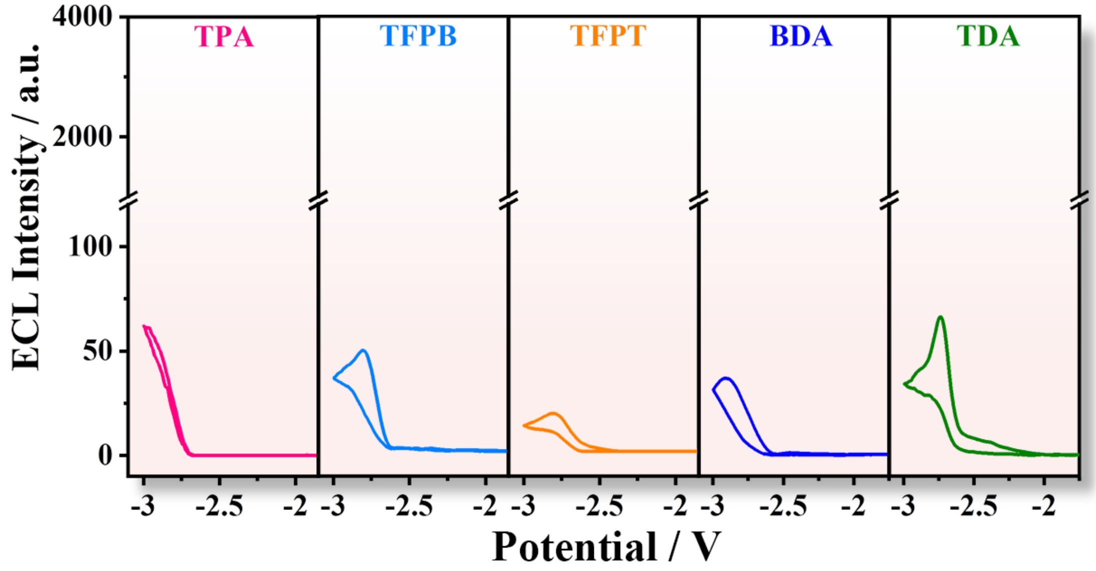

Supplementary Fig. 53 | The ECL− potential curves of 1 mM 10 μL TPA, TFPB, TFPT, BDA, and TDA modified GCE. All the buffer solutions throughout the ECL experiments were 0.1 M PBS with 0.1 M KCl between 0 V and -3.0 V. PMT: 800 V, pH 7.5, potential scan rate: 100 mV s^{-1}.

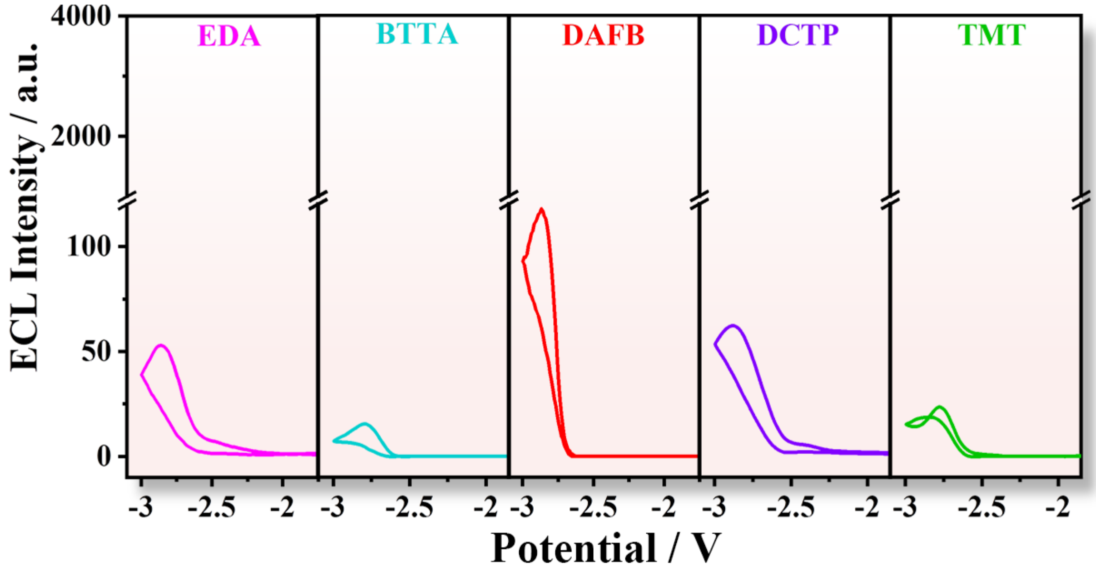

Supplementary Fig. 54 | The ECL−potential curves of 1 mM 10 μL EDA, BTTA, DAFB, DCTP, and TMT modified GCE. All the buffer solutions throughout the ECL experiments were 0.1 M PBS with 0.1 M KCl between 0 V and -3.0 V. PMT: 800 V, pH 7.5, potential scan rate: 100 mV s^{-1}.

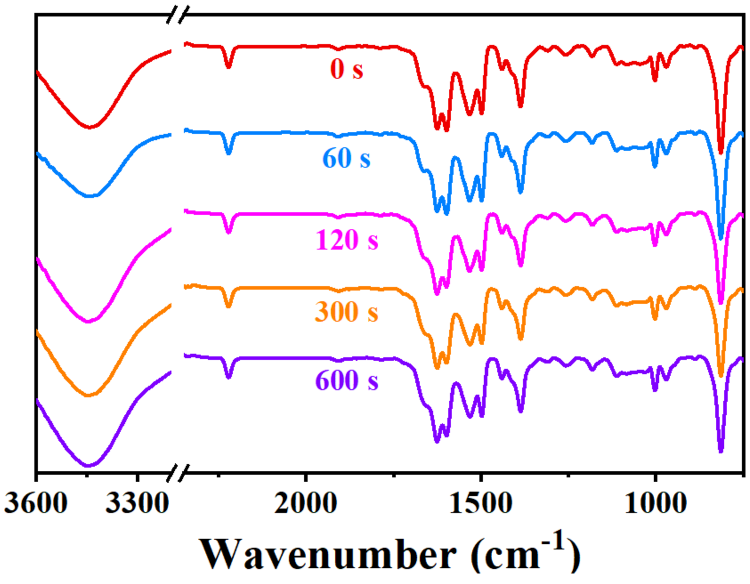

Supplementary Fig. 55 | FT-IR spectra of DAFB-DCTP during the 0~600s ECL cycles.

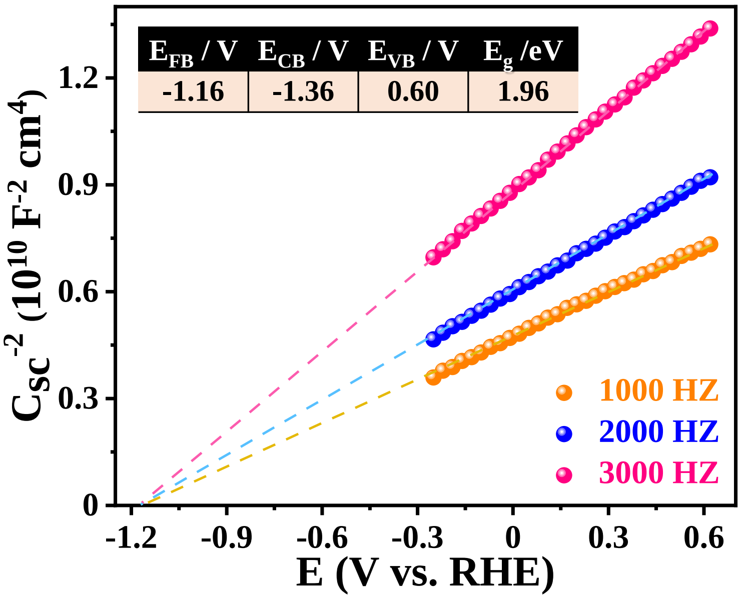

Supplementary Fig. 56 | Mott−Schottky plots of a DAFB-DCTP film at frequencies of 1, 2, and 3 kHz. The inset is band positions of DAFB-DCTP.

Liquid ^{1}H and ^{13}C NMR Spectra

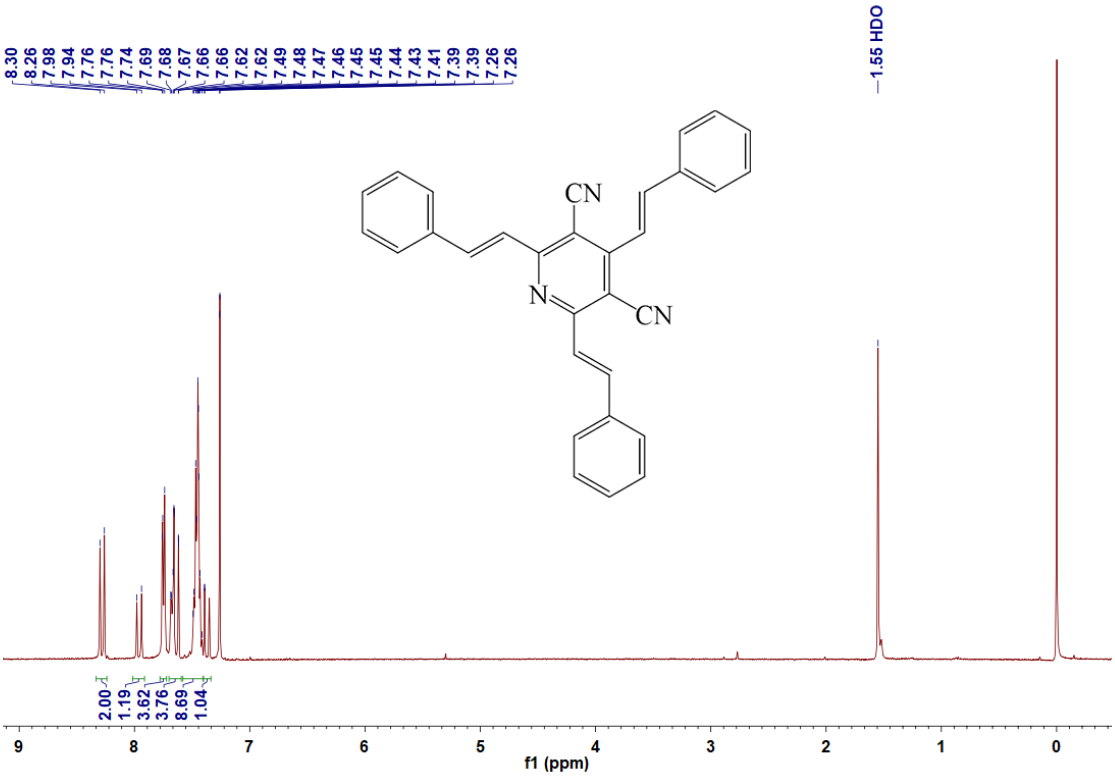

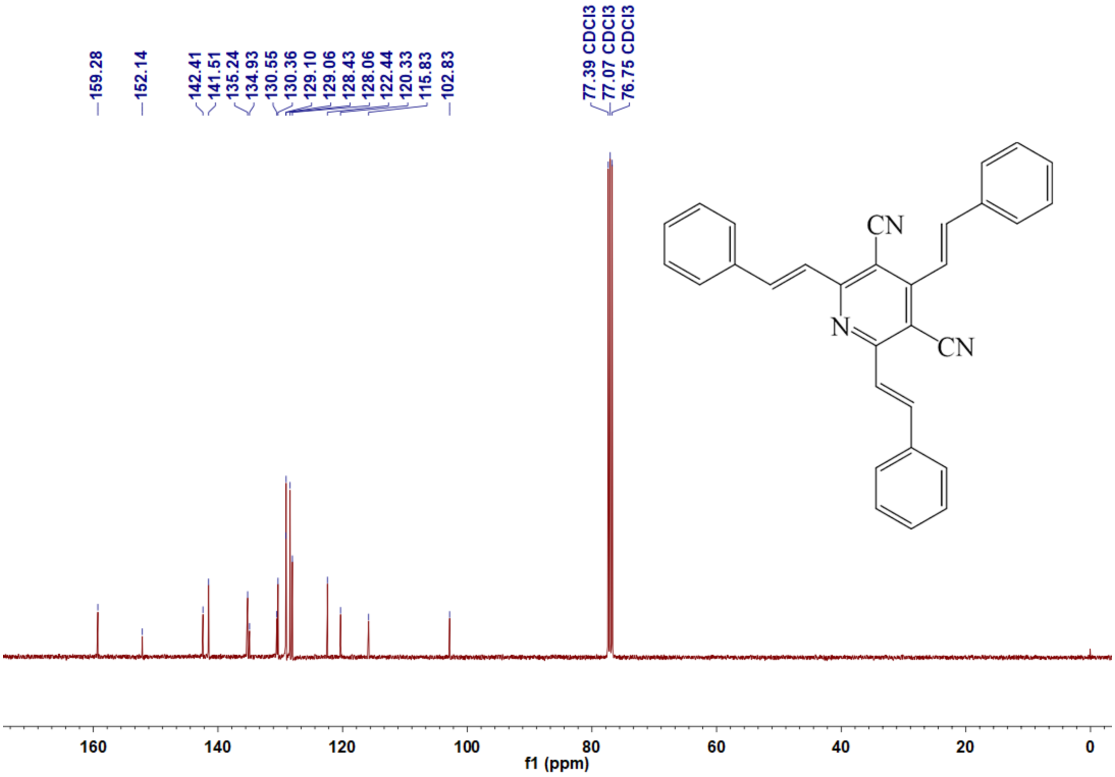

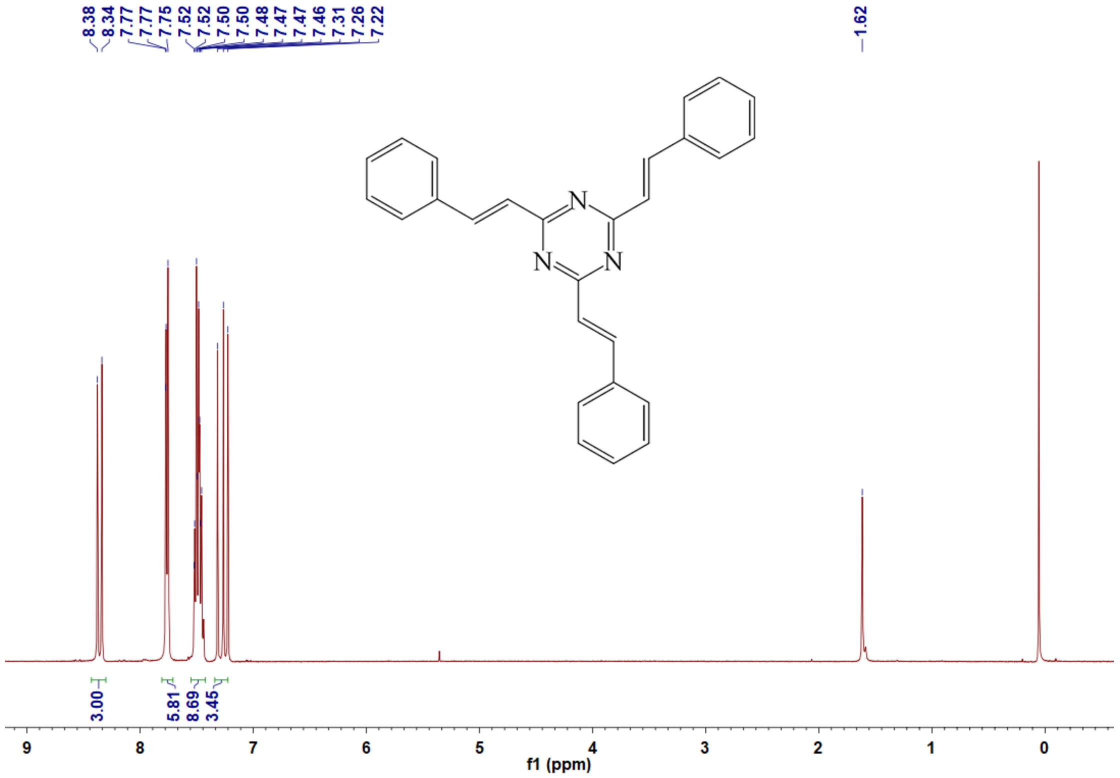
8.38
8.34
7.77
7.77
7.75
7.52
7.52
7.50
7.48
7.47
7.47
7.46
7.31
7.26
7.22
1.62
N
N
N
3.00
5.81
8.69
3.45
9
8
7
6
5
4
3
2
1
0
f1 (ppm)

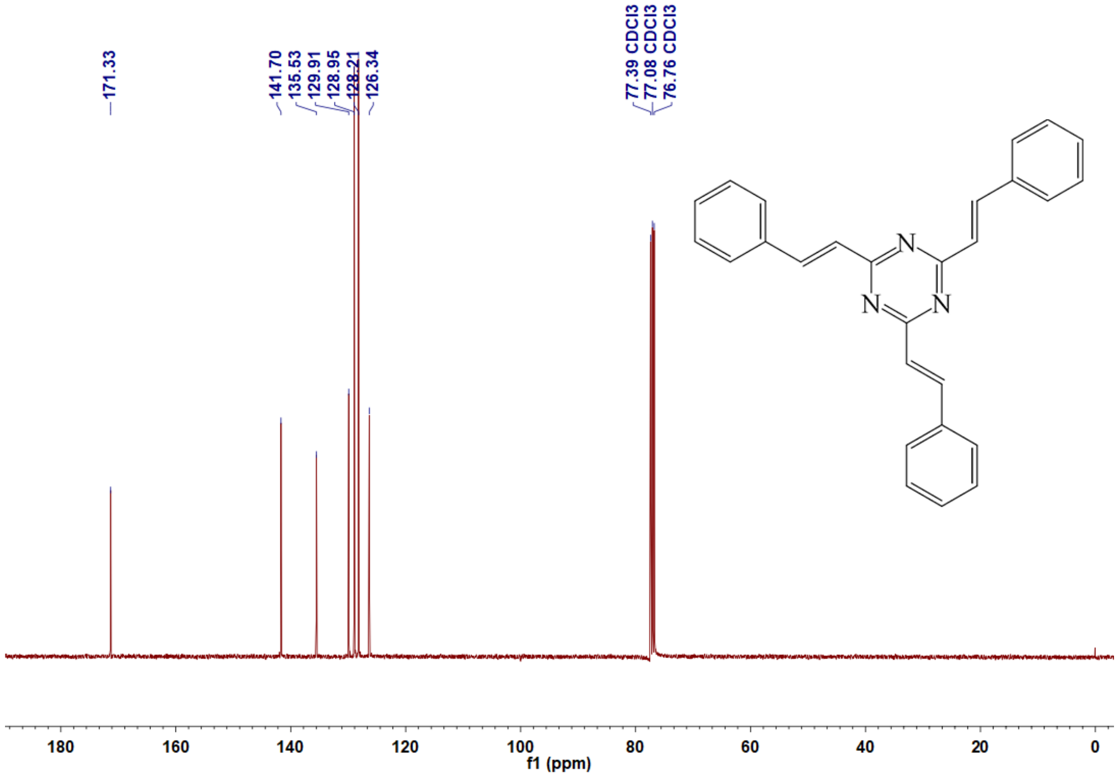
171.33
141.70
135.53
129.91
128.95
128.21
126.34
77.39 CDCl3
77.08 CDCl3
76.76 CDCl3
N
N
N
180
160
140
120
100
80
60
40
20
0
f1 (ppm)

Supplementary References

1. Xu, J. et al. An olefin-linked covalent organic framework as a flexible thin-film electrode for a high-performance micro-supercapacitor. *Angew. Chem. Int. Ed.* **58**, 12065-12069 (2019).

2. Lyu, H., Diercks, C. S., Zhu, C. & Yaghi, O. M. Crystalline olefin-linked covalent organic frameworks. *J. Am. Chem. Soc.* **141**, 6848-6852 (2019).

3. Bi, S. et al. Two-dimensional semiconducting covalent organic frameworks via condensation at arylmethyl carbon atoms. *Nat. Commun.* **10**, 2467 (2019).

4. Yang, Y., Niu, H., Xu, L., Zhang, H. & Cai, Y. Triazine functionalized fully conjugated covalent organic framework for efficient photocatalysis. *Appl. Catal. B-Environ.* **269**, 118799 (2020).

5. Zinna, F. et al. Circularly-polarized electrochemiluminescence from a chiral bispyrene organic macrocycle. *Angew. Chem. Int. Ed*. **58**, 6952-6956 (2019).

6. Long, Y.-T. & Meade, T. J. Advances in optical and electrochemical techniques for biomedical imaging. *Chem. Sci*. **11**, 6940-6941 (2020).

7. Qi, H., Chen, Y.-H., Cheng, C.-H. & Bard, A. J. Electrochemistry and electrogenerated chemiluminescence of three phenanthrene derivatives, enhancement of radical stability, and electrogenerated chemiluminescence efficiency by substituent groups. *J. Am. Chem. Soc*. **135**, 9041-9049 (2013).

8. Choi, J. P. et al. Electrogenerated chemiluminescence. 76. excited singlet state emission vs excimer emission in ter(9, 9-diarylfluorene)s. *J. Phys. Chem. B.* **107**, 14407-14413 (2003).

9. Wang, N. et al. Dual resonance energy transfer in triple-component polymer dots to enhance electrochemiluminescence for highly sensitive bioanalysis. *Chem. Sci*. **10**, 6815-6820 (2019).

10. Liang, Z. Liu, Z., Zhang, Q., Guo, Y. & Ma, Q. The high luminescent polydopamine nanosphere-based ECL biosensor with steric effect for MUC1 detection. *Chem. Eng. J*. **385**, 12385 (2020).

11. Luo, D. et al. Cathodic electrochemiluminescence of meso-tetra (4-carboxyphenyl) porphyrin/potassium peroxydisulfate system in aqueous media.

Electrochim. Acta. **151**, 42-49 (2015).

12. Fungo, F., Wong, K.-T., Ku, S.-Y., Hung, Y.-Y. & Bard, A. J. Electrogenerated chemiluminescence. 81. influence of donor and acceptor substituents on the ECL of a spirobifluorene-bridged bipolar system. *J. Phys. Chem. B.* **109**, 3984-3989 (2005).

13. Omer, K. M., Ku, S.-Y., Wong, K.-T. & Bard, A. J. Green electrogenerated chemiluminescence of highly fluorescent benzothiadiazole and fluorene derivatives. *J. Am. Chem. Soc*. **131**, 10733-10741 (2009).

14. Soulsby, L. C. et al. Co-reactant and annihilation electrogenerated chemiluminescence of $[Ir(df\text{-}ppy)_2(ptb)]^+$ derivatives. *ChemElectroChem.* **7**, 1889-1896 (2020).

15. O'Reilly, E. J., Keyes, T. E., Forster, R. J. & Dennanyet L. Insights into electrochemiluminescent enhancement through electrode surface modification. *Analyst*. **138**, 677-682 (2013).

16. Molapo, K. M. et al. High efficiency electrochemiluminescence from polyaniline: ruthenium metal complex films. *Electrochem. Commun.* **148**, 95-98 (2014).

17. Wang, F. et al. Intrinsic “vacancy point defect” induced electrochemiluminescence from coreless supertetrahedral chalcogenide nanocluster. *J. Am. Chem. Soc*. **138**, 7718-7724 (2016).

18. Liu, Y., Wang, M., Nie, Y., Zhang, Q. & Ma, Q. Sulfur regulated boron nitride quantum dots electrochemiluminescence with amplified surface plasmon coupling strategy for BRAF gene detection. *Anal. Chem*. **91**, 6250-6258 (2019).
